# Supplementary material for: Transgenerational transmission of reproductive and metabolic dysfunction in the male progeny of polycystic ovary syndrome
Source: Cell Rep Med. 2023 May 5;4(5):101035. doi: 10.1016/j.xcrm.2023.101035 (PMC10213875; doi:10.1016/j.xcrm.2023.101035)
Supplement: Document S1. Tables S1 and S2 and Figures S1–S3 [file mmc1.pdf]

Cell Reports Medicine, Volume *4*

Supplemental information

Transgenerational transmission of reproductive and metabolic dysfunction in the male progeny of polycystic ovary syndrome

Sanjiv Risal, Congru Li, Qing Luo, Romina Fornes, Haojiang Lu, Gustaw Eriksson, Maria Manti, Claes Ohlsson, Eva Lindgren, Nicolas Crisosto, Manuel Maliqueo, Barbara Echiburú, Sergio Recabarren, Teresa Sir Petermann, Anna Benrick, Nele Brusselaers, Jie Qiao, Qiaolin Deng, and Elisabet Stener-Victorin

Cell Reports Medicine

Supplemental Information

Transgenerational transmission of reproductive and metabolic dysfunction in the male progeny of polycystic ovary syndrome

Sanjiv Risal, Congru Li, Qing Luo, Romina Fornes, Haojiang Lu, Gustaw Eriksson, Maria Manti, Claes Ohlsson, Eva Lindgren, Nicolas Crisosto, Manuel Maliqueo, Barbara Echiburú, Sergio Recabarren, Teresa Sir Petermann, Anna Benrick, Nele Brusselaers, Jie Qiao, Qiaolin Deng, Elisabet Stener-Victorin

Supplemental Data to:

Transgenerational transmission of reproductive and metabolic dysfunction in the male progeny of polycystic ovary syndrome

Sanjiv Risal[1,13], Congru Li[1,2,13], Qing Luo[1,13], Romina Fornes[1,3,13], Haojiang Lu[1], Gustaw Eriksson[1], Maria Manti[1], Claes Ohlsson[4,5], Eva Lindgren[1], Nicolas Crisosto[6,7], Manuel Maliqueo[6], Barbara Echiburú[6], Sergio Recabarren[8], Teresa Sir Petermann[6], Anna Benrick[9,10], Nele Brusselaers[3,11], Jie Qiao[2], Qiaolin Deng[1, 12, 14], Elisabet Stener-Victorin[1, 15]

1 Department of Physiology and Pharmacology, Karolinska Institutet, Stockholm, Sweden

2 Center of Reproductive Medicine, Department of Obstetrics and Gynecology, Peking University Third Hospital, Beijing 100191, China

3 Department of Microbiology, Tumor and Cell Biology, Karolinska Institutet, Stockholm, Sweden

4 Centre for Bone and Arthritis Research, Department of Internal Medicine and Clinical Nutrition, Institute of Medicine, Sahlgrenska Academy, University of Gothenburg, Gothenburg, Sweden

5 Department of Drug Treatment, Region Västra Götaland, Sahlgrenska University Hospital, Gothenburg, Sweden

6 Endocrinology and Metabolism Laboratory, West Division, School of Medicine, University of Chile, Carlos Schachtebeck 299, Interior Quinta Normal, Santiago, Chile

7 Endocrinology Unit, Department of Medicine, Clínica Alemana de Santiago, Faculty of Medicine, Clinica Alemana, Universidad del Desarrollo, Santiago, Chile

8 Laboratory of Animal Physiology and Endocrinology, Faculty of Veterinary Sciences, University of Concepción, Chillán, Chile

9 Department of Physiology, Sahlgrenska Academy, University of Gothenburg, Gothenburg, Sweden

10 School of Health Sciences, University of Skövde, Skövde, Sweden

11 Global Health Institute, Antwerp University, Antwerp, Belgium

12 Center for Molecular Medicine, Karolinska University Hospital, Stockholm, Sweden

[13] **These authors contributed equally.**

[14] **Corresponding author**
Qiaolin Deng: qiaolin.deng@ki.se
Karolinska Institutet, Department of Physiology and Pharmacology
Center for molecular medicine
171 77 Stockholm, Sweden

[15] **Corresponding author and lead contact**
Elisabet Stener-Victorin: elisabet.stener-victorin@ki.se
Karolinska Institutet, Department of Physiology and Pharmacology
171 77 Stockholm, Sweden

Table S1. Maternal and pregnancy characteristics of women with and without polycystic ovary syndrome (PCOS) and exposure to metformin (Met) and perinatal characteristics of their sons between 2006 to 2016 in a Swedish register-based cohort study. **Related to figure 1.**

	**Control**		**PCOS**		**PCOS+/Met-**		**PCOS+/Met+**	
	**n**	**%**	**n**	**%**	**n**	**%**	**n**	**%**
	**457,447**	**%**	**9,828**	**%**	**9,663**		**165**	
Maternal age, in years								
< 25	66,280	14.5	1,341	13.6	1,325	13.7	16	9.7
25-29	135,271	29.6	3,262	33.2	3,210	33.2	52	31.5
30-34	156,353	34.2	3,425	34.8	3,372	34.9	53	32.1
≥ 35	99,543	21.8	1,800	18.3	1,756	18.2	44	26.7
Maternal body mass index, in kg/m^2								
<20	47,168	10.3	736	7.5	731	7.6	5	3.0
20-24.9	239,774	52.4	3,780	38.5	3,751	38.8	29	17.6
25-29.9	114,682	25.1	2,730	27.8	2,689	27.8	41	24.8
≥30	55,823	12.2	2,582	26.3	2,492	25.8	90	54.5
Parity								
Multiparous	255,389	55.8	4,616	47.0	4,543	47.0	73	44.2
Primiparous	202,058	44.2	5,212	53.0	5,120	53.0	92	55.8
Cigarrette consumption								
yes	28,249	6.2	583	5.9	4,543	33.3	16	9.7
no	429,198	93.8	9,245	94.1	9,096	66.7	149	90.3
Assisted reproduction								
yes	12,739	2.8	1,086	11.1	1,069	11.1	17	10.3
no	444,708	97.2	8,742	88.9	8,594	88.9	148	89.7
Diabetes								
yes	8,935	2.0	528	5.4	490	5.1	38	23.0
no	448,512	98.0	9,300	94.6	9,173	94.9	127	77.0
Size for gestational age								
Adequate for gestational age	432,251	94.5	9,137	93.0	8,990	93.0	147	89.1
Small for gestational age	10,025	2.2	209	2.1	204	2.1	5	3.0
Large for gestational age	15,171	3.3	482	4.9	469	4.9	13	7.9
Cesarean section								
yes	78,626	17.2	2,081	21.2	2,034	21.0	47	28.5
no	378,821	82.8	7,747	78.8	7,629	79.0	118	71.5

Preterm birth								
yes	21,217	4.6	622	6.3	612	6.3	10	6.1
no	436,230	95.4	9,206	93.7	9,051	93.7	155	93.9
Apgar <07 at 5 minutes								
yes	5,229	1.1	153	1.6	151	1.6	2	1.2
no	452,218	98.9	9,675	98.4	9,512	98.4	163	98.8

Table S2. Clinical. endocrine and metabolic features of sons of control and PCOS women in the Chilean case-control study. **Related to figure 1.**

	Tanner I			Tanner II - III			Tanner IV - V		
	Control	PCOS	*P*	Control	PCOS	*P*	Control	PCOS	*P*
Age (y)	9.0 (8.3 - 9.6) (20)	9.1 (8.4 - 9.6) (23)	0.541	10.3 (9.8 - 11.8) (31)	10.8 (10.3 - 11.6) (26)	0.442	14.6 (13.2 - 16.4) (42)	15.3 (13.7 - 16.7) (29)	0.289
Weight (kg)	35.6 (26.6 - 38.7) (20)	33.5 (30 - 42.0) (23)	0.846	39.1 (34.6 - 48.0) (31)	40.8 (35.9 - 54.6) (26)	0.400	60.1 (52.9 - 77.4) (42)	65.1 (56.5 - 78.5) (29)	0.281
Height (m)	1.3 (1.3 - 1.4) (20)	1.4 (1.3 - 1.4) (23)	0.573	1.4 (1.4 - 1.5) (31)	1.4 (1.4 - 1.5) (26)	0.500	1.7 (1.6 - 1.7) (42)	1.7 (1.6 - 1.7) (29)	0.091
BMI (kg/m^2)	19.8 (16.4 - 22.6) (20)	18.7 (17.3 - 22.4) (23)	0.850	20.2 (18.2 - 23.1) (31)	21.0 (18.4 - 24.8) (26)	0.421	22.9 (19.8 - 26.4) (42)	24.5 (20.2 - 27.1) (29)	0.533
Z-Score BMI	1.6 (-0.1 - 2.4) (20)	1.4 (0.4 - 2.6) (23)	0.581	1.5 (0.6 - 2.1) (31)	1.6 (0.8 - 2.5) (26)	0.330	1.2 (0.5 - 1.8) (42)	1.5 (0.3 - 2.1) (29)	0.648
Obesity (%)	40.0	39.1	1.000	29.0	34.6	0.777	16.7	24.1	0.547
Waist (cm)	63.3 (59.3 - 68) (20)	62.5 (59.6 - 70) (20)	0.968	66.0 (61.0 - 75.0) (31)	70.5 (65.8 - 79.3) (26)	0.111	74.5 (70.0 - 84.3) (42)	79.0 (72.8 - 87.5) (29)	0.097
Hip (cm)	71.0 (62.1 - 77.6) (20)	68 (64.3 - 77.3) (20)	0.560	72.0 (65.0 - 82.0) (31)	74.8 (69.9 - 84) (26)	0.431	84.0 (80.0 - 92.6) (42)	87.0 (80.8 - 93.5) (29)	0.264
Endocrine parameters									
SHBG (nmol/l)	56.5 (36.7 - 85.8) (20)	47.3 (35.2 - 66.7) (22)	0.268	45.9 (31.1 - 69.1) (30)	50.0 (29.7 - 72.5) (25)	1.000	23.0 (16.5 - 39.6) (41)	22.6 (16.6 - 30.5) (28)	0.582
LH (mIU/ml)	0.1 (0.1 - 0.2) (20)	0.1 (0.1 - 0.9) (20)	0.245	0.8 (0.2 - 2.5) (28)	1.8 (0.2 - 3.4) (24)	0.533	5.1 (2.8 - 7.8) (42)	4.5 (3.4 - 6.5) (27)	0.623
FSH (mIU/ml)	0.3 (0.1 - 0.7) (20)	0.5 (0.2 - 0.7) (21)	0.375	0.4 (0.1 - 0.9) (29)	1.0 (0.3 - 2.2) (24)	**0.038**	1 (0.3 - 2.2) (42)	0.9 (0.4 - 3.6) (27)	0.503
A4 (ng/ml)	0.3 (0.2 - 0.6) (20)	0.5 (0.3 - 0.7) (21)	0.111	0.6 (0.3 - 0.9) (29)	0.7 (0.6 - 1.1) (25)	0.070	1.3 (0.9 - 1.9) (42)	1.6 (1.2 - 2.4) (28)	**0.039**
T (ng/ml)	0.1 (0.1 - 0.2) (20)	0.1 (0.1 - 0.2) (21)	0.102	0.2 (0.1 - 0.9) (30)	0.3 (0.2 - 1.2) (25)	0.314	3.7 (2.9 - 4.7) (40)	4.0 (3.1 - 6.1) (27)	0.184
AMH (pmol/L)	528.5 (434.4 - 780.7) (20)	549.4 (458.4 - 726.3) (20)	0.626	411.6 (200.1 - 528.6) (30)	440.8 (154.7 - 514.5) (24)	0.287	67.8 (42.6 - 119.6) (41)	62.6 (39.3 - 119.8) (28)	0.678
FAI	0.7 (0.3 - 1.1) (20)	1.0 (0.8 - 1.7) (21)	0.054	2.4 (0.9 - 5.4) (30)	2.9 (1.4 - 12.3) (25)	0.917	44.9 (32.0 - 92.8) (40)	68.1 (52.5 - 86.7) (27)	0.135
Metabolic parameters									
Fating glucose (mg/dl)	80.0 (77.3 - 85) (20)	84.0 (78.0 - 89.0) (23)	0.215	84.0 (76.0 - 90.0) (31)	87 (80.8 - 93.5) (26)	0.111	84.5 (77.0 - 92.0) (42)	84.0 (79.0 - 87.0) (29)	0.680
Fasting insulin (uIU/ml)	7.9 (5.4 - 12.4) (20)	7.0 (5.2 - 11.9) (23)	0.715	9.5 (7.2 - 13.9) (31)	9.3 (7.1 - 12.7) (26)	0.949	12.8 (9.2 - 18) (41)	12.8 (7.5 - 18.5) (29)	0.839
HOMA	1.5 (1.1 - 2.6) (20)	1.6 (1.1 - 2.5) (23)	0.903	2.0 (1.5 - 2.9) (31)	1.9 (1.5 - 2.7) (26)	0.898	2.7 (1.9 - 3.8) (41)	2.6 (1.7 - 3.6) (29)	0.716
AUC glucose (mg/dl/2-h)	11340 (10413.8 - 13196.3) (20)	11865 (10365 - 14280) (23)	0.466	12585 (11370 - 14700) (31)	12967.5 (11782.5 - 14602.5) (26)	0.564	12180 (10927.5 - 13882.5) (33)	12412.5 (11036.3 - 13301.3) (20)	0.761
AUC insulin (uIU/ml/2-h)	4949.1 (2328.5 - 8004.2) (20)	4341.3 (2990.3 - 5933.6) (23)	0.715	4827.3 (2946.6 - 9672.9) (31)	5916.8 (4032.7 - 10748.9) (26)	0.543	7068 (5124.6 - 12910.7) (33)	7975.3 (5006 - 11510) (20)	0.971
Triglycerides (mg/dl)	107.5 (86.3 - 131.8) (20)	104.0 (81.0 - 137.0) (23)	0.456	106.0 (82.0 - 141.0) (31)	125.5 (88.5 - 150.8) (26)	0.247	109.5 (77.3 - 138.3) (42)	97 (83.0 - 138.0) (29)	0.478
Cholesterol (mg/dl)	141.0 (133.3 - 163.5) (20)	155.0 (129.0 - 176.0) (23)	0.348	140.0 (130.0 - 159.0) (31)	164.0 (141.8 - 179.0) (26)	**0.008**	118.5 (103.5 - 137.3) (42)	134.0 (118.0 - 164.0) (29)	**0.015**
HDL-Cholesterol (mg/dl)	43.8 (39.4 - 52.8) (20)	40.1 (36.7 - 49.7) (23)	0.113	41.8 (37.2 - 45.1) (31)	42.0 (38.0 - 52.0) (26)	0.665	36.1 (29.5 - 43.0) (42)	38.6 (33.5 - 44.0) (29)	0.390
LDL-Cholesterol (mg/dl)	74.1 (57.4 - 82.2) (20)	88.9 (70.1 - 107.5) (23)	0.084	79.6 (57.6 - 92.9) (31)	92.6 (78.5 - 103.4) (26)	**0.037**	54.0 (45.4 - 71.7) (41)	74.6 (56.3 - 101.1) (29)	**0.005**

Data are expressed as median (p25 - p75). Brackets show the number of observations. Differences were calculated by Student t' test or Mann Whitney test depending on normal distribution. AUC: Area Under the Curve; BMI: Body mass index; FAI: free androgen index.

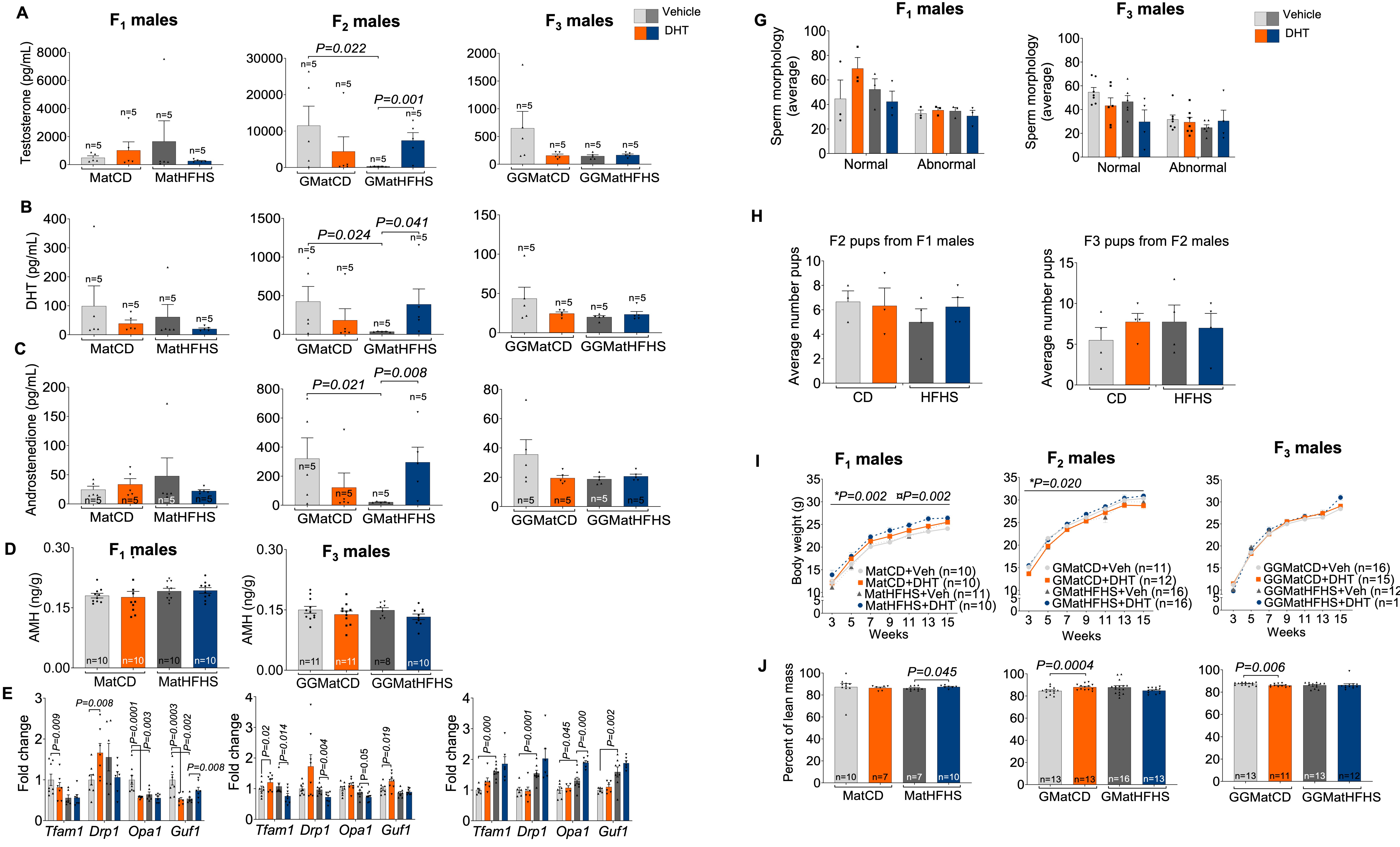

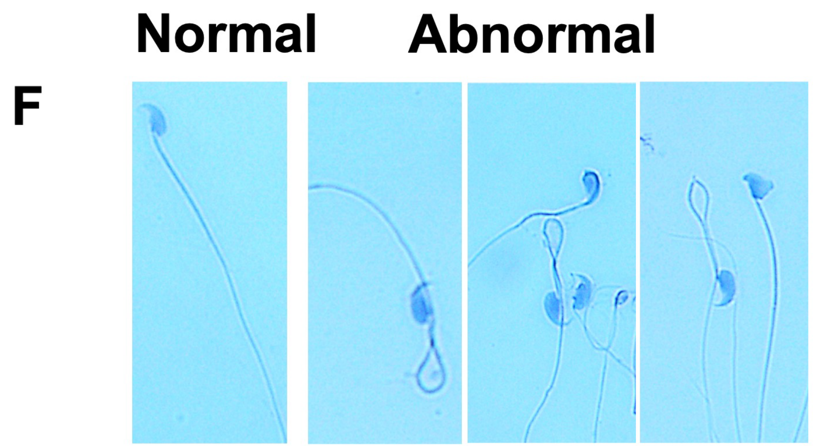

Supplemental Figure 1. Maternal obesity and prenatal androgen do not affect serum steroid levels and sperm morphology, mitochondrial gene expression, and body weight in male offspring. Related to figure 2.
Serum (A) testosterone, (B) dihydrotestosterone and (C) androstenedione levels measured by gas-chromatography mass spectrometry (GC-MS/MS) in F1, F2 and F3 male offspring.
(CD+Veh (F1 n=5, litters=5; F2 n=5, litters=4; F3 n=5, litters=4), CD+DHT (F1 n=5, litters=5; F2 n=5, litters=4, F3 n=5, litters=4), HFHS+Veh (F1 n=5, litters=5; F2 n=5, litters=4, F3 n=5, litters=4), HFHS+DHT (F1 n=5, litters=5; F2 n=5, litters=4; F3 n=5, litters=4). (D) mRNA expression levels of mitochondrial genes: Tfam1, Drp1, Opa1, and Guf1 in testis of F1, F2, and F3 male offspring.
CD+Veh (F1 n=8, litters=5; F2 n=8, litters=4 F3 n=6, litters=3), CD+DHT (F1, n=7, litters=5; F2 n=8, litters=4, F3 n=6, litters=4), HFHS+Veh (F1 n=6, litters=5; F2 n=7, litters=4, F3 n=8, litters=4), HFHS+DHT (F1, n=7, litters=5; F2 n=8, litters=4;, F3 n=6, litters=4).
(E) Representative images of normal and abnormal sperm morphology considered for analysis. (F) Average number of normal and abnormal sperm (expressed in percentages) of F1 and F3 male offspring.
(G) Average total number of pups sired by F1 and F2 males. Body weight development in (H) F1, F2, and F3 adult male offspring from 3 to15 weeks of age.
CD+Veh (F1 n=10, litters=5; F2 n=11, litters=4; F3 n=16, litters=4), CD+DHT (F1 n=10, litters=5; F2 n=12, litters=4; F3 n=15, litters=4), HFHS+Veh, (F1 n=11, litters=5; F2 n=16, litters=4; F3 n=12, litters=4), HFHS+DHT (F1 n=10, litters=7; F2 n=16, litters=4; F3 n=13, litters=4).
(i) Percent lean mass normalized with body weight (grams) in F1, F2, and F3 adult male offspring.
CD+Veh (F1 n=10, litters=5; F2 n=13, litters=4; F3 n=13, litters=4), CD+DHT (F1 n=7, litters=5; n=13; F2 litters=4, F3 n=11, litters=4), HFHS+Veh (F1 n=10, litters=5; F2 n=16, litters=4, F3 n=13, litters=4), HFHS+DHT, (F1 n=7, litters=4. F2 n=13, litters=4; F3 n=12, litters=4).
Lineage: CD+Veh = maternal control diet + vehicle exposure; CD+DHT = maternal control diet + dihydrotestosterone exposure; HFHS+Veh = maternal high fat high sucrose diet + vehicle exposure.
Comparison between the groups were performed using linear mixed-effects models, non-repeated measures (ANODE, R package car) except for panel H) linear mixed-effects models with repeated measure was used.
*=CD+Veh vs CD+DHT; ¤=HFHS+Veh vs HFHS+DHT. Each dot represents one offspring mouse. All data are presented as mean ± s.e.m.

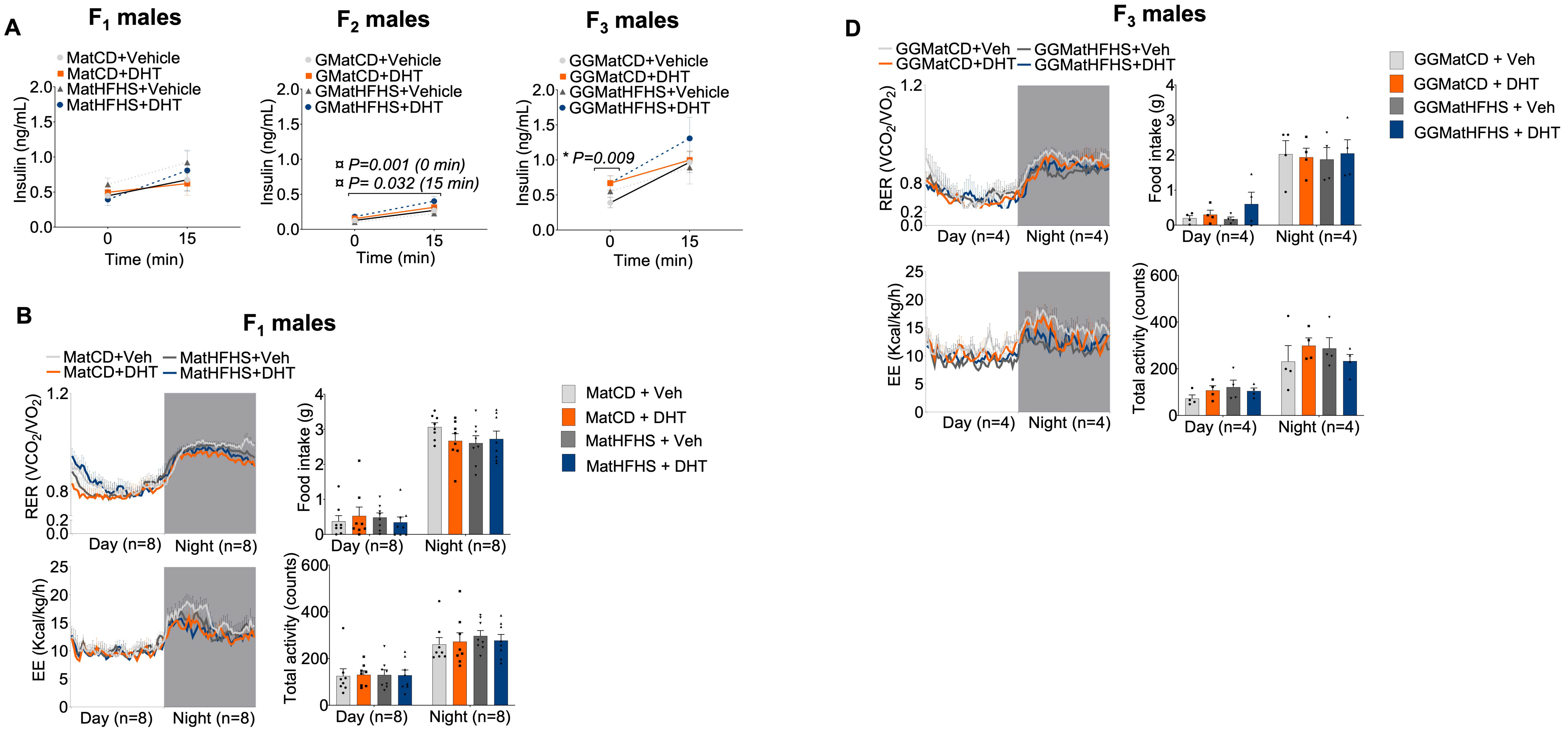

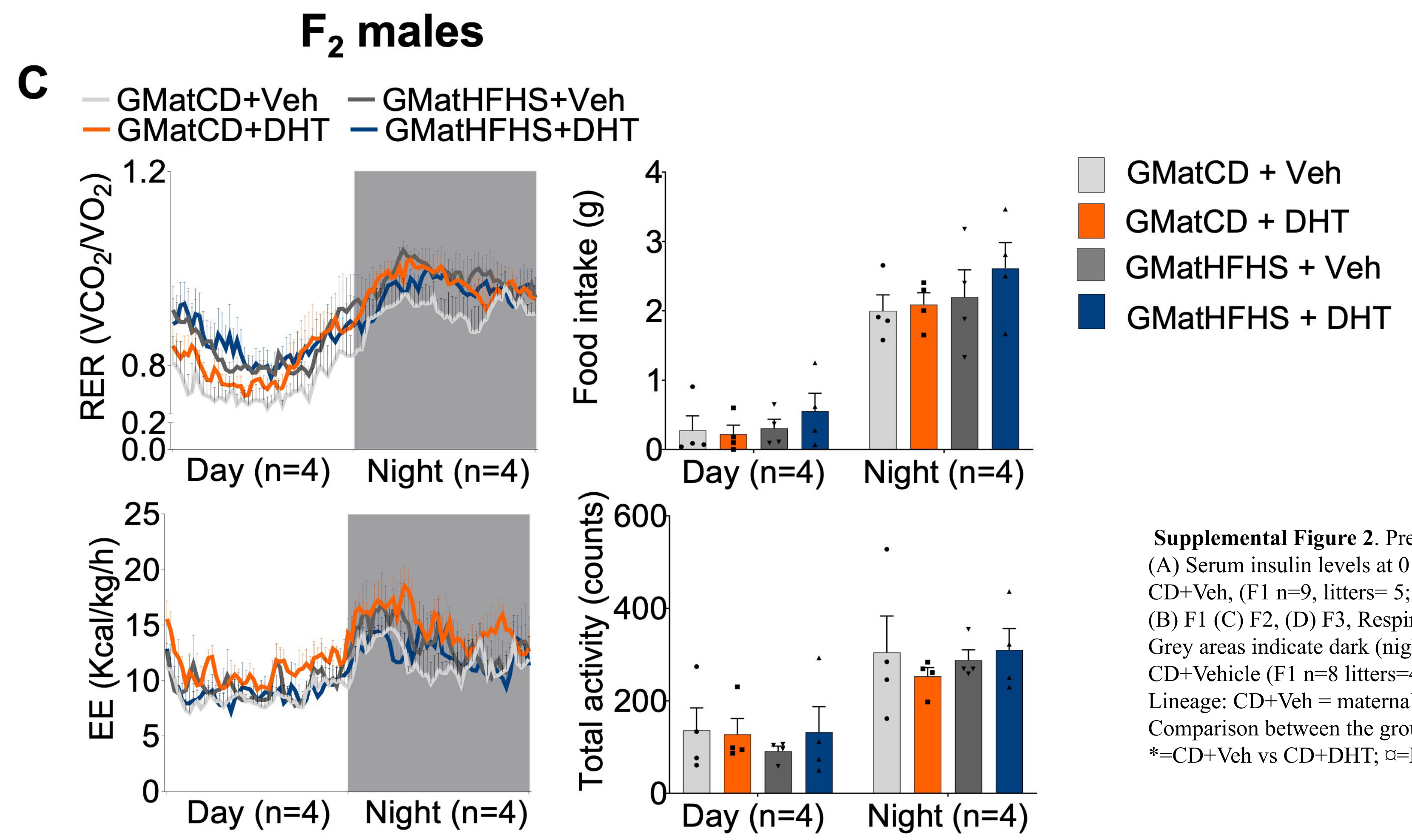

Supplemental Figure 2. Prenatal androgen and maternal obesity exposure cause transgenerational impaired energy balance in male offspring. Related to figure 3.
(A) Serum insulin levels at 0 and 15 minutes before and after oral glucose challenge.
CD+Veh, (F1 n=9, litters= 5; F2 n=9, litters=4; F3 n=12, litters=4), CD+DHT (F1 n=7, litters=4, F2 n=5, litters=4, F3 n=8, litters=4), HFHS+Veh (F1 n=9 litters=5; F2 n=9, litters=4; F3 n=12, litters=4) HFHS+DHT (F1 n=7, litters=4; F2 n=5, litters=4. F3 n=8, litters=4).
(B) F1 (C) F2, (D) F3, Respiratory exchange ratio (RER; VCO2/VO2) and energy expenditure (EE) was measured by indirect calorimetry by using the TSE system in F1 to F3 adult male offspring.
Grey areas indicate dark (night) periods (6 PM to 6 AM). Bar graph represents RER, EE, food intake, and total activity average values during day and night.
CD+Vehicle (F1 n=8 litters=4; F2 n=4, litters= 4; F3 n=4, litters=4), CD+DHT (F1 n=8, litters=4; F2 n=4, litters=4; F3 n=4, litters=4), HFHS+Veh (F1 n=8, litters=4; F2 n=4, litters=4; F3 n=4, litters=4), and HFHS+DHT (F1 n=8, litters=5; F2 n=4, litters=4; F3 n=4, litters=4).
Lineage: CD+Veh = maternal control diet + vehicle exposure; CD+DHT = maternal control diet + dihydrotestosterone exposure; HFHS+Veh = maternal high fat high sucrose diet + vehicle exposure.
Comparison between the groups were performed using linear mixed-effects models, non-repeated measures (ANODE, R package car) except for panel H) linear mixed-effects models with repeated measure was used.
*=CD+Veh vs CD+DHT; ¤=HFHS+Veh vs HFHS+DHT. Each dot represents one offspring mouse. All data are presented as mean ± s.e.m.

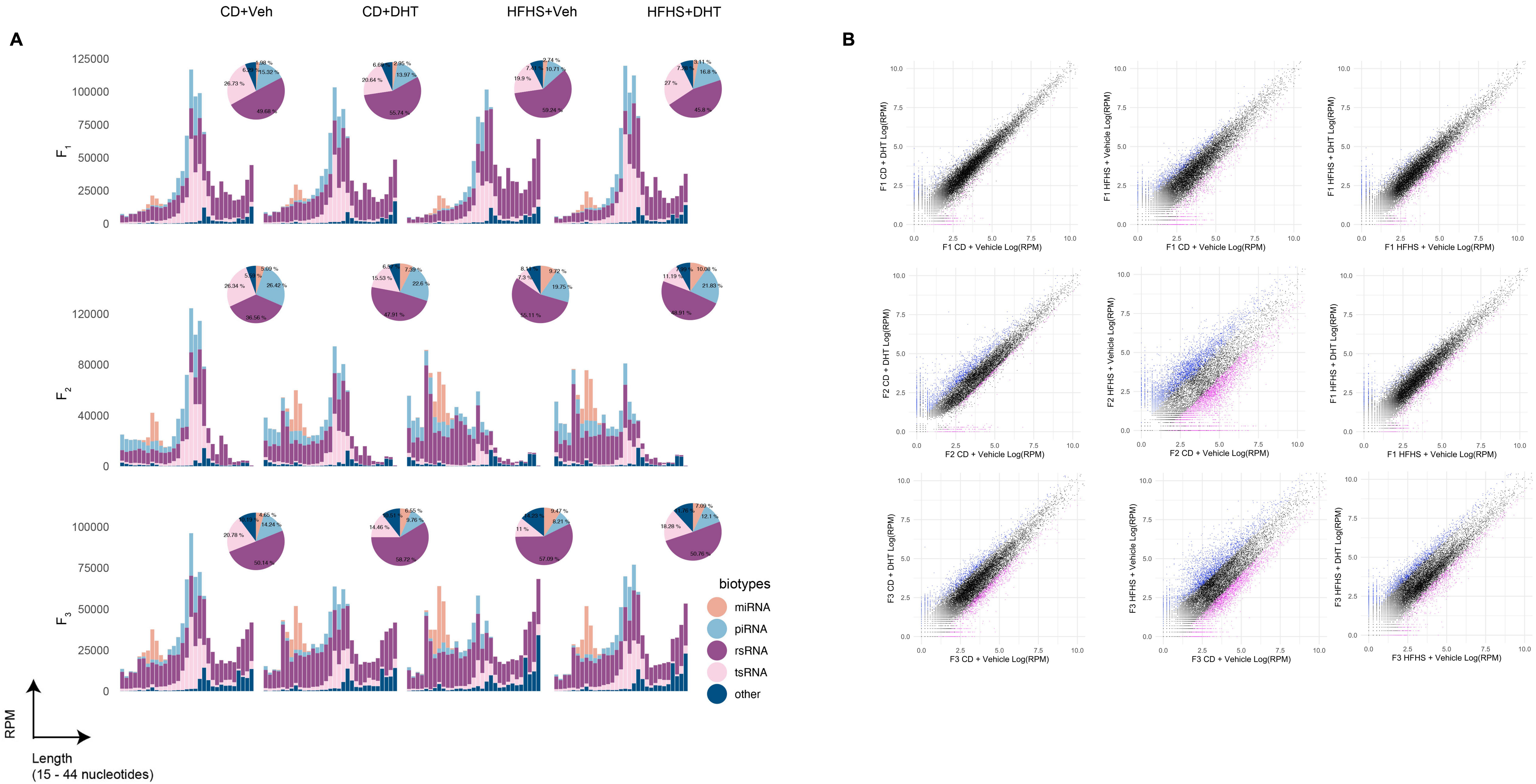

Supplemental Figure 3. Size distribution and expression of sncRNAs in sperm. Related to figure 4.
(A) The size distribution of miRNA, piRNA, rsRNA, tsRNA and other biotypes in different lineages in F1 and F3 male. Average RPM (reads per million) values in bar plots and RPM proportion (%) in pie charts.
(B) Scatterplot analysis showing average RPM values of different lineages as indicated in x-axis and y-axis for differentially expressed sncRNA. Down (magenta) and up (blue) regulated sncRNAs.
CD+Veh (F1 n=4, litters=4; F2 n=4, litters= 4; F3 n=4, litters=4), CD+DHT (F1 n=3, litters=3; F2 n=4, litters=4; F3 n=3, litters=3), HFHS+Veh (F1 n=4, litters=4; F2 n=4, litters=4; F3 n=4, litters=4), and HFHS+DHT (F1 n=4, litters=4; F2 n=4, litters=4; F3 n=4, litters=4).
