# Supplementary material for: Transgenerational transmission of reproductive and metabolic dysfunction in the male progeny of polycystic ovary syndrome
Source: Cell Rep Med. 2023 May 5;4(5):101035. doi: 10.1016/j.xcrm.2023.101035 (PMC10213875; doi:10.1016/j.xcrm.2023.101035)
Supplement: Document S2. Article plus supplemental information [file mmc10.pdf]

Cell Reports
Medicine

Article

Transgenerational transmission of reproductive and metabolic dysfunction in the male progeny of polycystic ovary syndrome

Graphical abstract

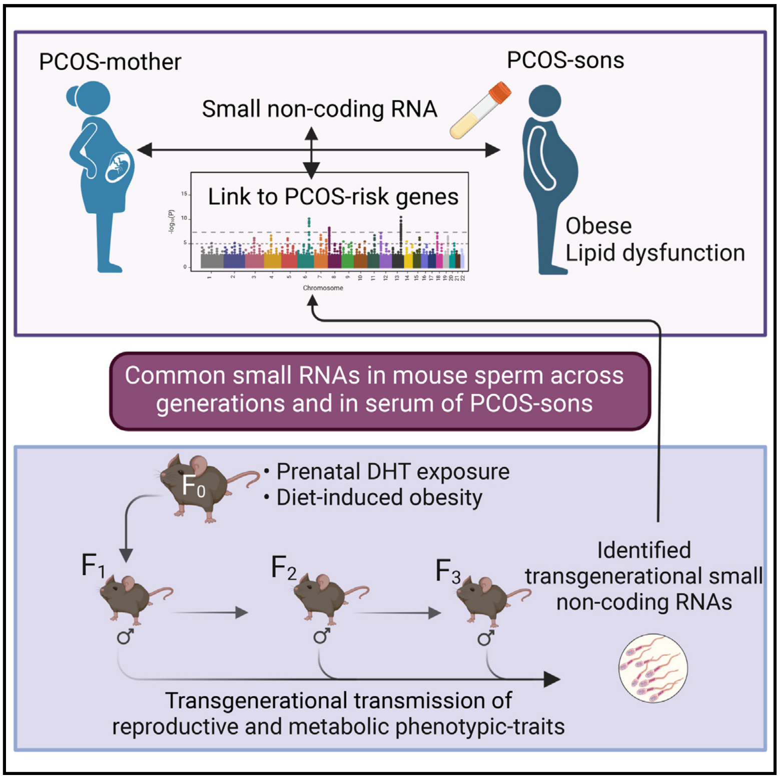

Highlights

- PCOS-sons are often obese and have dyslipidemia
- miRNAs altered in the serum of PCOS-sons and women with PCOS targets PCOS-risk genes
- Small RNAs present in sperm imply transgenerational transmission of phenotype in mice
- Shared miRNAs between mouse sperm of F_1–F_3 generations and human serum are revealed

Authors

Sanjiv Risal, Congru Li, Qing Luo, ..., Jie Qiao, Qiaolin Deng, Elisabet Stener-Victorin

Correspondence

qiaolin.deng@ki.se (Q.D.), elisabet.stener-victorin@ki.se (E.S.-V.)

In brief

Risal et al. found that the sons of women affected by polycystic ovary syndrome (PCOS) are frequently obese and dyslipidemic. Male descendants of obese or androgen-exposed mothers also exhibit reproductive and metabolic problems across generations, mediated by sperm small RNAs dysregulation. Common predicted small RNA targets are suggested in PCOS-affected mice and PCOS-sons' serum.

Risal et al., 2023, Cell Reports Medicine *4*, 101035
May 16, 2023 © 2023 The Author(s).
https://doi.org/10.1016/j.xcrm.2023.101035

CellPress

Cell Reports Medicine

CellPress
OPEN ACCESS

Article

Transgenerational transmission of reproductive and metabolic dysfunction in the male progeny of polycystic ovary syndrome

Sanjiv Risal,[1,13] Congru Li,[1,2,13] Qing Luo,[1,13] Romina Fornes,[1,3,13] Haojiang Lu,[1] Gustaw Eriksson,[1] Maria Manti,[1] Claes Ohlsson,[4,5] Eva Lindgren,[1] Nicolas Crisosto,[6,7] Manuel Maliqueo,[6] Barbara Echiburú,[6] Sergio Recabarren,[8] Teresa Sir Petermann,[6] Anna Benrick,[9,10] Nele Brusselaers,[3,11] Jie Qiao,[2] Qiaolin Deng,[1,12,*] and Elisabet Stener-Victorin[1,14,*]

[1]Department of Physiology and Pharmacology, Karolinska Institutet, Stockholm, Sweden
[2]Center of Reproductive Medicine, Department of Obstetrics and Gynecology, Peking University Third Hospital, Beijing 100191, China
[3]Department of Microbiology, Tumor and Cell Biology, Karolinska Institutet, Stockholm, Sweden
[4]Centre for Bone and Arthritis Research, Department of Internal Medicine and Clinical Nutrition, Institute of Medicine, Sahlgrenska Academy, University of Gothenburg, Gothenburg, Sweden
[5]Department of Drug Treatment, Region Västra Götaland, Sahlgrenska University Hospital, Gothenburg, Sweden
[6]Endocrinology and Metabolism Laboratory, West Division, School of Medicine, University of Chile, Carlos Schachtebeck 299, Interior Quinta Normal, Santiago, Chile
[7]Endocrinology Unit, Department of Medicine, Clínica Alemana de Santiago, Faculty of Medicine, Clinica Alemana, Universidad del Desarrollo, Santiago, Chile
[8]Laboratory of Animal Physiology and Endocrinology, Faculty of Veterinary Sciences, University of Concepción, Chillán, Chile
[9]Department of Physiology, Sahlgrenska Academy, University of Gothenburg, Gothenburg, Sweden
[10]School of Health Sciences, University of Skövde, Skövde, Sweden
[11]Global Health Institute, Antwerp University, Antwerp, Belgium
[12]Center for Molecular Medicine, Karolinska University Hospital, Stockholm, Sweden
[13]These authors contributed equally
[14]Lead contact
*Correspondence: qiaolin.deng@ki.se (Q.D.), elisabet.stener-victorin@ki.se (E.S.-V.)
https://doi.org/10.1016/j.xcrm.2023.101035

SUMMARY

The transgenerational maternal effects of polycystic ovary syndrome (PCOS) in female progeny are being revealed. As there is evidence that a male equivalent of PCOS may exists, we ask whether sons born to mothers with PCOS (PCOS-sons) transmit reproductive and metabolic phenotypes to their male progeny. Here, in a register-based cohort and a clinical case-control study, we find that PCOS-sons are more often obese and dyslipidemic. Our prenatal androgenized PCOS-like mouse model with or without diet-induced obesity confirmed that reproductive and metabolic dysfunctions in first-generation (F_1) male offspring are passed down to F_3. Sequencing of F_1–F_3 sperm reveals distinct differentially expressed (DE) small non-coding RNAs (sncRNAs) across generations in each lineage. Notably, common targets between transgenerational DEsncRNAs in mouse sperm and in PCOS-sons serum indicate similar effects of maternal hyperandrogenism, strengthening the translational relevance and highlighting a previously underappreciated risk of transmission of reproductive and metabolic dysfunction via the male germline.

INTRODUCTION

Polycystic ovary syndrome (PCOS) is the leading causes of female infertility and is associated with a high degree of comorbidities, including type 2 diabetes and psychiatric disorders.[1,2] The key feature of PCOS is hyperandrogenism,[3] and >50% of women with PCOS are obese,[4] which exacerbates their symptoms. Although ~15% of women worldwide suffer from PCOS, management of the syndrome is hindered by lack of insight into the origin and underlying mechanisms. It is known that PCOS runs in families with both genetic and epigenetic contributions, the latter of which are phenotypic changes that do not involve alterations in the DNA sequence,[5] and that daughters of women with PCOS are five times more likely to be diagnosed with the syndrome.[6] Although a distinct phenotype of male offspring related to PCOS has not yet been defined, sons born to mothers with PCOS (PCOS-sons) display increased body mass index (BMI), insulin resistance,[7,8] and prepubertal signs of reproductive dysfunction with increased antimüllerian (AMH) hormone levels, indicating increased Sertoli cell number.[9] However, at adult age, there were no differences in circulating AMH, sex steroids, or gonadotropins, in addition to sperm production.[9]

© 2023 The Author(s).
This is an open access article under the CC BY license (http://creativecommons.org/licenses/by/4.0/).

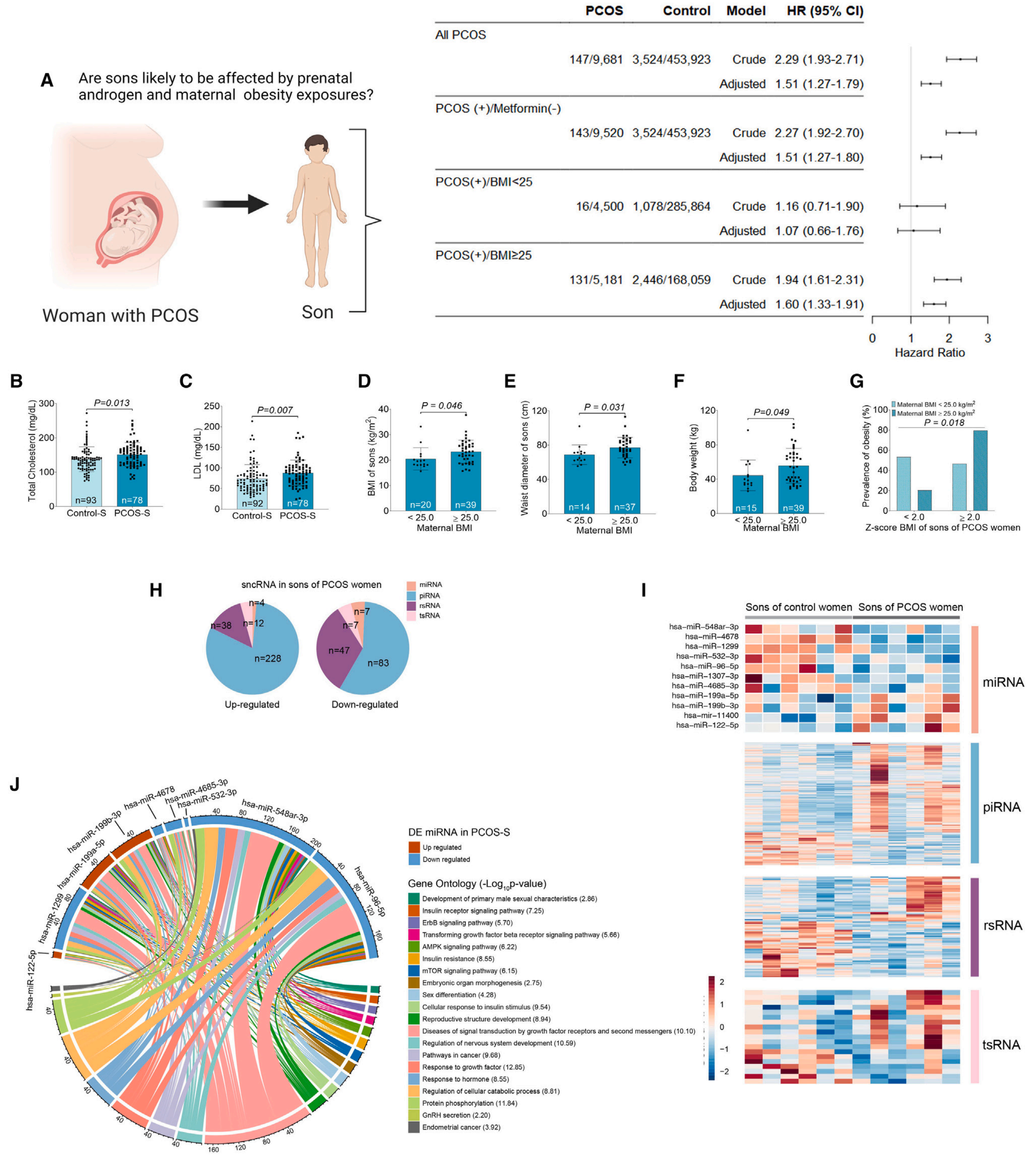

Figure 1. Risk of being obese and altered lipid profile of sons of women with PCOS

(A) Risk of obesity during childhood in sons born to mother with polycystic ovary syndrome (PCOS) identified in the Swedish National Patient Register and in the Multi-Generational Register (n = 467,275), expressed as hazard ratios (HRs) and 95% confidence intervals (CIs). The covariates in the adjusted model were maternal age at delivery stratified as <25, 25–29, 30–34, and ≥35; maternal BMI stratified as <18.5, 18.5–24.9, 25–29.9, and ≥30; parity (multiparous, nulliparous), cigarette consumption at enrollment (yes/no); assisted reproduction (yes/no); size for gestational age (adequate, small or large); preterm birth (yes/no); Apgar <7 at 5 min; cesarean section; diabetes (gestational diabetes, diabetes mellitus, or use of metformin during pregnancy); and finally sub-analyses of women with a BMI <25 and women with a BMI ≥25.

(legend continued on next page)

CellPress
OPEN ACCESS

Moreover, brothers of women with PCOS have increased AMH hormone levels, altered gonadotrophin, and steroidogenic secretion,[10,11] as well as a metabolic phenotype with insulin resistance and pancreatic β-cell dysfunction, dyslipidemia, and an increased cardiovascular disease risk.[12–16] Recently, it was shown that genetic risk factors for PCOS increase the odds of obesity, type 2 diabetes, cardiovascular disease, and androgenic alopecia in men.[17] Although genetic components are likely involved in a male-PCOS phenotype, the clinical observations suggest that maternal obesity and PCOS could also affect the development of male fetuses and predispose them to reproductive and metabolic disorders in later life, as shown in their female siblings.

Our and others' recent animal studies show that prenatal androgen or AMH exposure predisposes the first-generation (F_1) female offspring to develop PCOS-like traits, and both reproductive and metabolic phenotypic alterations are passed on to the F_3 of females, suggesting non-genetic transgenerational transmission.[6,18] Previous studies reveal that F_1 male offspring of both rodents and sheep develop an aberrant reproductive and metabolic phenotype due to prenatal androgen exposure.[19–23] Whether these phenotypic changes are transmitted further to subsequent male generations has not yet been explored. Other studies have demonstrated that maternal stress and endocrine disruptors in rodent models cause a transgenerational transmission of phenotypes on both female and male germline.[24–27] Diet-induced obesity from early life in male mice affects sperm with altered small non-coding RNAs (sncRNAs), which predisposes their male offspring in subsequent generations to obesity, suggesting epigenetic inheritance potentially driven by the germline.[28,29]

Our study now provides evidence that PCOS-sons have altered lipid profiles and are at higher risk to childhood obesity. Several differentially expressed (DE) miRNAs found in serum from PCOS-sons are overlapped with those identified in serum or follicular fluid of women with PCOS, likely to regulate PCOS-risk genes identified by genome-wide association studies (GWASs).[30–36] We then turned to the mouse models and showed that prenatal androgen exposure and/or maternal obesity resulted in the transmission of reproductive and metabolic traits to F_3 male offspring associated with common DEsncRNAs in sperm of F_1, F_2, and F_3 offspring (i.e., transgenerational DEsncRNAs). Moreover, we found that several DEsncRNAs in serum from PCOS-sons are shared with transgenerational DEsncRNAs in mouse sperm, highlighting the translational relevance of our transgenerational mouse studies.

RESULTS

PCOS-sons are more obese together with altered circulating lipid profile

Our previous findings show that PCOS-sons have increased BMI and abnormal glucose and lipid metabolism.[7,8] Besides, they have increased AMH levels during infancy, childhood, and adulthood as well as smaller testicular volume.[8] To follow up these findings in a large cohort, we performed a Swedish nationwide register-based cohort study to investigate whether PCOS-sons are more often diagnosed with obesity (Figure 1A; Table S1). Using the Swedish Medical Birth Register and the National Patient Register, a total of 467,275 sons born in Sweden between July 2006 and December 2015 were included and followed from 2 years of age. From them, 9,828 (2.10%) were born to a mother diagnosed with PCOS. Of the mothers diagnosed with PCOS, 165 (1.67%) had at least one prescription of metformin discharged from a pharmacy. Obesity diagnosed in children was identified by using the International Code of Diseases, v.10 (ICD-10: E66). Overall, an increased risk of obesity in sons born to mothers with PCOS (with or without use of metformin during pregnancy) was found (adjusted hazard ratio [HR] = 1.51 95% confidence interval [1.27–1.79]). A similar risk was found in the sub-analysis assessing only women with PCOS and without use of metformin. Finally, when stratifying maternal BMI, there was an association between maternal PCOS and childhood obesity only in the group of women with BMI ≥25 (HR = 1.60 [1.33–1.91]) with no association in women with BMI <25 (HR = 1.07 [0.66–1.76]) (Figure 1A). Of note, only 2.1% of the mothers had PCOS, which is much lower than the expected 10%–18% in the general population of reproductive age.[37] In a longitudinal case-control study from Chile,[7] we showed that already at Tanner II-III and Tanner IV-V PCOS-sons have higher circulating cholesterol and low-density lipoprotein (LDL) cholesterol compared with control sons (Figures 1B and 1C; Table S2). Moreover, sons of mothers with maternal obesity have higher BMI and larger waist circumference compared with sons of mothers with BMI <25 (Figures 1D–1F). The prevalence of children who were overweight and obese was higher in those children born to mothers with BMI >25 during pregnancy (Figure 1G).

sncRNA analysis on serum of PCOS-sons identifies miRNAs targeting loci of PCOS-risk genes

Next, we investigated molecular features in serum of PCOS-sons (Chilean case control) by sncRNA sequencing (sncRNA-seq). Among DEsncRNAs between sons of women with and without PCOS, Piwi-interacting RNAs (piRNAs), rRNA-derived small

(B and C) Total cholesterol (B) and low-density lipoprotein (C) in sons of women with PCOS at Tanner stages I–V.

(D–G) BMI of sons of women with PCOS (D), waist circumference of sons of women with PCOS (E), body weight of sons of women with PCOS (F), and prevalence of obesity of sons of women with PCOS (G) with *Z* score BMI <2 (normal weight) and *Z* score BMI ≥2 (overweight-obesity) according to maternal nutritional state at beginning of pregnancy distributed in BMI <25 and ≥25 kg/m^2. Differences were calculated by chi-squared test for prevalence values and Student's t test for BMI and waist diameter. Control-S, control son; PCOS-S, PCOS son.

(H) The numbers of differently expressed (DE) sncRNAs in each biotype in whole blood from sons of women with PCOS and control women (n = 9/group).

(I) Heatmap of DEsncRNA in whole blood from sons of women with PCOS and control women (n = 9/group).

(J) Chord diagram showing DEmiRNA in whole blood from sons of women with PCOS and their target genes. Chords in different colors represent Gene Ontology (GO) enrichment. The expression of the DEmiRNAs is shown in red (up-regulated) and blue (down-regulated).

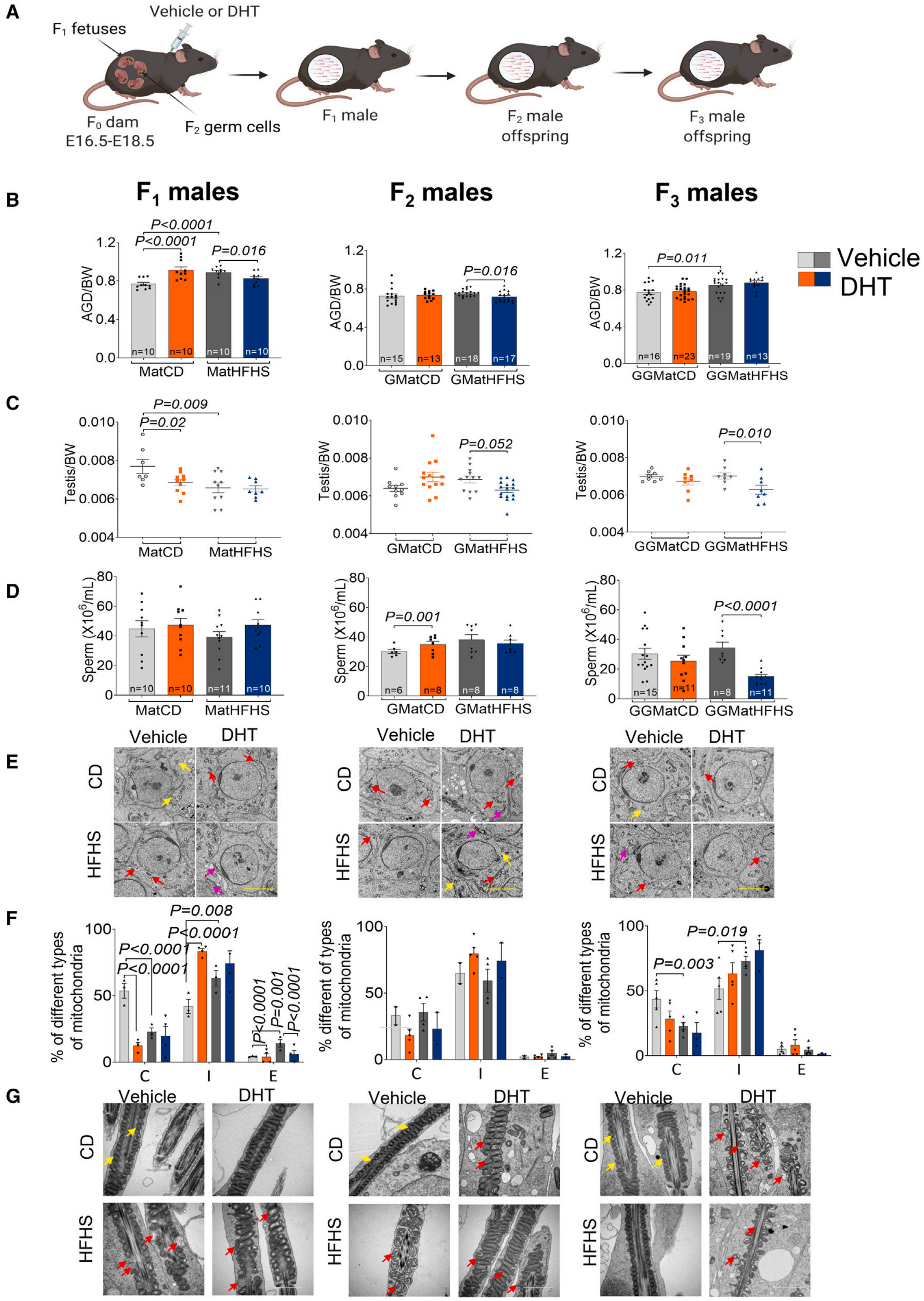

(legend on next page)

RNAs (rsRNAs), and microRNAs (miRNAs) were the major biotypes (Figures 1H and 1I). As circulating miRNAs are extensively characterized in gene regulation and as stable biomarkers, we first asked whether DEmiRNAs of serum of PCOS-sons are also identified in women with PCOS by comparing our data with previously profiled serum, granulosa cells, or follicular fluid miRNAs expression. We found that 7 out 11 DEmiRNAs in the serum of PCOS-sons were also DE in women with PCOS (Data S1): hsa-miR-1299,[38] hsa-miR-122-5p,[39] hsa-miR-199b-3p,[40,41] hsa-miR-199a-5p,[42] hsa-miR-1307-3p,[43] hsa-miR-96-5p,[44] and hsa-miR-548ar-3p.[41] Moreover, we examined *in silico* targets of these DEmiRNA and revealed 783 potential target genes (Data S2), among which six are reported as PCOS-risk genes by GWAS, i.e., *AOPEP*[30,33] (hsa-miR-1299), *TOX3*[32,45] (hsa-miR-1299), *ERBB4*[32] (hsa-miR-199b-3p), *GABRB1*[46] (hsa-miR-548ar-3p), *ADGRB3*[33] (hsa-miR-96-5p), and *MYRIP*[36] (hsa-miR-96-5p). To understand the function of these overlapped miRNAs, we performed Gene Ontology (GO) pathway analyses of targeted genes (Figure 1J; Data S3). Among the enriched pathways that potentially could contribute to the pathology of PCOS are insulin resistance (e.g., *FOXO1*, *MTOR*, *GYS1*, *PTEN*, *RPS6KB1*, *STAT3*, *CREB5*, *TRIB3*), sex differentiation (e.g., *FER*, *FOXF2*, *LRP2*, *PGR*, *SIRT1*, *LHX9*, *AGO4*), response to hormone (e.g., *KLF9*, *GABRB1*, *ITGA3*, *BCAR3*, *CYP7B1*), regulation of cellular catabolic process (e.g., *ABCA2*, *ABCD1*, *PIK3CA*, *DISC1*, *MAP3K5*), and GnRH secretion (e.g., *ITPR1*, *PIK3CA*, *PIK3R1*, *PLCB4*, *PIK3R3*).

Maternal obesity in F_0 dam causes transgenerational reproductive dysfunction in male offspring

We used our previously validated mouse models[6] to investigate whether F_1 male offspring that were directly exposed to diet-induced maternal obesity, prenatal androgens, or the combination of the two exposures could develop reproductive traits in adult males and if such traits are passed on to F_2 (direct germline exposure, i.e., intergenerational) and F_3 (transgenerational) male offspring. The phenotype of F_0 dams has recently been described in detail.[6] In total, four experimental lineages were studied: (1) control diet + vehicle (control); (2) control diet + dihydrotestosterone (androgenized); (3) high-fat, high-sucrose diet + vehicle (obese); and (4) high-fat, high-sucrose diet + dihydrotestosterone (obese and androgenized) (Figure 2A). F_1 male offspring were mated with unrelated healthy females to generate F_2, and F_2 male offspring were mated with unrelated healthy females to generate F_3 and each generation were compared with parallel bred controls, which is required to study transgenerational inheritance. Phenotypic testing was performed between 15 and 22 weeks of age in each generation.

Anogenital distance, a marker of *in utero* androgen exposure,[47] was longer in F_1 and F_3 male offspring in the obese lineage, demonstrating a transgenerational effect due to maternal obesity, whereas it was longer only in the F_1 male offspring in the androgenized lineage (Figure 2B). Notably, the transgenerational transmission of anogenital distance was independent of circulating sex steroids, as we found neither differences in circulating testosterone, dihydrotestosterone, and androstenedione nor in testis AMH concentrations in F_1 and F_3 male offspring in any of the lineages (Figures S1A–S1D), suggesting that the transgenerational effects are caused by initial maternal condition rather than excessive circulating androgens in F_1 and F_3 male offspring.

Moreover, we found lower testis weight in F_1 male offspring in the androgenized and obese lineages, respectively, compared with controls, although no difference was observed in their respective F_3 males (Figure 2C). In contrast, there was a latent effect in F_2 and F_3 male offspring in the combined obese and androgenized lineage with lower testis weight compared with the obese lineage (Figure 2C). In line with the low testis weight, the obese and androgenized lineage of F_3 showed a low total sperm count (Figure 2D).

Maternal obesity and prenatal androgen exposure affect mitochondrial morphology of MII oocytes.[6] Accordingly, we analyzed

Figure 2. Prenatal androgen and maternal obesity exposure causes transgenerational reproductive phenotypes in male offspring

(A) Schematic illustration of the experimental design. (1) CD+Veh (control lineage), (2) CD+DHT (androgenized lineage), (3) HFHS+Veh (obese lineage), and (4) HFHS+DHT (obese and androgenized lineage).

(B) Transgenerational increase in anogenital distance (AGD) in the obese and the obese and androgenized lineages. CD+Veh (F_1: n = 10, litters = 5; F_2: n = 15, litters = 4; F_3: n = 16, litters = 4); CD+DHT (F_1: n = 10, litters = 5; F_2: n = 13, litters = 4; F_3: n = 23, litters = 4); HFHS+Veh (F_1: n = 10, litters = 5; F_2: n = 18, litters = 4; F_3: n = 19, litters = 4); and HFHS+DHT (F_1: n = 10, litters = 7; F_2: n = 17, litters = 4; F_3: n = 13, litters = 4).

(C) Testis weight normalized to body weight in F_1–F_3 male offspring. CD+Veh (F_1: n = 7, litters = 5; F_2: n = 10, litters = 4; F_3: n = 8, litters = 4); CD+DHT (F_1: n = 10, litters = 5; F_2: n = 13, litters = 4; F_3: n = 8, litters = 4); HFHS+Veh (F_1: n = 10, litters = 5; F_2: n = 12, litters = 4; F_3: n = 8, litters = 4); and HFHS+DHT (F_1: n = 8, litters = 7; F_2: n = 16, litters = 4; F_3: n = 8, litters = 4).

(D) Total sperm counts in F_1–F_3 male offspring. CD+Veh (F_1: n = 10, litters = 5; F_2: n = 6, litters = 4; F_3: n = 16, litters = 4); CD+DHT (F_1: n = 10, litters = 5; F_2: n = 8, litters = 4; F_3: n = 12, litters = 4); HFHS+Veh (F_1: n = 11, litters = 5; F_2: n = 8, litters = 4; F_3: n = 6, litters = 4); and HFHS+DHT (F_1: n = 10, litters = 7; F_2: n = 8, litters = 4; F_3: n = 11, litters = 4).

(E) Representative transmission electronic microscopy images of mitochondrial morphology in pachytene spermatocytes in F_1–F_3 testis. Yellow arrows: condense mitochondria, red arrows: intermediate form of mitochondria, and pink arrows: elongated form of mitochondria.

(F) Quantification of mitochondrial morphology: normal type, condense (C); abnormal types, intermediate (I) and elongated (E) forms, in pachytene spermatocytes in F_1, F_2, and F_3 male offspring. Scale bar: 5 μm. CD+Veh (F_1: n = 3, litters = 3; F_2: n = 2, litters = 2; F_3: n = 5, litters = 4); CD+DHT (F_1: n = 3, litters = 3; F_2: n = 5, litters = 4; F_3: n = 5, litters = 4); HFHS+Veh (F_1: n = 3, litters = 3; F_2: n = 4, litters = 4; F_3: n = 5, litters = 4); and HFHS+DHT (F_1: n = 3, litters = 3; F_2: n = 2, litters = 2; F_3: n = 3, litters = 3).

(G) Representative images of the mitochondrial sheath of sperm from testis in F_1, F_2, and F_3 male offspring. Yellow arrows: normal mitochondria, and red arrows: abnormal mitochondria. Scale bar: 5 μm.

Lineage: control: CD+Veh, maternal control diet + vehicle exposure; androgenized: CD+DHT, maternal control diet + dihydrotestosterone exposure; obese: HFHS+Veh, maternal high-fat, high-sucrose diet + vehicle exposure; and the obese and androgenized: HFHS+DHT, maternal high-fat, high-sucrose diet + dihydrotestosterone exposure. Comparison between the groups was performed using linear mixed-effects models and non-repeated measures (ANODE, R package car). Each dot represents one offspring mouse. All data are presented as mean ± SEM.

mitochondrial morphology in the testis by transmission electron microscopy. During spermatogenesis, three different types of cristae morphology are present in mitochondria: orthodox type (Sertoli cells, spermatogonia, and preleptotene and leptotene spermatocytes), intermediate type (zygotene spermatocytes), and condense type (pachytene and secondary spermatocytes and early spermatids).[48] F_1 of the androgenized lineage and F_1 and F_3 male offspring of the obese lineage had spermatocytes with more intermediate-type (abnormal) mitochondria and a declining number of condensed (normal) mitochondria in pachytene spermatocytes and round spermatids (Figures 2E and 2F). We further analyzed the mitochondrial sheath of sperm and found abnormal crista structures with vacuoles, indicating morphological abnormalities in F_1 male offspring in the obese and the obese and androgenized lineages (Figure 2G). In F_2 male offspring, the aberrant mitochondrial sheath was observed in all three lineages (Figure 2G), which was retained in F_3 male offspring as a transgenerational effect (Figure 2G). These findings were further supported by dysregulated expression of key mitochondrial genes in testis of F_1, F_2, and F_3 male offspring, namely *Tfam1* (transcription factor A; mitochondrial), *Drp1* (dynamin-related protein 1), *Opa1* (OPA1 mitochondrial dynamin-like GTPase), and *Guf1* (GUF1 homolog; GTPase) (Figure S1E). Despite these mitochondrial phenotypes, there are no significant changes in sperm morphology in F_1–F_3 male offspring and no effect on the fecundity of F_1 and F_2 adult males (Figures S1F–S1H).

Collectively, these results suggest that altered reproductive (testis and sperm mitochondrial) functions are transmitted across generations in male offspring of the obese lineage, and there is a strong and belated effect in F_3 male progeny in the combined obese and androgenized lineage.

Prenatal androgen exposure and maternal obesity cause transgenerational metabolic dysfunction in male offspring

F_1 male offspring in the androgenized and in the androgenized and obese lineages, respectively, gained more weight, whereas F_2 in the androgenized lineage gained less weight with no difference in F_3 male offspring (Figure S1I). Both F_1 and F_3 male offspring in the androgenized and in the obese lineages, respectively, had more fat mass (Figure 3A). Increased fat mass was observed independent of lean mass (Figure S1J). In support of the increased adiposity in F_1 and F_3 male offspring in the androgenized and obese lineages, we also found enlarged epididymal adipocytes (Figures 3B and 3C). The observation of increased fat mass and enlarged adipocytes was further supported by impaired glucose metabolism in F_1 and F_3 male offspring in the obese lineage as shown by increased area under the curve-oral glucose tolerance test (AUC-OGTT) (Figures 3D, 3E, and S2A).

Although there was no transgenerational transmission of adiposity and impaired glucose homeostasis in the combined obese and androgenized lineage, we found higher liver triglycerides content in F_3 male offspring (Figure 3F). Furthermore, the accumulation of neutral lipids in the liver further corroborated these observations in the combined lineage, as well as the transgenerational effects of increased fat mass and enlarged epididymal adipocytes in the obese and androgenized lineages, respectively (Figure 3G).

To gain a deeper understanding of the metabolic phenotypes, we used indirect calorimetry and found that F_1 male offspring in the androgenized and the combined lineages, and F_3 male offspring in all lineages, had altered respiratory exchange ratio (RER) (day or night), which is an indicator of fuel selection and -utilization and altered energy expenditure (EE), indicating dysfunctional energy metabolism (Figures 3H, 3I, and S2B–S2D). These results demonstrate a transition from carbohydrate to fatty acid consumption, which corresponded to increased adiposity in F_1 and F_3 male offspring and occurred without a change in food intake and total activity.

These findings suggest that metabolic dysfunction in F_1 male offspring as an effect of maternal obesity or prenatal androgen exposure are transmitted across generations, whereas the transgenerational effect in the combined obese and androgenized lineage is less pronounced.

Reproductive and metabolic function in F_2 male offspring is less affected. The phenomenon that phenotypic changes skip one (or even two) generations has previously been observed in the female and male germline by us and others.[6,49,50]

sncRNAs in sperm accompany transgenerational transmission of phenotypic traits

To dissect the molecular basis of transmission of PCOS-related dysfunction, sperm was collected from the cauda epididymis and subjected to a swim-up assay to ensure that only motile mature spermatozoa, i.e., the pure fraction of ~10 million sperm was used for sncRNA-seq analysis of F_1, F_2, and F_3 male offspring (Figure 4A). First, we found that sperm from different lineages in F_1, F_2, and F_3 male offspring show various sncRNA biotypes: miRNA (22–24 nt), piRNA (20–34 nt), rsRNAs (15–44 nt), and tRNA-derived small RNAs (tsRNA; 27–36 nt) (Figure S3A). To reveal the molecular basis of phenotypic variation, we further analyzed DEsncRNAs in F_1, F_2, and F_3 offspring of the androgenized, the obese, and the obese and androgen lineages (Figure S3B).

First, we performed principal-component analysis (PCA) on the sncRNA profiles for the three comparisons: (1) control (control diet [CD] + vehicle [Veh]) vs. androgenized lineages (CD + dihydrotestosterone [DHT]), (2) CD+Veh vs. obese lineages (high fat, high sucrose [HFHS] + Veh), and (3) HFHS+Veh vs. obese and androgenized lineages (HFHS+DHT), respectively. The obese lineages, with or without androgen exposure, show a clear separation of F_1–F_3 sncRNA profiles from controls, with less prominent separation in the androgenized lineage (Figure 4B). Next, we defined transgenerational up-regulated and down-regulated DEsncRNAs of F_1, F_2, and F_3 male offspring sperm and identified 8, 140, and 9 transgenerational DEsncRNAs in the androgenized, obese, and combined obese and androgenized lineages, respectively (Figure 4C; Data S4). Interestingly, the obese lineage has the greatest number and diverse biotypes of DEsncRNAs across the three generations, indicating that maternal obesity resulted in more transgenerational effectors compared with prenatal androgen exposure. This interpretation agreed with phenotypic data that the obese lineage showed clear aspects of transgenerational reproductive and metabolic dysfunctions, whereas the androgenized lineage only shows transgenerational metabolic dysfunctions. This may also explain

CellPress
OPEN ACCESS

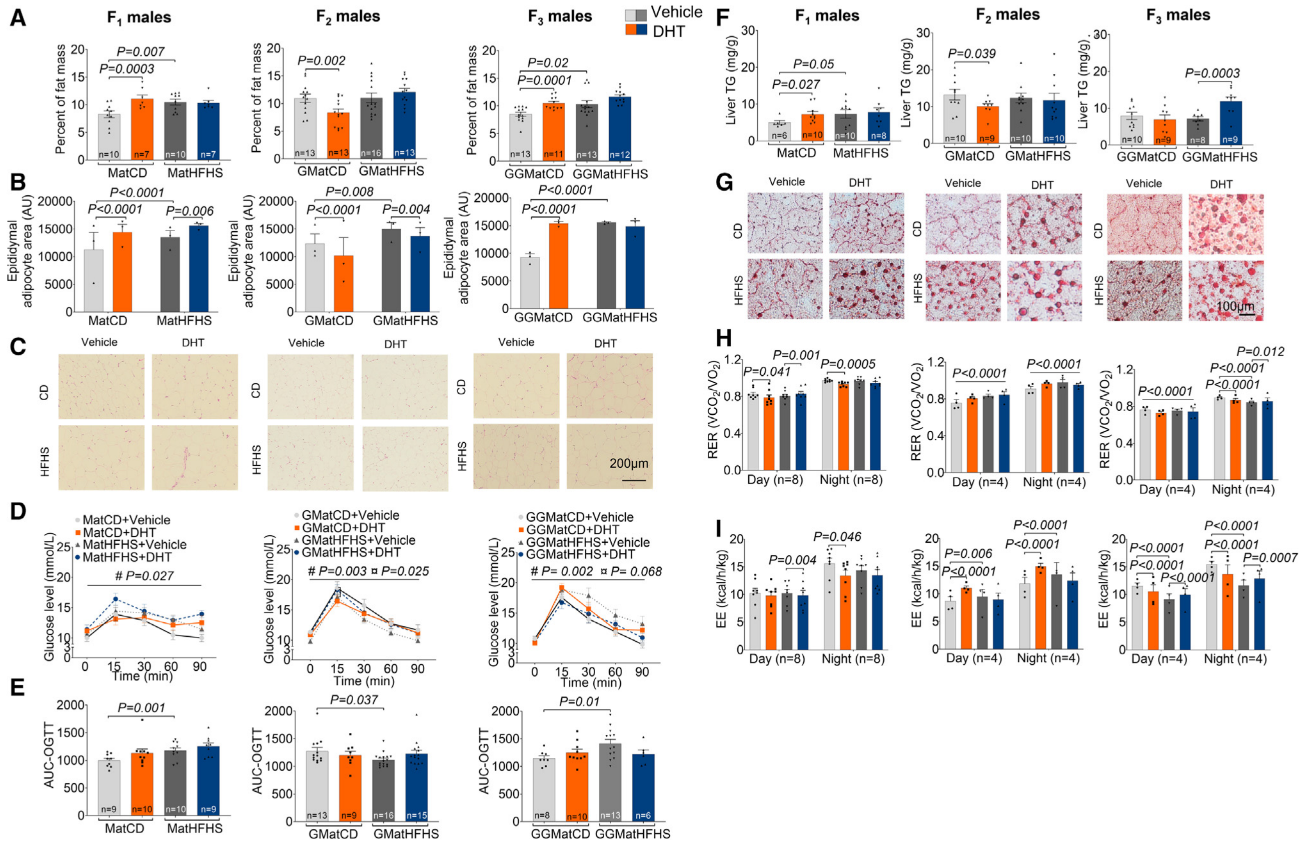

Figure 3. Prenatal androgen and maternal obesity exposure causes transgenerational metabolic dysfunction in male offspring and reproductive dysfunction in male cousins caused by androgen exposure

(A) Body composition presented as percentage of fat mass normalized to body weight (grams). CD+Veh (F_1: n = 10, litters = 5; F_2: n = 13, litters = 4; F_3: n = 13, litters = 4); CD+DHT (F_1: n = 7, litters = 5; F_2: n = 13, litters = 4; F_3: n = 11, litters = 4); HFHS+Veh (F_1: n = 10, litters = 5; F_2: n = 16, litters = 4; F_3: n = 13, litters = 4); and HFHS+DHT (F_1: n = 7, litters = 4; F_2: n = 13, litters = 4; F_3: n = 12, litters = 4).

(B) Epididymal adipocyte size measurements were made on six sections per mouse of F_1–F_3. CD+Veh (F_1: n = 3 litters = 3; F_2: n = 3, litters = 4; F_3: n = 3, litters = 3); CD+DHT (F_1: n = 3 litters = 3; F_2: n = 3, litters = 4; F_3: n = 3, litters = 3); HFHS+Veh (F_1: n = 3 litters = 3; F_2: n = 3, litters = 4; F_3: n = 3, litters = 3); and HFHS+DHT (F_1: n = 3 litters = 3; F_2: n = 3, litters = 4; F_3: n = 3, litters = 3).

(C) Representative images of epididymal adipocytes stained with hematoxylin and eosin. Scale bar: 200 μm.

(D) Blood glucose levels at different time points during oral glucose tolerance test (OGTT).

(E) Glucose area under the curve (AUC) at 0 to 90 min in F_1–F_3 adult male offspring. CD+Veh (F_1: n = 9 litters = 5; F_2: n = 13, litters = 4; F_3: n = 8, litters = 4); CD+DHT (F_1: n = 10, litters = 5; F_2: n = 9, litters = 4; F_3: n = 10, litters = 4); HFHS+Veh (F_1: n = 10, litters = 5; F_2: n = 16, litters = 4; F_3: n = 13, litters = 4); and HFHS+DHT (F_1: n = 9, litters = 6; F_2: n = 15, litters = 4; F_3: n = 6, litters = 4).

(F) Liver triglyceride (TG) content in F_1–F_3 male offspring normalized to tissue weight (mg/g). CD+Veh (F_1: n = 6 litters = 5; F_2: n = 10, litters = 4; F_3: n = 10, litters = 4); CD+DHT (F_1: n = 10, litters = 5; F_2: n = 9, litters = 4; F_3: n = 9, litters = 4); HFHS+Veh (F_1: n = 10, litters = 5; F_2: n = 10, litters = 4; F_3: n = 8, litters = 4); and HFHS+DHT (F_1: n = 8, litters = 6; F_2: n = 10, litters = 4; F_3: n = 9, litters = 4).

(G) Representative images of neutral lipid accumulation in the liver visualized by oil red O staining. Scale bar: 100 μm.

(H and I) Respiratory exchange ratio (RER; VCO_2/VO_2) (H) and energy expenditure (EE) (I) was measured by indirect calorimetry by using the TSE system in F_1–F_3 adult male offspring. CD+Veh (F_1: n = 8 litters = 4; F_2: n = 4, litters = 4; F_3: n = 4, litters = 4); CD+DHT (F_1: n = 8, litters = 4; F_2: n = 4, litters = 4; F_3: n = 4, litters = 4); HFHS+Veh (F_1: n = 8, litters = 4; F_2: n = 4, litters = 4; F_3: n = 4, litters = 4); and HFHS+DHT (F_1: n = 8, litters = 5; F_2: n = 4, litters = 4; F_3: n = 4, litters = 4).

Lineage: CD+Veh, maternal control diet + vehicle exposure; CD+DHT, maternal control diet + dihydrotestosterone exposure; HFHS+Veh, maternal high-fat, high-sucrose diet + vehicle exposure. Comparison between the groups were performed using linear mixed-effects models and non-repeated measures (ANODE, R package car) except for (D), where linear mixed-effects models with repeated measure was used. #, CD+Veh vs. HFHS+Veh; ¤, HFHS+Veh vs. HFHS+DHT. Each dot represents one offspring mouse. All data are presented as mean ± SEM.

why there is a belated transgenerational effect in the combined obese and androgenized lineage.

Next, we investigated where the transgenerational DEsncRNAs are derived from.[51] Distinct from prepachytene piRNAs mainly silencing retrotransposons to protect the integrity of the genome,[52,53] pachytene piRNAs are dominantly transcribed from genic and intergenic regions and instruct mRNA degradation during late spermrmatogenesis[54] Accordingly, the majority of identified transgenerational DEpiRNAs are presented within intergenic and genic regions in the obese lineage (Figure 5A). However, only two transgenerational DEpiRNA in the androgenized (in the genic and intergenic region) and one

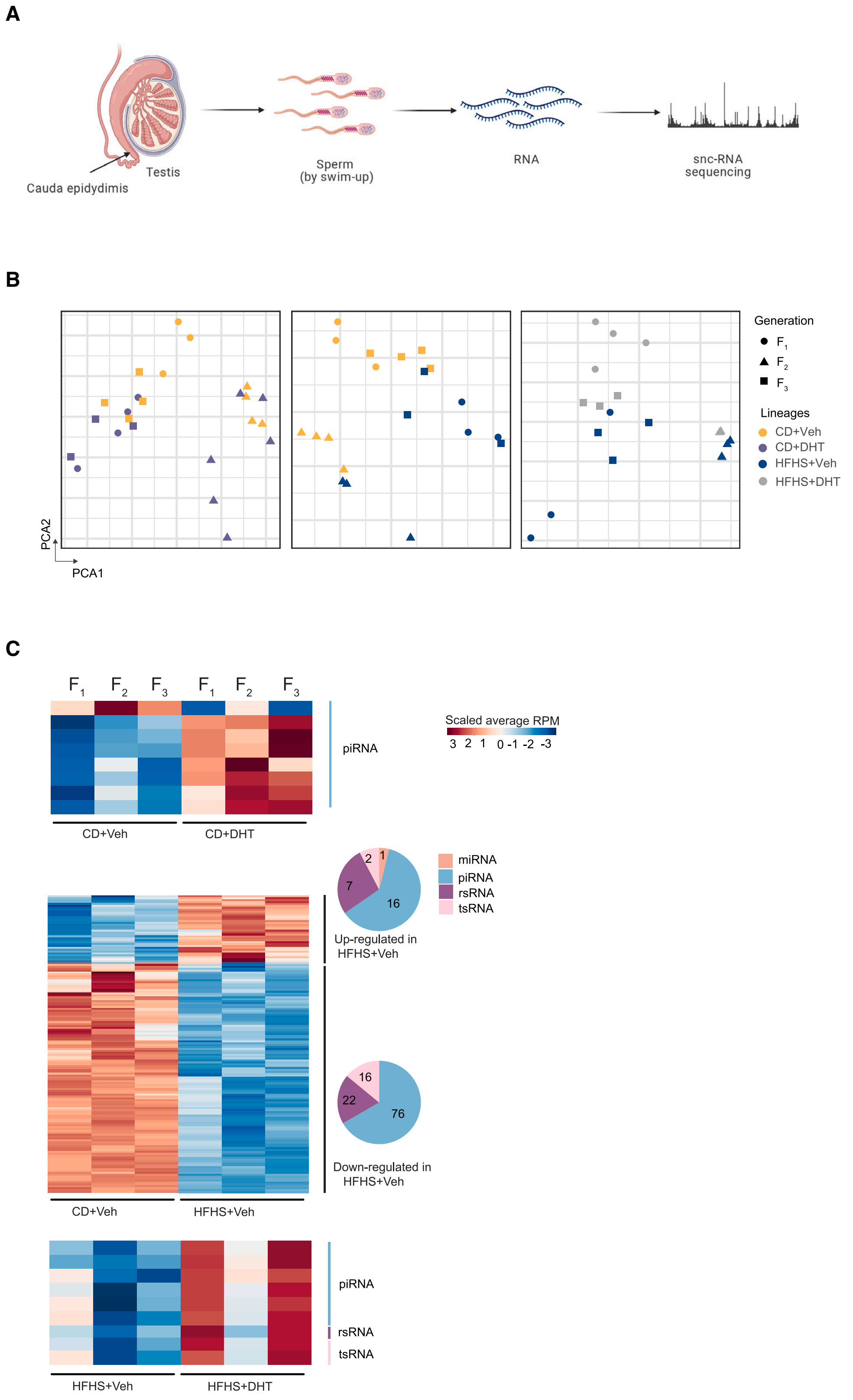

(legend on next page)

transgenerational DEpiRNA in the combined obese and androgenized lineages (in the repeat region) were annotated, respectively. rsRNAs were classified according to their origins as mitochondrial 12S and 16S rRNAs; ribosomal 18S, 28S, 45S, 4.5S, and 5.8S rRNAs; and nuclear 5S rRNAs. The transgenerational rsRNAs in the obese lineage derives mainly from 28S, 18S, and 45S rRNA (Figure 5B). In the obese and androgenized lineage, only one transgenerational DErsRNA was derived from 28S rRNA, with no DErsRNAs in the androgenized lineage. tsRNAs were systematically annotated from tRNAs according to their coupled amino acids from genome and mitochondria. The majority of the transgenerational DEtsRNAs in the obese lineage are derived from Glu and Gly tRNA, followed by Val, Gln, and mitochondrial tRNAs (Figure 5C). One tRNA can give rise to various tsRNAs, whose origins are mainly categorized into 5′-tRNA, 3′-tRNA, internal-tRNA, and 3′CCA-tRNA. Transgenerational tsRNAs in the obese lineage were mainly derived from 5′-tRNA and internal tRNA, with a small proportion derived from 3′-tRNA with or without CCA end (Figure 5C).

A family of X-linked miRNAs predominantly express in mammalian sperms and were named spermatogenesis-related miRNAs (spermiRs).[55] We next investigated whether the spermiRs were transgenerationally dysregulated (Data S5). In the androgenized and the obese lineages, respectively, five X-linked spermiRs (mmu-miR-465a-5p, mmu-miR-743b-3p, mmu-miR-470-5p, mmu-miR-871-3p, mmu-miR-741-3p) were up-regulated in F_2 male offspring, and four of these miRNAs (except mmu-miR-470-5p) were DE in the obese lineage in F_3 generation.[55]

Thereafter we annotated the function of transgenerational DEmiRNA target genes in each lineage and each generation using pathway enrichment analyses (Data S6A). When overlapping enriched biological pathways across the three generations, we found different pathways enriched in each respective lineage. In the androgenized lineage, enriched biological processes are implicated in morphogenesis of epithelium (e.g., *Edn1*, *Ajuba*, *Cited2*, *Nrp1*, *Frs2* [mmu-miR-1a-3p]), and in the combined obese and androgenized lineage, the three pathways enriched are related to protein phosphorylation (e.g., *Epha7* [mmu-miR-130b-5p], *Ptk2b*, *Rock2*, *Itch* [mmu-miR-6240]) (Figure 5D; Data S6B). In the obese lineage, the biological processes are mainly enriched *in utero* embryonic development (e.g., *Socs3*, *Sox6*, *Zbtb18* (mmu-miR-19a-3p), *Fgfr1*, *Hif1a* [mmu-miR-6240]); vasculature development (e.g., *Bmpr2* [mmu-miR-467d-3p], *Egr3* [mmu-miR-23b-3p], *Lrp2* [mmu-miR-142a-5p, mmu-miR-148a-3p, mmu-miR-152-3p, mmu-miR-199a-3p, and mmu-miR-199b-3p], *Adipor2* [mmu-miR-19a-3p, mmu-miR-19b-3p, and mmu-miR-218-5p], *Slc4a7*, *Esr1* [miR-19a-3p and mmu-miR-148b-3p], *Tsc1* [mmu-miR-130a-3p, miR-19a-3p, and miR-19b-3p]); and neuron-related pathways (e.g., *Dlx1* [mmu-miR-19a-3p and mmu-miR-19b-3p], *Id2* [mmu-miR-19a-3p], *Myo5b* [mmu-miR-6240], *Atxn1* [mmu-miR-101a-3p, mmu-miR-125a-5p, and mmu-miR-141-3p]) (Figure 5E; Data S6B). Collectively, these data show that DEsncRNAs carried by sperm are correlated with the transgenerational transmission of metabolic and reproductive phenotype in male offspring.

Comparison between DEsncRNAs blood of PCOS-sons and mouse sperm

Next, we aligned the DEsncRNAs identified in sons of women with and without PCOS and each mouse lineage (Figure 6A) and selected those with >90% sequence homology to investigate if the human DEsncRNAs overlap with the identified transgenerational DEsncRNAs in mice. Overall, the obese lineage transgenerational DEsncRNAs has the greatest number of overlaps with DEsncRNAs from sons of women with PCOS (Figure 6B; Data S7). The transgenerational DEtsRNA in the androgenized and the obese lineages, respectively, overlap with the human DEtsRNA specifically derived from the 5′ end of tRNA-Val-CAC (Figure 6C; Data S7). Finally, we also identified several shared biological processes of DEmiRNA target genes in serum of PCOS-sons and sperm of male F1 offspring in the different lineages such as male gonad development, primary male sexual characteristic development, embryo organ development in the androgenized lineage, etc. (Figure 6D; Data S8). Common biological processes of DEmiRNA target genes in serum of PCOS-sons and sperm of male F1 offspring in the different lineages are vasculature development, male gonad development, primary male sexual characteristics development, and embryo organ development in the androgenized lineage; *in utero* embryonic development, neuron development, cell differentiation, and cell catabolic process in the obese linage; and protein phosphorylation, nervous system development, cell activity, and vasculature development pathways in the combined linage.

Taken together, these common DEsncRNAs found in PCOS-sons and offspring of androgenized and obese lineages suggest their roles in regulating PCOS-like phenotypic traits.

DISCUSSION

As to other complex diseases such as type 2 diabetes,[56,57] PCOS is a highly heritable disorder.[58] In addition to genetic factors, growing evidence from clinical and preclinical studies suggests that epigenetic regulation triggered by an adverse maternal-fetal environment could result in phenotypic transmission similar to conventional genetic effects as demonstrated by us and others.[6,18] More intriguingly, a recent study showed that men who carry high polygenic risk scores for PCOS develop an increased risk of obesity, type 2 diabetes, and cardiovascular

Figure 4. The transgenerational inheritance pattern of sncRNA in sperm of androgenized, obese, and obese and androgenized lineages
(A) Illustration showing the collection of sperm for sncRNA sequencing.
(B) The principal-component analysis (PCA) showing different lineages in F_1, F_2, and F_3 male offspring.
(C) Transgenerational expression patterns of F_1, F_2, and F_3 sperm differentially expressed miRNA, piRNA, rsRNA, and tsRNA.
The heatmaps show the $\log_2$ fold change (FC) of overlapped differentially expressed sncRNAs in F_1, F_2, and F_3. CD+Veh (F_1: n = 4, litters = 4; F_2: n = 4, litters = 4; F_3: n = 4, litters = 4); CD+DHT (F_1: n = 3, litters = 3; F_2: n = 4, litters = 4; F_3: n = 3, litters = 3); HFHS+Veh (F_1: n = 4, litters = 4; F_2: n = 4, litters = 4; F_3: n = 4, litters = 4); and HFHS+DHT (F_1: n = 4, litters = 4; F_2: n = 4, litters = 4; F_3: n = 4, litters = 4).

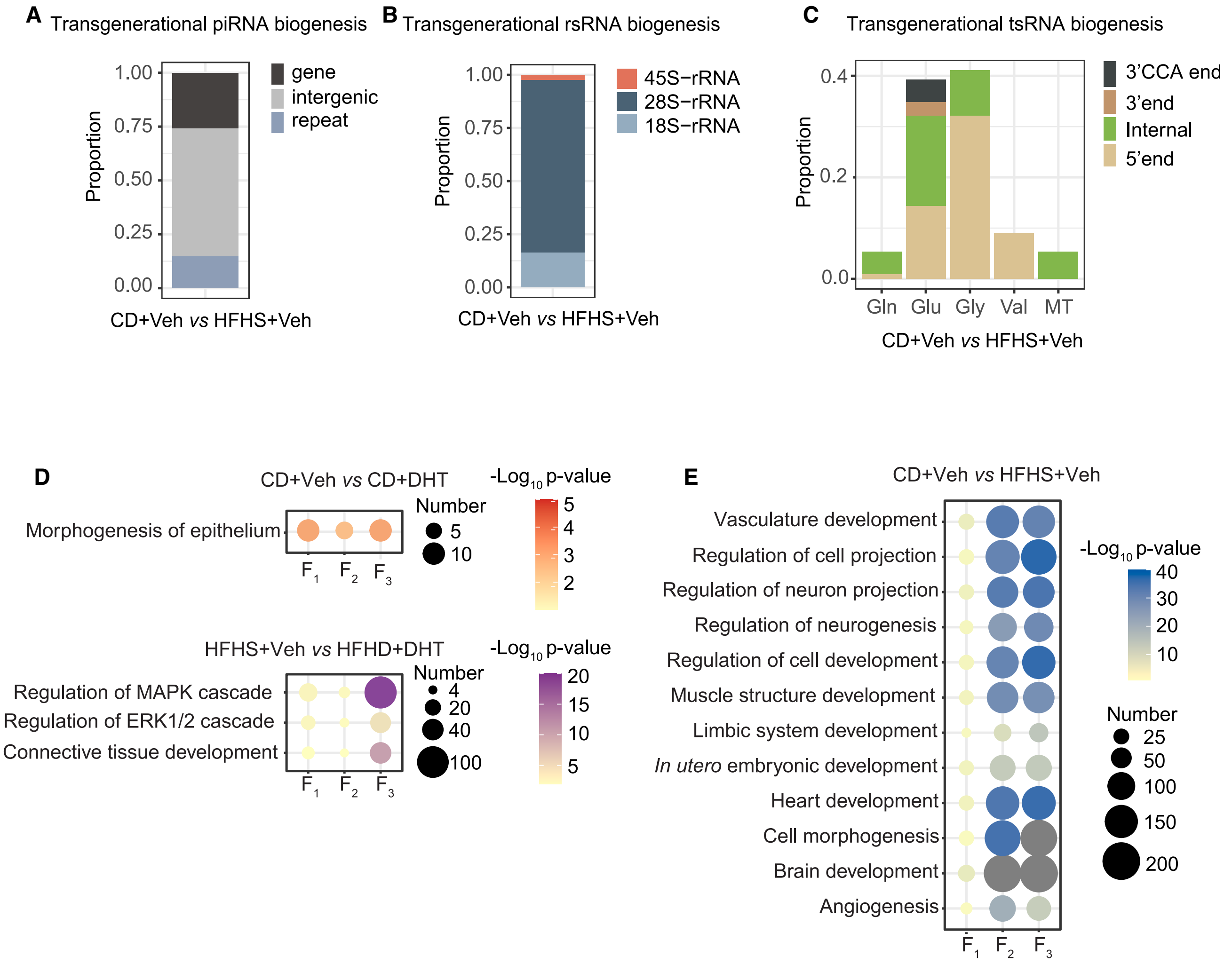

Figure 5. Transgenerational differential expression of sperm piRNA, rsRNAs and tsRNAs

(A–C) Proportion of transgenerational (in sperm of F_1, F_2, and F_3) biogenesis of (A) piRNA, (B) rsRNA, and (C) tsRNA in obese lineage.

(D) The overlapped GO enrichment for DEmiRNA target genes in F_1–F_3 transgenerational androgenized and obese and androgenized lineages.

(E) The overlapped GO enrichment for DEmiRNA target genes in F_1–F_3 transgenerational obese lineage.

Representative pathways with $\log_{10}$ (p value) >2 are presented. Size of the bubble represents the number of genes enriched in the pathway, and the gray color means the number of genes is larger than 200. Color represents $-\log_{10}$ (p value). CD+Veh (F_1: n = 4, litters = 4; F_2: n = 4, litters = 4; F_3: n = 4, litters = 4); CD+DHT (F_1: n = 3, litters = 3; F_2: n = 4, litters = 4; F_3: n = 3, litters = 3); HFHS+Veh (F_1: n = 4, litters = 4; F_2: n = 4, litters = 4; F_3: n = 4, litters = 4); and HFHS+DHT (F_1: n = 4, litters = 4; F_2: n = 4, litters = 4; F_3: n = 4, litters = 4).

disease, as well as male-pattern baldness, indicating that PCOS-sons could also be adversely affected.[17]

To identify common features in PCOS-sons, we here explored a large Swedish register-based cohort study and a Chilean case-control study and found that PCOS-sons are more likely to be diagnosed with obesity and display an altered lipid profile with high circulating total cholesterol and LDL cholesterol. In the Chilean cohort, we did observe significantly increased body weight and lipid profile alterations likely reflecting insulin resistance that is translated into obesity during adult life as we have previously reported.[8] These findings demonstrate that sons of obese women are more metabolically affected and highlight the importance of weight counseling and preferably weight reduction prior to pregnancy, especially in women with PCOS. Testicular volumes were comparable due to the initial study design for recruiting the pubertal group; nevertheless, previous work by our group showed that testicular volumes are lower at adulthood in PCOS-sons.[9] Moreover, our serum sncRNA analyses of sons from women with and without PCOS in the longitudinal Chilean clinical cohort study allowed us, for the first time, to identify potential miRNAs that underlie phenotypic transmission in humans by performing comparisons with published miRNA profiles from women with or without PCOS.[38,59–66] Several overlapped miRNAs are enriched in pathways that could contribute to the metabolic and reproductive phenotypic features observed in PCOS-sons including insulin resistance (hsa-miR-122-5p, hsa-miR-4678), sex differentiation (hsa-miR-199a-5p, hsa-miR-532-3p, hsa-miR-548ar-3p), and response to hormones (hsa-miR-122-5p,

CellPress
OPEN ACCESS

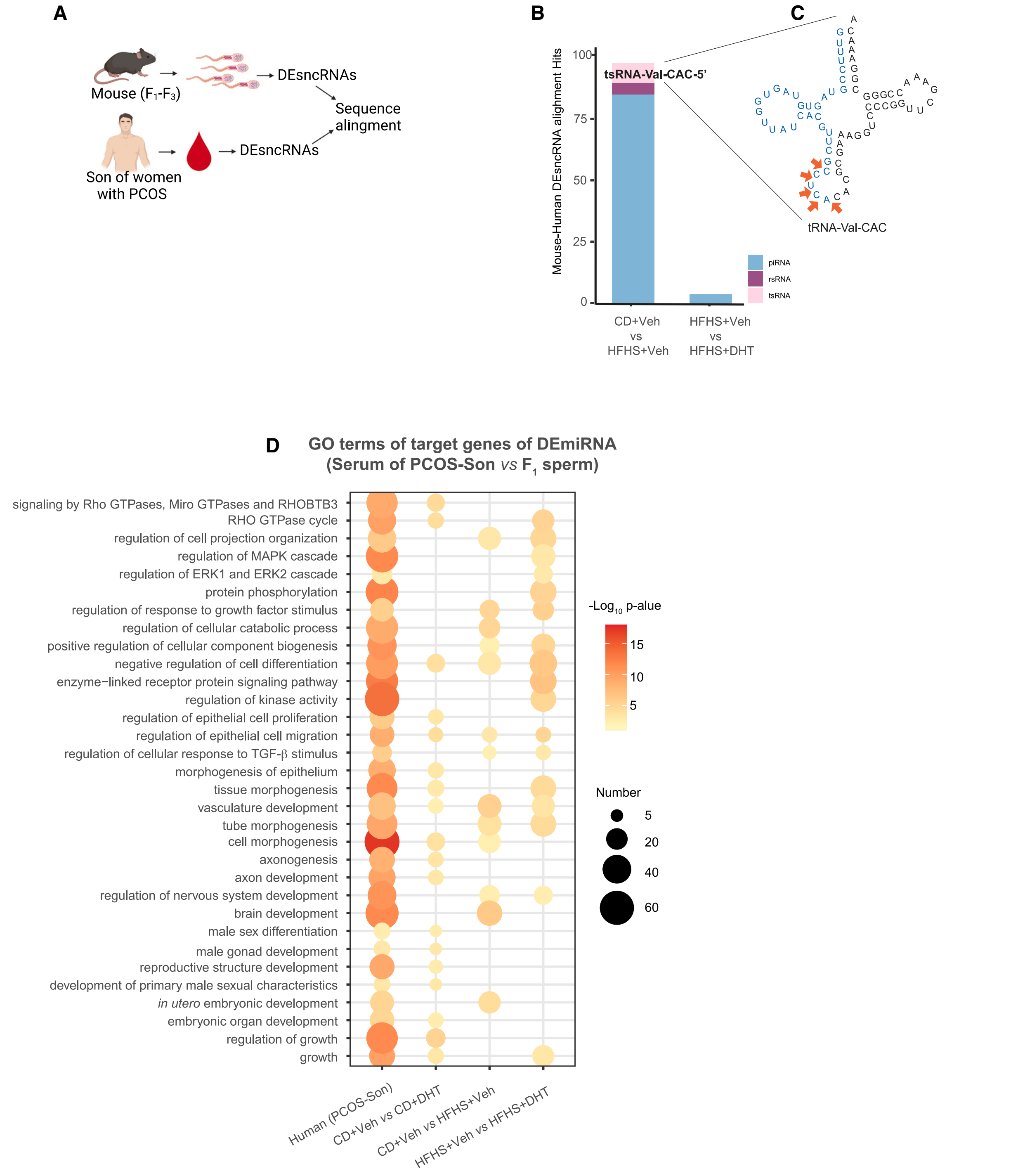

Figure 6. sncRNA analysis in whole blood of sons from mothers with PCOS and comparison with sperm of mice

(A) Schematic illustration of workflow showing how we identify human and mouse homologous DEsncRNA.

(B) Number and biotype of homologous pairs of human and mouse transgenerational DEsncRNAs in each lineage.

(C) Cloverleaf structure of tRNA-Val-CAC showing the region (blue) where the human and mouse common DEtsRNAs (as in B) are derived, with arrows pointing to the cutting sites.

(D) GO enrichment for target genes of overlapped DEsncRNAs between mouse sperm (F_1) and human blood.

(legend continued on next page)

hsa-miR-199a-5p, hsa-miR-199b-3p, hsa-miR-4685-3p). In addition, miRNAs observed in serum of PCOS-sons appear to regulate six PCOS-risk genes identified in GWASs. The *AOPEP* that encodes zinc-dependent metallopeptidase catalyzing the removal of an amino acid from a protein[30,33] is also linked to type 2 diabetes,[67] testosterone levels,[3] and endometrial cancer[68]; TOX3 encodes a nuclear protein of the high-mobility group (HMG) box family and is associated with insulin resistance and metabolic syndrome in women with PCOS[32,45]; *ERBB4* is a member of epidermal growth factor receptors (EGFRs) and is a risk locus for PCOS, BMI and visceral adipose tissue, type 2 diabetes, and epithelial ovarian cancer, as well as sperm motility[31,32,34,35,45]; the SNP rs1159315 is located near γ-aminobutyric acid A receptor β1 (*GABRB1*) and linked to PCOS, obesity-related traits, and depression;[46] *ADGRB3* is linked to BMI, triglyceride, adiponectin, and follicle-stimulating hormone levels in PCOS;[33] *MYRIP* is related to insulin levels and PCOS[36]; and *ADGRB3* is linked to BMI, triglyceride, adiponectin, and follicle-stimulating hormone levels in PCOS.[33]

These findings implicate that PCOS-sons may carry circulating factors that underlie PCOS susceptibility for the development of phenotype. Together with our recent findings that daughters of women with PCOS are five times more likely to be diagnosed with PCOS[6] and that prenatal androgen-exposed F_1 male offspring develop an aberrant reproductive and metabolic phenotype,[19,20] our current findings strengthen the hypothesis that maternal PCOS could induce fetal programming, predisposing not only daughters but also their sons to adult disease due to adverse maternal-fetal environment.

Recently, we and others showed that PCOS-like traits induced by maternal androgen or AMH exposure in mice can be passed down to the third generation in female offspring,[6,18] but it remains unexplored whether such traits in F_1 male offspring could be transmitted across generations in their male progeny. In agreement with the clinical findings, our mouse models confirmed that maternal obesity or prenatal androgen exposure affects their F_1 male offspring, resulting in reproductive dysfunctions with increased anogenital distance, decreased testis weight, and aberrant mitochondrial morphology. Moreover, metabolic dysfunctions in F_1 male offspring are presented by increased fat mass and epididymal adipocytes and altered energy metabolism in both obesity and androgenized lineages, except for impaired glucose homeostasis, which is only in the obese lineage. Of note, the indirect calorimetry results showed lower metabolic activity (RER and EE) both in light and dark phases in the F_1 and F_3 males in the androgenized, obese, and obese-androgenized lineages, respectively. Importantly, the decrease in RER and EE is independent of food intake and total activity, suggesting that it is caused by increased body weight and fat mass. Thus, the lower metabolic activities (RER and EE) are likely consequences of *in utero* programming, either by androgen- and/or diet-induced obesity, resulting in inheritable metabolic pattern alterations such as different preferences of energy substrate.

The F_1 male offspring of the prenatal androgenized lineage did not present with any differences in circulating testosterone or sperm count, which agrees with a recent study by Holland et al.[69] However, their study contrasts our finding of decreased testis weight as they found no such effect. But they did not measure anogenital distance or testis mitochondrial morphology or perform any molecular analyses of the sperm. Instead, they found unchanged luteinizing hormone (LH) secretion and density of gonadotropin-releasing hormone-expressing (GnRH) neurons, which strengthens our hypothesis that the transgenerational effects we observed in the androgenized lineage are likely mediated through the germline. More importantly, metabolic phenotypes were passed down to F_3 male offspring in both the androgenized and the obese lineage, while reproductive phenotype transmission occurred only in the obese lineage, which suggests germline reprogramming is likely more maintained in the obese lineage compared with the androgenized lineage. We speculate that male offspring (male germline) are more tolerant to maternal androgen exposure but more sensitive to maternal obesity exposure. Intriguingly, the combined effects of maternal obesity and prenatal androgenization have belated effects often presented only in F_3 male offspring. In contrast, there are no F_3 females surviving in the obese and androgenized lineage for analysis in our previous study,[6] which additionally indicates that the maternal uterine environment may have sex-dimorphic effects on offspring health. Future studies are warranted to identify the mechanism underlying sex dimorphism as well as distinct roles of the uterine environment versus germ cells in the transgenerational inheritance of disease.

Several studies demonstrated that the sperm small RNAs can also act as an epigenetic vehicle in transmitting phenotypes to the offspring. Mammalian sperm contain a complex repertoire of different types of sncRNAs in addition to haploid DNA.[70,71] These sncRNAs, including miRNAs, piRNAs, rsRNAs, and tsRNAs, regulate early embryonic development upon fertilization and act as a causal factor of intergenerational transmission of ancestral exposure phenotypes via germlines.[71] Maternal and paternal endocrine disruptors and nutrient disturbance result in inter/transgenerational epigenetic inheritance mediated by sperm sncRNAs.[27] The small RNA profiling of sperms in male offspring in the androgenized, obese, and combined lineages showed abundant transgenerational DEsncRNAs. Although we did not investigate the causal effects of DEsncRNAs, the majority of the 28S RNA-derived rsRNAs and Glu, Gly, and Val tRNA-derived tsRNAs have previously been reported to be involved in intergenerational and transgenerational transmission of disease such as stress response.[72–74] Moreover, epigenetic information can be delivered from somatic tissues to gametes via sncRNAs, providing a route for external stimuli depositing changes in the germline epigenomes.[74–77] Here, we identified common DEsncRNAs in serum of PCOS-sons and mouse sperms across generations, including the tsRNA-Val-CAC 5′ species, which previously has been reported to be absorbed

Representative pathways with $\log_{10}$ (p value) >2 are presented. Size of the bubble represents the number of genes enriched in the pathway. Orange color represents up-regulated expression of DEsncRNA in human and mouse, while light yellow color represents down-regulation. CD+Veh (F_1: n = 4, litters = 4; F_2: n = 4, litters = 4; F_3: n = 4, litters = 4); CD+DHT (F_1: n = 3, litters = 3; F_2: n = 4, litters = 4; F_3: n = 3, litters = 3); HFHS+Veh (F_1: n = 4, litters = 4; F_2: n = 4, litters = 4; F_3: n = 4, litters = 4); and HFHS+DHT (F_1: n = 4, litters = 4; F_2: n = 4, litters = 4; F_3: n = 4, litters = 4).

from the epididymosomes into the sperm, highlighting a potential bridge between the somatic cells and the germ cells.[75–77] Such interplay could also explain the intriguing inconsistent phenotypic features in the F_2 male offspring. Despite a weak reproductive and metabolic phenotype in F_2, they have alterations in sperm sncRNA profiles contributing to the transmission of aberrant phenotypes into subsequent generations. We did also observe DE X-linked spermiRs in androgenized and obese lineage across generations. Moreover, we identified several common biological processes between DEmiRNA predicted target genes in PCOS-sons and sperm of F_1 male offspring in respective lineage. Several of the predicted target genes have previously been shown to be of biological relevance in women with PCOS. For example, in PCOS-sons, *EDN1*, the target gene of hsa-miR-4685-3p, has been shown to be dysregulated in granulosa cells of women with PCOS; *CDK6*, the target gene of hsa-miR-1299, is a cell cycle marker likely to be involved in the development of PCOS; *FRS*2, a target gene of hsa-miR-96-5p, has been reported to be upregulated in obese women with PCOS and involved in insulin signaling; and *ETS1*, a target gene of hsa-miR-1299, is involved in androgen-mediated DNA methylation in granulosa cells in PCOS. All these genes in F_1 sperm are targets of mmu-miR-1a-3p.

Taken together, our comprehensive analyses have defined long-term adverse effects of obesity and prenatal androgen excess during pregnancy leading to transgenerational transmission of metabolic and reproductive traits in male progeny and epigenetic modifications in the sperm accompanying the phenotype transmission across generations. Importantly, several molecular signatures are detectible in serum from PCOS-sons, supporting the possibility of epigenetic inheritance in humans. These findings strengthen the translational significance identified in mouse models and shed light on a previously underappreciated risk of reproductive and metabolic alterations across generations via the male germline.

Limitations of the study

The overall percentage of women having PCOS in the Swedish Medical Birth Register and National Patient register is low because many women with PCOS are undiagnosed. This has gradually changed over the last 10 years, but the percentage is still much lower than in other countries and cannot be used as an estimate of prevalence. In this study, 2.1% of the mothers have PCOS, which is lower than the expected 10%–18% in the general population of reproductive age.[37,78,79] We have no explanation why F_2 in most of the measured variables seems to be unaffected whereas the phenotype occurs again in F_3. That phenotypic changes skip one (or even two) generation has previously been observed in the female and male germline by us and others.[6,49,50] Recently it was shown that F_0 males of a depression-like model can transmit the depressive traits to the F_1 but not the F_2 generation, which may result from the lack of miRNA changes in the depression lineage of F_1 sperm under baseline condition.[80] This phenomenon can be referred to as adaptive effects by the initial exposed germline in F_0.[81] Importantly, sncRNA alteration is in agreement with overall phenotypes, with the greatest number of DEsncRNAs found in the obese lineage. The variation in the F_1, F_2, and F_3 control groups, where the F_2 controls often differ from F_1 and F_3 male offspring, could explain the non-significant effects in F_2.

STAR★METHODS

Detailed methods are provided in the online version of this paper and include the following:

- KEY RESOURCES TABLE
- RESOURCE AVAILABILITY
    - Lead contact
    - Materials availability
    - Data and code availability
- EXPERIMENTAL MODEL AND SUBJECT DETAILS
    - Ethical approvals
    - Register based study
    - Human case-control study population
    - Animals
    - Mouse-breeding scheme and feeding paradigm to generate F_1 to F_3 offspring
- METHOD DETAILS
    - Assessment of reproductive phenotype
    - Assessment of metabolic phenotype
    - Biochemical assessment of serum insulin and sex steroids
    - Adipocyte size measurement
    - Liver triglycerides quantification
    - Testis AMH quantification
    - Liver oil red O staining
    - RNA isolation from testis and mRNA expression of mitochondrial genes
    - Collection of organs and isolation of motile spermatozoa
    - Transmission electron microscopy (TEM) for mitochondrial morphology
    - Small non-coding RNA library preparation
    - sncRNA-seq data processing
    - Differentially expressed gene analyses
    - Identification of common DEmiRNAs in women with PCOS and in sons of PCOS women
    - Human and mouse DEsncRNAs alignment
    - Target gene prediction and gene ontology analyses
- QUANTIFICATION AND STATISTICAL ANALYSIS

SUPPLEMENTAL INFORMATION

Supplemental information can be found online at https://doi.org/10.1016/j.xcrm.2023.101035.

ACKNOWLEDGMENTS

We thank Zhiyi Zhao, Jacob Victorin, Sonja Edström, and Sara Pilström for technical assistance during animal work and molecular analysis; TSE Systems and the Metabolic Phenotyping Center at the Strategic Research program in Diabetes at the Karolinska Institutet; and the electron microscopy unit Emil at Huddinge University Hospital at the Karolinska Institutet. This work is supported by the Swedish Medical Research Council: project nos. 2018-02435 and 2022-00550 (E.S.-V.) and 2018-02557 and 2020-00253 (Q.D.); the Knut and Alice Wallenberg Foundation: 2019.0211 (Q.D.); Distinguished Investigator Grant – Endocrinology and Metabolism, Novo Nordisk

Foundation: NNF22OC0072904 (E.S.-V.); the Diabetes Foundation: DIA2021-633 (E.S.-V.); the Novo Nordisk Foundation: NNF18OC0033992 and NNF19OC0056647 (E.S.-V.); the Strategic Research Program in Diabetes at the Karolinska Institutet (E.S.-V.); the Adlerbertska Research Foundation: GU 2019/86 (E.S.-V.); Karolinska Institutet KID funding: 2020-00990 (E.S.-V.); a Karolinska Instiutet faculty funded position (Q.D.); the Regional Agreement on Medical Training and Clinical Research between the Stockholm County Council and the Karolinska Institutet: 20190079 (E.S.-V.); O. E. och Edla Johanssons Stiftelse 2021 (S.R.); theKarolinska Institutet-China scholarship council program (Q.L.); Magnus Bergvalls Stiftelse: 2020-03808 and 2021-04329 (S.R.); the Karolinska Institutet: 2020-02026 (S.R.); theNational Fund for Scientific and Technological Development (FONDECYT): project no. 1151531 (T.S.P.); the FONDECYT: project no. 1201483 (B.E.); the National Commission for Scientific and Technological Research (CONICYT) (R.F.); HKH Kronprinsessan Lovisas förening för barnasjukvård (R.F.); and Stiftelsen Axel Tielmans minnesfond (R.F.).

AUTHOR CONTRIBUTIONS

Conceptualization, E.S.-V., Q.D., and S.R.; methodology, S.R., C.L., Q.L., R.F., N.B., A.B., J.Q., Q.D., and E.S.-V.; investigation, S.R., C.L., Q.L., R.F., H.L., G.E., E.L., N.C., M.M., B.E., S.R., T.S.P., and C.O.; visualization, S.R., C.L., Q.L., and R.F.; funding acquisition, E.S.-V., Q.D., S.R., and R.F.; project administration, E.S.-V. and Q.D.; supervision, E.S.-V. and Q.D.; writing – original draft, E.S.-V., Q.D., S.R., C.L., Q.L., R.F., N.B., N.C., M.M., S.R., T.S.P., C.O., A.B., and J.Q.; writing – review & editing, E.S.-V., Q.D., S.R., C.L., and Q.L.

DECLARATION OF INTERESTS

The authors declare no competing interests.

INCLUSION AND DIVERSITY

We support inclusive, diverse, and equitable conduct of research.

Received: June 3, 2022
Revised: November 27, 2022
Accepted: April 11, 2023
Published: May 5, 2023

REFERENCES

1. Dumesic, D.A., Oberfield, S.E., Stener-Victorin, E., Marshall, J.C., Laven, J.S., and Legro, R.S. (2015). Scientific statement on the diagnostic criteria, epidemiology, pathophysiology, and molecular genetics of polycystic ovary syndrome. Endocr. Rev. *36*, 487–525. https://doi.org/10.1210/er.2015-1018.
2. Cesta, C.E., Månsson, M., Palm, C., Lichtenstein, P., Iliadou, A.N., and Landén, M. (2016). Polycystic ovary syndrome and psychiatric disorders: Co-morbidity and heritability in a nationwide Swedish cohort. Psychoneuroendocrinology *73*, 196–203. https://doi.org/10.1016/j.psyneuen.2016.08.005.
3. Ruth, K.S., Day, F.R., Tyrrell, J., Thompson, D.J., Wood, A.R., Mahajan, A., Beaumont, R.N., Wittemans, L., Martin, S., Busch, A.S., et al. (2020). Using human genetics to understand the disease impacts of testosterone in men and women. Nat. Med. *26*, 252–258. https://doi.org/10.1038/s41591-020-0751-5.
4. Kataoka, J., Larsson, I., Björkman, S., Eliasson, B., Schmidt, J., and Stener-Victorin, E. (2019). Prevalence of polycystic ovary syndrome in women with severe obesity - effects of a structured weight loss programme. Clin. Endocrinol. *91*, 750–758. https://doi.org/10.1111/cen.14098.
5. Stener Victorin, E., and Deng, Q. (2021). Epigenetic inheritance of polycystic ovary syndrome – challenges and opportunities for treatment. Nat. Rev. Endocrinol. *17*, 521–533. https://doi.org/10.1038/s41574-021-00517-x.
6. Risal, S., Pei, Y., Lu, H., Manti, M., Fornes, R., Pui, H.P., Zhao, Z., Massart, J., Ohlsson, C., Lindgren, E., et al. (2019). Prenatal androgen exposure and transgenerational susceptibility to polycystic ovary syndrome. Nat. Med. *25*, 1894–1904. https://doi.org/10.1038/s41591-019-0666-1.
7. Crisosto, N., Echiburú, B., Maliqueo, M., Luchsinger, M., Rojas, P., Recabarren, S., and Sir-Petermann, T. (2017). Reproductive and metabolic features during puberty in sons of women with polycystic ovary syndrome. Endocr. Connect. *6*, 607–613. https://doi.org/10.1530/EC-17-0218.
8. Recabarren, S.E., Smith, R., Rios, R., Maliqueo, M., Echiburú, B., Codner, E., Cassorla, F., Rojas, P., and Sir-Petermann, T. (2008). Metabolic profile in sons of women with polycystic ovary syndrome. J. Clin. Endocrinol. Metab. *93*, 1820–1826. https://doi.org/10.1210/jc.2007-2256.
9. Recabarren, S.E., Sir-Petermann, T., Rios, R., Maliqueo, M., Echiburú, B., Smith, R., Rojas-García, P., Recabarren, M., and Rey, R.A. (2008). Pituitary and testicular function in sons of women with polycystic ovary syndrome from infancy to adulthood. J. Clin. Endocrinol. Metab. *93*, 3318–3324. https://doi.org/10.1210/jc.2008-0255 jc.2008-0255.
10. Liu, D.M., Torchen, L.C., Sung, Y., Paparodis, R., Legro, R.S., Grebe, S.K., Singh, R.J., Taylor, R.L., and Dunaif, A. (2014). Evidence for gonadotrophin secretory and steroidogenic abnormalities in brothers of women with polycystic ovary syndrome. Hum. Reprod. *29*, 2764–2772. https://doi.org/10.1093/humrep/deu282.
11. Torchen, L.C., Kumar, A., Kalra, B., Savjani, G., Sisk, R., Legro, R.S., and Dunaif, A. (2016). Increased antimullerian hormone levels and other reproductive endocrine changes in adult male relatives of women with polycystic ovary syndrome. Fertil. Steril. *106*, 50–55. https://doi.org/10.1016/j.fertnstert.2016.03.029.
12. Sam, S., Coviello, A.D., Sung, Y.A., Legro, R.S., and Dunaif, A. (2008). Metabolic phenotype in the brothers of women with polycystic ovary syndrome. Diabetes Care *31*, 1237–1241. https://doi.org/10.2337/dc07-2190.
13. Sam, S., Sung, Y.A., Legro, R.S., and Dunaif, A. (2008). Evidence for pancreatic beta-cell dysfunction in brothers of women with polycystic ovary syndrome. Metabolism *57*, 84–89.
14. Baillargeon, J.P., and Carpentier, A.C. (2007). Brothers of women with polycystic ovary syndrome are characterised by impaired glucose tolerance, reduced insulin sensitivity and related metabolic defects. Diabetologia *50*, 2424–2432. https://doi.org/10.1007/s00125-007-0831-9.
15. Subramaniam, K., Tripathi, A., and Dabadghao, P. (2019). Familial clustering of metabolic phenotype in brothers of women with polycystic ovary syndrome. Gynecol. Endocrinol. *35*, 601–603. https://doi.org/10.1080/09513590.2019.1566451.
16. Karthik, S., Vipin, V.P., Kapoor, A., Tripathi, A., Shukla, M., and Dabadghao, P. (2019). Cardiovascular disease risk in the siblings of women with polycystic ovary syndrome. Hum. Reprod. *34*, 1559–1566. https://doi.org/10.1093/humrep/dez104.
17. Zhu, J., Pujol-Gualdo, N., Wittemans, L.B.L., Lindgren, C.M., Laisk, T., Hirschhorn, J.N., and Chan, Y.M. (2022). Evidence from men for ovary-independent effects of genetic risk factors for polycystic ovary syndrome. J. Clin. Endocrinol. Metab. *107*, e1577–e1587. https://doi.org/10.1210/clinem/dgab838.
18. Mimouni, N.E.H., Paiva, I., Barbotin, A.L., Timzoura, F.E., Plassard, D., Le Gras, S., Ternier, G., Pigny, P., Catteau-Jonard, S., Simon, V., et al. (2021). Polycystic ovary syndrome is transmitted via a transgenerational epigenetic process. Cell Metab. *33*, 513–530.e8. https://doi.org/10.1016/j.cmet.2021.01.004.
19. Scully, C.M., Estill, C.T., Amodei, R., McKune, A., Gribbin, K.P., Meaker, M., Stormshak, F., and Roselli, C.E. (2018). Early prenatal androgen exposure reduces testes size and sperm concentration in sheep without altering neuroendocrine differentiation and masculine sexual behavior. Domest. Anim. Endocrinol. *62*, 1–9. https://doi.org/10.1016/j.domaniend.2017.07.001.

20. Veiga-Lopez, A., Steckler, T.L., Abbott, D.H., Welch, K.B., MohanKumar, P.S., Phillips, D.J., Refsal, K., and Padmanabhan, V. (2011). Developmental programming: impact of excess prenatal testosterone on intrauterine fetal endocrine milieu and growth in sheep. Biol. Reprod. *84*, 87–96. https://doi.org/10.1095/biolreprod.110.086686.

21. Siemienowicz, K.J., Filis, P., Shaw, S., Douglas, A., Thomas, J., Mulroy, S., Howie, F., Fowler, P.A., Duncan, W.C., and Rae, M.T. (2019). Fetal androgen exposure is a determinant of adult male metabolic health. Sci. Rep. *9*, 20195. https://doi.org/10.1038/s41598-019-56790-4.

22. More, A.S., Mishra, J.S., Gopalakrishnan, K., Blesson, C.S., Hankins, G.D., and Sathishkumar, K. (2016). Prenatal testosterone exposure leads to gonadal hormone-dependent hyperinsulinemia and gonadal hormone-independent glucose intolerance in adult male rat offspring. Biol. Reprod. *94*, 5. https://doi.org/10.1095/biolreprod.115.133157.

23. Lazic, M., Aird, F., Levine, J.E., and Dunaif, A. (2011). Prenatal androgen treatment alters body composition and glucose homeostasis in male rats. J. Endocrinol. *208*, 293–300. https://doi.org/10.1677/JOE-10-0263JOE-10-0263.

24. Crews, D., Gillette, R., Scarpino, S.V., Manikkam, M., Savenkova, M.I., and Skinner, M.K. (2012). Epigenetic transgenerational inheritance of altered stress responses. Proc. Natl. Acad. Sci. USA *109*, 9143–9148. https://doi.org/10.1073/pnas.1118514109.

25. Manikkam, M., Tracey, R., Guerrero-Bosagna, C., and Skinner, M.K. (2012). Pesticide and insect repellent mixture (permethrin and DEET) induces epigenetic transgenerational inheritance of disease and sperm epimutations. Reprod. Toxicol. *34*, 708–719. https://doi.org/10.1016/j.reprotox.2012.08.010.

26. Guerrero-Bosagna, C., Covert, T.R., Haque, M.M., Settles, M., Nilsson, E.E., Anway, M.D., and Skinner, M.K. (2012). Epigenetic transgenerational inheritance of vinclozolin induced mouse adult onset disease and associated sperm epigenome biomarkers. Reprod. Toxicol. *34*, 694–707. https://doi.org/10.1016/j.reprotox.2012.09.005.

27. Gapp, K., Jawaid, A., Sarkies, P., Bohacek, J., Pelczar, P., Prados, J., Farinelli, L., Miska, E., and Mansuy, I.M. (2014). Implication of sperm RNAs in transgenerational inheritance of the effects of early trauma in mice. Nat. Neurosci. *17*, 667–669. https://doi.org/10.1038/nn.3695.

28. Crisóstomo, L., Bourgery, M., Rato, L., Raposo, J.F., Batterham, R.L., Kotaja, N., and Alves, M.G. (2022). Testicular "inherited metabolic memory" of ancestral high-fat diet is associated with sperm sncRNA content. Biomedicines *10*, 909. https://doi.org/10.3390/biomedicines10040909.

29. Crisóstomo, L., Videira, R.A., Jarak, I., Starčević, K., Mašek, T., Rato, L., Raposo, J.F., Batterham, R.L., Oliveira, P.F., and Alves, M.G. (2022). Inherited metabolic memory of high-fat diet impairs testicular fatty acid content and sperm parameters. Mol. Nutr. Food Res. *66*, e2100680. https://doi.org/10.1002/mnfr.202100680.

30. Shi, Y., Zhao, H., Shi, Y., Cao, Y., Yang, D., Li, Z., Zhang, B., Liang, X., Li, T., Chen, J., et al. (2012). Genome-wide association study identifies eight new risk loci for polycystic ovary syndrome. Nat. Genet. *44*, 1020–1025. https://doi.org/10.1038/ng.2384 ng.2384.

31. Day, F.R., Ruth, K.S., Thompson, D.J., Lunetta, K.L., Pervjakova, N., Chasman, D.I., Stolk, L., Finucane, H.K., Sulem, P., Bulik-Sullivan, B., et al. (2015). Large-scale genomic analyses link reproductive aging to hypothalamic signaling, breast cancer susceptibility and BRCA1-mediated DNA repair. Nat. Genet. *47*, 1294–1303. https://doi.org/10.1038/ng.3412.

32. Day, F., Karaderi, T., Jones, M.R., Meun, C., He, C., Drong, A., Kraft, P., Lin, N., Huang, H., Broer, L., et al. (2018). Large-scale genome-wide meta-analysis of polycystic ovary syndrome suggests shared genetic architecture for different diagnosis criteria. PLoS Genet. *14*, e1007813. https://doi.org/10.1371/journal.pgen.1007813.

33. Hayes, M.G., Urbanek, M., Ehrmann, D.A., Armstrong, L.L., Lee, J.Y., Sisk, R., Karaderi, T., Barber, T.M., McCarthy, M.I., Franks, S., et al. (2015). Genome-wide association of polycystic ovary syndrome implicates alterations in gonadotropin secretion in European ancestry populations. Nat. Commun. *6*, 7502. https://doi.org/10.1038/ncomms8502.

34. Zhang, Y., Ho, K., Keaton, J.M., Hartzel, D.N., Day, F., Justice, A.E., Josyula, N.S., Pendergrass, S.A., Actkins, K., Davis, L.K., et al. (2020). A genome-wide association study of polycystic ovary syndrome identified from electronic health records. Am. J. Obstet. Gynecol. *223*, 559.e1-559559.e21. https://doi.org/10.1016/j.ajog.2020.04.004.

35. Tyrmi, J.S., Arffman, R.K., Pujol-Gualdo, N., Kurra, V., Morin-Papunen, L., Sliz, E., FinnGen Consortium Estonian Biobank Research Team; Piltonen, T.T., Laisk, T., Kettunen, J., and Laivuori, H. (2022). Leveraging Northern European population history: novel low-frequency variants for polycystic ovary syndrome. Hum. Reprod. *37*, 352–365. https://doi.org/10.1093/humrep/deab250.

36. Lim, W.Y., Lee, H., and Cho, Y.S. (2021). Identification of genetic variants for blood insulin level in sex-stratified Korean population and evaluation of the causal relationship between blood insulin level and polycystic ovary syndrome. Genes Genomics *43*, 1105–1117. https://doi.org/10.1007/s13258-021-01134-8.

37. Joham, A.E., Norman, R.J., Stener-Victorin, E., Legro, R.S., Franks, S., Moran, L.J., Boyle, J., and Teede, H.J. (2022). Polycystic ovary syndrome. Lancet Diabetes Endocrinol. *10*, 668–680. https://doi.org/10.1016/S2213-8587(22)00163-2.

38. Zhang, F., Li, S.P., Zhang, T., Yu, B., Zhang, J., Ding, H.G., Ye, F.J., Yuan, H., Ma, Y.Y., Pan, H.T., and He, Y. (2021). High throughput microRNAs sequencing profile of serum exosomes in women with and without polycystic ovarian syndrome. PeerJ *9*, e10998. https://doi.org/10.7717/peerj.10998.

39. Udesen, P.B., Glintborg, D., Sørensen, A.E., Svendsen, R., Nielsen, N.L.S., Wissing, M.L.M., Andersen, M.S., Englund, A.L.M., and Dalgaard, L.T. (2020). Metformin decreases miR-122, miR-223 and miR-29a in women with polycystic ovary syndrome. Endocr. Connect. *9*, 1075–1084. https://doi.org/10.1530/EC-20-0195.

40. Butler, A.E., Ramachandran, V., Hayat, S., Dargham, S.R., Cunningham, T.K., Benurwar, M., Sathyapalan, T., Najafi-Shoushtari, S.H., and Atkin, S.L. (2019). Expression of microRNA in follicular fluid in women with and without PCOS. Sci. Rep. *9*, 16306. https://doi.org/10.1038/s41598-019-52856-5.

41. Hu, M.H., Zheng, S.X., Yin, H., Zhu, X.Y., Lu, F.T., Tong, X.H., Liu, Y.S., Zhang, Y.W., and Xu, B. (2020). Identification of microRNAs that regulate the MAPK pathway in human cumulus cells from PCOS women with insulin resistance. Reprod. Sci. *27*, 833–844. https://doi.org/10.1007/s43032-019-00086-5.

42. Shao, S., Wang, H., Shao, W., and Liu, N. (2020). miR-199a-5p stimulates ovarian granulosa cell apoptosis in polycystic ovary syndrome. J. Mol. Endocrinol. *65*, 187–201. https://doi.org/10.1530/JME-20-0077.

43. Li, Y., Fang, Y., Liu, Y., and Yang, X. (2015). MicroRNAs in ovarian function and disorders. J. Ovarian Res. *8*, 51. https://doi.org/10.1186/s13048-015-0162-2.

44. Hossain, M.M., Cao, M., Wang, Q., Kim, J.Y., Schellander, K., Tesfaye, D., and Tsang, B.K. (2013). Altered expression of miRNAs in a dihydrotestosterone-induced rat PCOS model. J. Ovarian Res. *6*, 36. https://doi.org/10.1186/1757-2215-6-36.

45. Tian, Y., Li, J., Su, S., Cao, Y., Wang, Z., Zhao, S., and Zhao, H. (2020). PCOS-GWAS susceptibility variants in THADA, INSR, TOX3, and DENND1A are associated with metabolic syndrome or insulin resistance in women with PCOS. Front. Endocrinol. *11*, 274. https://doi.org/10.3389/fendo.2020.00274.

46. Kim, S.H., Liu, M., Jin, H.S., and Park, S. (2019). High genetic risk scores of ASIC2, MACROD2, CHRM3, and C2orf83 genetic variants associated with polycystic ovary syndrome impair insulin sensitivity and interact with energy intake in Korean women. Gynecol. Obstet. Invest. *84*, 225–236. https://doi.org/10.1159/000493131.

47. Barrett, E.S., Hoeger, K.M., Sathyanarayana, S., Abbott, D.H., Redmon, J.B., Nguyen, R.H.N., and Swan, S.H. (2018). Anogenital distance in newborn daughters of women with polycystic ovary syndrome indicates

fetal testosterone exposure. J. Dev. Orig. Health Dis. *9*, 307–314. https://doi.org/10.1017/S2040174417001118.

48. Jiang, M., Kauppila, T.E.S., Motori, E., Li, X., Atanassov, I., Folz-Donahue, K., Bonekamp, N.A., Albarran-Gutierrez, S., Stewart, J.B., and Larsson, N.G. (2017). Increased total mtDNA copy number cures male infertility despite unaltered mtDNA mutation load. Cell Metab. *26*, 429–436.e4. https://doi.org/10.1016/j.cmet.2017.07.003.

49. Aiken, C.E., and Ozanne, S.E. (2014). Transgenerational developmental programming. Hum. Reprod. Update *20*, 63–75. https://doi.org/10.1093/humupd/dmt043.

50. Risal, S., Manti, M., Lu, H., Fornes, R., Larsson, H., Benrick, A., Deng, Q., Cesta, C.E., Rosenqvist, M.A., and Stener-Victorin, E. (2021). Prenatal androgen exposure causes a sexually dimorphic transgenerational increase in offspring susceptibility to anxiety disorders. Transl. Psychiatry *11*, 45. https://doi.org/10.1038/s41398-020-01183-9.

51. Beyret, E., Liu, N., and Lin, H. (2012). piRNA biogenesis during adult spermatogenesis in mice is independent of the ping-pong mechanism. Cell Res. *22*, 1429–1439. https://doi.org/10.1038/cr.2012.120.

52. Aravin, A.A., Sachidanandam, R., Bourc'his, D., Schaefer, C., Pezic, D., Toth, K.F., Bestor, T., and Hannon, G.J. (2008). A piRNA pathway primed by individual transposons is linked to de novo DNA methylation in mice. Mol. Cell *31*, 785–799. https://doi.org/10.1016/j.molcel.2008.09.003.

53. Aravin, A.A., Sachidanandam, R., Girard, A., Fejes-Toth, K., and Hannon, G.J. (2007). Developmentally regulated piRNA clusters implicate MILI in transposon control. Science *316*, 744–747. https://doi.org/10.1126/science.1142612.

54. Gou, L.T., Dai, P., Yang, J.H., Xue, Y., Hu, Y.P., Zhou, Y., Kang, J.Y., Wang, X., Li, H., Hua, M.M., et al. (2014). Pachytene piRNAs instruct massive mRNA elimination during late spermiogenesis. Cell Res. *24*, 680–700. https://doi.org/10.1038/cr.2014.41.

55. Zhang, F., Zhang, Y., Lv, X., Xu, B., Zhang, H., Yan, J., Li, H., and Wu, L. (2019). Evolution of an X-linked miRNA family predominantly expressed in mammalian male germ cells. Mol. Biol. Evol. *36*, 663–678. https://doi.org/10.1093/molbev/msz001.

56. Chen, J., Spracklen, C.N., Marenne, G., Varshney, A., Corbin, L.J., Luan, J., Willems, S.M., Wu, Y., Zhang, X., Horikoshi, M., et al. (2021). The trans-ancestral genomic architecture of glycemic traits. Nat. Genet. *53*, 840–860. https://doi.org/10.1038/s41588-021-00852-9.

57. Flannick, J., and Florez, J.C. (2016). Type 2 diabetes: genetic data sharing to advance complex disease research. Nat. Rev. Genet. *17*, 535–549. https://doi.org/10.1038/nrg.2016.56.

58. Dapas, M., and Dunaif, A. (2022). Deconstructing a syndrome: genomic insights into PCOS causal mechanisms and classification. Endocr. Rev. *43*, 927–965. https://doi.org/10.1210/endrev/bnac001.

59. Ding, C.F., Chen, W.Q., Zhu, Y.T., Bo, Y.L., Hu, H.M., and Zheng, R.H. (2015). Circulating microRNAs in patients with polycystic ovary syndrome. Hum. Fertil. *18*, 22–29. https://doi.org/10.3109/14647273.2014.956811.

60. Jiang, L., Li, W., Wu, M., and Cao, S. (2015). Ciculating miRNA-21 as a biomarker predicts polycystic ovary syndrome (PCOS) in patients. Clin. Lab. *61*, 1009–1015. https://doi.org/10.7754/clin.lab.2015.150122.

61. Jiang, X., Li, J., Zhang, B., Hu, J., Ma, J., Cui, L., and Chen, Z.J. (2021). Differential expression profile of plasma exosomal microRNAs in women with polycystic ovary syndrome. Fertil. Steril. *115*, 782–792. https://doi.org/10.1016/j.fertnstert.2020.08.019.

62. Murri, M., Insenser, M., Fernández-Durán, E., San-Millán, J.L., Luque-Ramírez, M., and Escobar-Morreale, H.F. (2018). Non-targeted profiling of circulating microRNAs in women with polycystic ovary syndrome (PCOS): effects of obesity and sex hormones. Metabolism *86*, 49–60. https://doi.org/10.1016/j.metabol.2018.01.011.

63. Sathyapalan, T., David, R., Gooderham, N.J., and Atkin, S.L. (2015). Increased expression of circulating miRNA-93 in women with polycystic ovary syndrome may represent a novel, non-invasive biomarker for diagnosis. Sci. Rep. *5*, 16890. https://doi.org/10.1038/srep16890.

64. Song, J., Luo, S., and Li, S.W. (2015). miRNA-592 is downregulated and may target LHCGR in polycystic ovary syndrome patients. Reprod. Biol. *15*, 229–237. https://doi.org/10.1016/j.repbio.2015.10.005.

65. Xiong, W., Lin, Y., Xu, L., Tamadon, A., Zou, S., Tian, F., Shao, R., Li, X., and Feng, Y. (2017). Circulatory microRNA 23a and microRNA 23b and polycystic ovary syndrome (PCOS): the effects of body mass index and sex hormones in an Eastern Han Chinese population. J. Ovarian Res. *10*, 10. https://doi.org/10.1186/s13048-016-0298-8.

66. Zhao, C., Liu, X., Shi, Z., Zhang, J., Zhang, J., Jia, X., and Ling, X. (2015). Role of serum miRNAs in the prediction of ovarian hyperstimulation syndrome in polycystic ovarian syndrome patients. Cell. Physiol. Biochem. *35*, 1086–1094. https://doi.org/10.1159/000373934.

67. Vujkovic, M., Keaton, J.M., Lynch, J.A., Miller, D.R., Zhou, J., Tcheandjieu, C., Huffman, J.E., Assimes, T.L., Lorenz, K., Zhu, X., et al. (2020). Discovery of 318 new risk loci for type 2 diabetes and related vascular outcomes among 1.4 million participants in a multi-ancestry meta-analysis. Nat. Genet. *52*, 680–691. https://doi.org/10.1038/s41588-020-0637-y.

68. Cheng, T.H.T., Thompson, D., Painter, J., O'Mara, T., Gorman, M., Martin, L., Palles, C., Jones, A., Buchanan, D.D., Win, A.K., et al. (2015). Meta-analysis of genome-wide association studies identifies common susceptibility polymorphisms for colorectal and endometrial cancer near SH2B3 and TSHZ1. Sci. Rep. *5*, 17369. https://doi.org/10.1038/srep17369.

69. Holland, S., Prescott, M., Pankhurst, M., and Campbell, R.E. (2019). The influence of maternal androgen excess on the male reproductive axis. Sci. Rep. *9*, 18908. https://doi.org/10.1038/s41598-019-55436-9.

70. Chen, Q., Yan, M., Cao, Z., Li, X., Zhang, Y., Shi, J., Feng, G.H., Peng, H., Zhang, X., Zhang, Y., et al. (2016). Sperm tsRNAs contribute to intergenerational inheritance of an acquired metabolic disorder. Science *351*, 397–400. https://doi.org/10.1126/science.aad7977.

71. Zhang, Y., Shi, J., Rassoulzadegan, M., Tuorto, F., and Chen, Q. (2019). Sperm RNA code programmes the metabolic health of offspring. Nat. Rev. Endocrinol. *15*, 489–498. https://doi.org/10.1038/s41574-019-0226-2.

72. Zhang, Y., Zhang, X., Shi, J., Tuorto, F., Li, X., Liu, Y., Liebers, R., Zhang, L., Qu, Y., Qian, J., et al. (2018). Dnmt2 mediates intergenerational transmission of paternally acquired metabolic disorders through sperm small non-coding RNAs. Nat. Cell Biol. *20*, 535–540. https://doi.org/10.1038/s41556-018-0087-2.

73. Zhang, Y., Ren, L., Sun, X., Zhang, Z., Liu, J., Xin, Y., Yu, J., Jia, Y., Sheng, J., Hu, G.F., et al. (2021). Angiogenin mediates paternal inflammation-induced metabolic disorders in offspring through sperm tsRNAs. Nat. Commun. *12*, 6673. https://doi.org/10.1038/s41467-021-26909-1.

74. van Steenwyk, G., Gapp, K., Jawaid, A., Germain, P.L., Manuella, F., Tanwar, D.K., Zamboni, N., Gaur, N., Efimova, A., Thumfart, K.M., et al. (2020). Involvement of circulating factors in the transmission of paternal experiences through the germline. EMBO J. *39*, e104579. https://doi.org/10.15252/embj.2020104579.

75. Sharma, U., Sun, F., Conine, C.C., Reichholf, B., Kukreja, S., Herzog, V.A., Ameres, S.L., and Rando, O.J. (2018). Small RNAs are trafficked from the epididymis to developing mammalian sperm. Dev. Cell *46*, 481–494.e6. https://doi.org/10.1016/j.devcel.2018.06.023.

76. Conine, C.C., and Rando, O.J. (2022). Soma-to-germline RNA communication. Nat. Rev. Genet. *23*, 73–88. https://doi.org/10.1038/s41576-021-00412-1.

77. Conine, C.C., Sun, F., Song, L., Rivera-Pérez, J.A., and Rando, O.J. (2018). Small RNAs gained during epididymal transit of sperm are essential for embryonic development in mice. Dev. Cell *46*, 470–480.e3. https://doi.org/10.1016/j.devcel.2018.06.024.

78. Roos, N., Kieler, H., Sahlin, L., Ekman-Ordeberg, G., Falconer, H., and Stephansson, O. (2011). Risk of adverse pregnancy outcomes in women with polycystic ovary syndrome: population based cohort study. BMJ *343*, d6309. https://doi.org/10.1136/bmj.d6309 bmj.d6309.

CellPress
OPEN ACCESS

79. Lindholm, A., Andersson, L., Eliasson, M., Bixo, M., and Sundström-Poromaa, I. (2008). Prevalence of symptoms associated with polycystic ovary syndrome. Int. J. Gynaecol. Obstet. *102*, 39–43.

80. Wang, Y., Chen, Z.P., Hu, H., Lei, J., Zhou, Z., Yao, B., Chen, L., Liang, G., Zhan, S., Zhu, X., et al. (2021). Sperm microRNAs confer depression susceptibility to offspring. Sci. Adv. *7*, eabd7605. https://doi.org/10.1126/sciadv.abd7605.

81. Bale, T.L. (2015). Epigenetic and transgenerational reprogramming of brain development. Nat. Rev. Neurosci. *16*, 332–344. https://doi.org/10.1038/nrn3818.

82. Nilsson, M.E., Vandenput, L., Tivesten, Å., Norlén, A.K., Lagerquist, M.K., Windahl, S.H., Börjesson, A.E., Farman, H.H., Poutanen, M., Benrick, A., et al. (2015). Measurement of a comprehensive sex steroid profile in rodent serum by high-sensitive gas chromatography-tandem mass spectrometry. Endocrinology *156*, 2492–2502. https://doi.org/10.1210/en.2014-1890.

83. Benrick, A., Chanclón, B., Micallef, P., Wu, Y., Hadi, L., Shelton, J.M., Stener-Victorin, E., and Wernstedt Asterholm, I. (2017). Adiponectin protects against development of metabolic disturbances in a PCOS mouse model. Proc. Natl. Acad. Sci. USA *114*, E7187–E7196. https://doi.org/10.1073/pnas.1708854114.

84. Mehlem, A., Hagberg, C.E., Muhl, L., Eriksson, U., and Falkevall, A. (2013). Imaging of neutral lipids by oil red O for analyzing the metabolic status in health and disease. Nat. Protoc. *8*, 1149–1154. https://doi.org/10.1038/nprot.2013.055.

85. Martin, M. (2011). Cutadapt removes adapter sequences from high-throughput sequencing reads. Embnet. J *17*, 3. https://doi.org/10.14806/ej.17.1.200.

86. Shi, J., Ko, E.A., Sanders, K.M., Chen, Q., and Zhou, T. (2018). SPORTS1.0: a tool for annotating and profiling non-coding RNAs optimized for rRNA- and tRNA-derived small RNAs. Dev. Reprod. Biol. *16*, 144–151. https://doi.org/10.1016/j.gpb.2018.04.004.

87. Edgar, R.C. (2013). UPARSE: highly accurate OTU sequences from microbial amplicon reads. Nat. Methods *10*, 996–998. https://doi.org/10.1038/nmeth.2604.

88. Zhang, Y., Parmigiani, G., and Johnson, W.E. (2020). ComBat-seq: batch effect adjustment for RNA-seq count data. NAR Genom. Bioinform. *2*, lqaa078. https://doi.org/10.1093/nargab/lqaa078.

89. Hicks, S.C., Okrah, K., Paulson, J.N., Quackenbush, J., Irizarry, R.A., and Bravo, H.C. (2018). Smooth quantile normalization. Biostatistics *19*, 185–198. https://doi.org/10.1093/biostatistics/kxx028.

90. Robinson, M.D., McCarthy, D.J., and Smyth, G.K. (2010). edgeR: a Bioconductor package for differential expression analysis of digital gene expression data. Bioinformatics *26*, 139–140. https://doi.org/10.1093/bioinformatics/btp616.

91. Lambert, M., Benmoussa, A., and Provost, P. (2019). Small non-coding RNAs derived from eukaryotic ribosomal RNA. Noncoding. RNA *5*, 16. https://doi.org/10.3390/ncrna5010016.

92. Altschul, S.F., Madden, T.L., Schäffer, A.A., Zhang, J., Zhang, Z., Miller, W., and Lipman, D.J. (1997). Gapped BLAST and PSI-BLAST: a new generation of protein database search programs. Nucleic Acids Res. *25*, 3389–3402. https://doi.org/10.1093/nar/25.17.3389.

93. Zhang, D., Tu, S., Stubna, M., Wu, W.S., Huang, W.C., Weng, Z., and Lee, H.C. (2018). The piRNA targeting rules and the resistance to piRNA silencing in endogenous genes. Science *359*, 587–592. https://doi.org/10.1126/science.aao2840.

94. Zhou, Y., Zhou, B., Pache, L., Chang, M., Khodabakhshi, A.H., Tanaseichuk, O., Benner, C., and Chanda, S.K. (2019). Metascape provides a biologist-oriented resource for the analysis of systems-level datasets. Nat. Commun. *10*, 1523. https://doi.org/10.1038/s41467-019-09234-6.

95. Barlow, S.E.; Expert Committee (2007). Expert committee recommendations regarding the prevention, assessment, and treatment of child and adolescent overweight and obesity: summary report. Pediatrics *120* (*Suppl 4*), S164–S192. https://doi.org/10.1542/peds.2007-2329C.

96. Manti, M., Fornes, R., Qi, X., Folmerz, E., Lindén Hirschberg, A., de Castro Barbosa, T., Maliqueo, M., Benrick, A., and Stener-Victorin, E. (2018). Maternal androgen excess and obesity induce sexually dimorphic anxiety-like behavior in the offspring. Faseb. J. *32*, 4158–4171. https://doi.org/10.1096/fj.201701263RR.

97. Al Nabhani, Z., Dulauroy, S., Lécuyer, E., Polomack, B., Campagne, P., Berard, M., and Eberl, G. (2019). Excess calorie intake early in life increases susceptibility to colitis in adulthood. Nat. Metab. *1*, 1101–1109. https://doi.org/10.1038/s42255-019-0129-5.

STAR★METHODS

KEY RESOURCES TABLE

REAGENT or RESOURCE	SOURCE	IDENTIFIER
Biological samples		
Human Serum	Human	Crisosto et al., 2017[7]
Mouse Serum	Mouse	This paper
Mouse Sperm	Mouse	This paper
Chemicals, peptides, and recombinant proteins		
Benzyl benzoate	Sigma-Aldrich	Cat#B6630
Sesame oil	Sigma-Aldrich	Cat#S3547
DHT (5α androstane-17β-ol-3-one)	Sigma-Aldrich	Cat#A8380
NP-40 alternative	Merck KGaA	Cat#492016
TRI reagent	Sigma-Aldrich	Cat# T9424
Critical commercial assays		
Serum insulin (ELISA kit)	Crystal Chem	Cat# 90080
Triglyceride kit	Randox	Cat#TR210
High-Capacity RNA-to-cDNA kit	Applied Biosystems	Cat#4387406
NEB Next Multiplex Small RNA Library Prep Set for Illumina	NEB	Cat#E7300
DNA Clean & Concentrator-5 Kit	Zymo Research	Cat# D4003
PipPin Prep	Sage Science	Cat#CDP 3010
Polyacrylamide Gel	Life Technologies	Cat#EC6265BOX
Deposited data		
Raw sequencing reads of sncRNA-seq data of mouse sperm	This paper	Sequence Read Archive database via accession number PRJNA743232
Raw sequencing reads of sncRNA-seq data of human serum	This paper	Raw sequencing reads of sncRNA-seq data of human serum are available in The European Genome-Phenome Archive (EGA) via accession number EGAS00001007079
Raw data of F_1–F_3	This paper	Mendeley Data: https://doi.org/10.17632/4vhx9dk4st1.
Experimental models: Organisms/strains		
C57Bl/6J mice	Janvier Labs	RRID: IMSR_JAX:000664
Oligonucleotides		
Tfam **F**: ATTCCGAAGTGTTTTTCCAGCA; **R**: TCTGAAAGTTTTGCATCTGGGT	This paper	N/A
Drp1 **F**: CAGGAATTGTTACGGTTCCCTAA; **R**: CCTGAATTAACTTGTCCCGTG	This paper	N/A
Opa1 **F**: TGGAAAATGGTTCGAGAGTCAG; **R**: CATTCCGTCTCTAGGTTAAAGCG	This paper	N/A
Guf1 **F**: GCTTTCTGATTGCTGGGATGA; **R**: TGCAAACACCATTGGTTTCGC	This paper	N/A
Software and algorithms		
ANODE	R package car	RRID: SCR_022137
Stata statistical software version 14.0	Stata Corps	RRID: SCR_012763
GraphPad Prism 8	GraphPad Software	RRID: SCR_002798
Other		
UCSC genome GRCm38 for mouse and GRCh38 for human.	miRbase 22.1, rRNA from NCBI/Nucleotide, GtRNAdb, piRBase and piRNA bank, ensembl (release-89) ncRNA, and rfam 12.3	N/A

CellPress
OPEN ACCESS

RESOURCE AVAILABILITY

Lead contact

Further information and requests for resources and reagents should be directed to and will be fulfilled by the lead contact Elisabet Stener-Victorin (elisabet.stener-victorin@ki.se).

Materials availability

This study did not generate new unique reagents.

Data and code availability

- Raw data of F_1–F_3 through Mendeley Data: https://doi.org/10.17632/4vhx9dk4st.1.
- Raw sequencing reads of sncRNA-seq data of mouse sperm from F_1, F_2 and F_3 males are available in Sequence Read Archive database via accession number PRJNA743232.
- Raw sequencing reads of sncRNA-seq data of human serum are available in The European Genome-Phenome Archive (EGA) via accession number EGAS00001007079.
- For the Swedish register-based cohort, original data are held by the Swedish National Board of Health and Welfare and Statistics Sweden, and because of Swedish data privacy laws we cannot make the data publicly available. Any researcher can access the data by obtaining ethical approval from a regional ethical review board and thereafter asking the Swedish National Board of Health and Welfare and Statistics Sweden for the original data. However, aggregated data used in the analysis of this study are available from the authors upon reasonable request and with approved data sharing and data processing agreements in line with the General Data Protection Regulation. Further use of these data must be authorized by the local ethics committee regarding the merit of the project involved.
- Any additional information required is available from the elisabet.stener-victorin@ki.se upon request.

EXPERIMENTAL MODEL AND SUBJECT DETAILS

Ethical approvals

All procedures contributing to this work comply with the ethical standards of the relevant national and institutional committees on human experimentation and with the Helsinki Declaration of 1975, as revised in 2008. The register-based study was approved by the regional ethical review board in Stockholm, Sweden (diary number 2017/2423-31). The requirement for informed consent was waived because of the nature of this study, and the individuals included were not identifiable at any time. The case-control protocol was approved by the institutional review board of the University of Chile (Approval of Research Project No.032-2015). All parents signed informed consent, and boys gave their assent before any examination.

All animal experiments were approved by the Stockholm Ethical Committee for Animal Research (10798-2017) by the legal requirements of the European Community (SJVFS 2017:40) and the directive 2010/63/EU of the European Parliament on the protection of animals used for scientific purposes. Animal care and procedures were performed following guidelines specified by the European Council Directive and controlled by Comparative Medicine Biomedicum (KM-B), Karolinska Institutet, Stockholm, Sweden.

Register based study

The cohort used for this study gathers all births of children born in Sweden from 2006 up to 2016 and have been used to study the effects of antenatal use of commonly prescribed drugs and their effects on the pregnancy outcomes in the offspring up to December 2017. By using the unique Swedish personal identity number from mothers registered in the medical birth register was used to link the data with the: 1) the National Patient Registry (in- and specialist outpatient care), that contains visits to the specialist or hospitalizations since 1981, 2) the Causes of Death Registry and 3) the Drug Register that contains information about the dispensed medication since 2005. The main outcome was obesity in sons born to mothers with PCOS (with or without use of metformin during pregnancy). The two exposures evaluated were:1) maternal polycystic ovary syndrome, which was defined as having at least one diagnosis of PCOS (ICD-10: E282) in the Medical Birth Register during the study period or in the National Patient Registry (ICD-10:E282, ICD9:256E or ICD8:256,9) since 1981, and 2) use of metformin during pregnancy, that included those women with at least one prescription of metformin (ATC codes: A10BA02) during pregnancy. Thus, the risk of obesity in sons of mothers with PCOS was evaluated in the combinations of these two exposures i) All PCOS; ii) PCOS(+)/Met(−), and iii) PCOS(+)/Met(+). PCOS(−)/Met(−) was considered as reference. With this information, a total of 467,275 sons older than 2 years of age, born in Sweden between July 2006 and December 2015 and followed up to December 2017 were included. From them, 9,828 were born to a mother with diagnosed with PCOS. Of these, 165 mothers diagnosed with PCOS had at least one prescription of metformin discharged from pharmacy. Obesity diagnosis, identified in the National Patient Registry with the ICD-10 code E.66, was registered in 147 sons born to mother with PCOS (with or without use of metformin during pregnancy), and only 4 of them corresponded to mother with both exposures (PCOS(+)/Met(+)).

Human case-control study population

Seventy-eight 8 to 18-year-old pubertal sons born to PCOS mothers and 93 born to control women were included. This cohort includes subjects from a previous study from our group[7] and new subjects were recruited after that study. Boys were grouped as Tanner I, Tanner II-III and Tanner IV-V according to testicular volume. All boys were born from singleton pregnancies and were not taking any medication at the time of study. The boys were characterized through clinical, hormonal and metabolic measurements as previously described.[7] Mothers with PCOS were recruited from patients attending the Unit of Endocrinology and Reproductive Medicine, University of Chile, Santiago, Chile. PCOS mothers were diagnosed according to the National Institutes of Health Consensus Criteria and exhibited chronic oligomenorrhea or amenorrhea, hirsutism, and characteristic ovarian morphology of polycystic ovaries in ultrasound. Control mothers had a history of regular 28- to 32-day menstrual cycles, absence of hirsutism and other manifestations of hyperandrogenism and no history of infertility or pregnancy complications.

Animals

C57Bl/6J mice (3-week-old) were obtained from Janvier Labs (Le Genest-Saint-Isle, France). We generated and phenotype F_0 dams in detail.[6] To generate prenatal androgen exposed offspring, the CD and HFHS groups were randomly subdivided and injected daily subcutaneously (s.c.) in the inter-scapular area from E16.5 to E18.5 with 50 μL of a solution containing a mixture of 1) a mixture of 5 μL benzyl benzoate (B6630; Sigma-Aldrich) and 45 μL sesame oil (S3547; Sigma-Aldrich, St. Louis, Missouri, USA) i.e. vehicle, or 2) 250 μg DHT (5α androstane-17β-ol-3-one, A8380; Sigma-Aldrich, St. Louis, Missouri, USA) dissolved in a mixture of 5 μL benzyl benzoate and 45 μL sesame oil i.e. PNA by DHT. See key resource table for reagents and resources. Thus, four experimental lineages were studied, i.e., control diet+vehicle (control), control diet+ dihydrotestosterone (androgenized), high-fat, high-sucrose diet+vehicle (obese), and high-fat, high-sucrose diet+DHT (obese and androgenized) lineages.

Mouse-breeding scheme and feeding paradigm to generate F_1 to F_3 offspring

F_1, F_2, F_3 (Figure 2A) male offspring from CD + Veh, CD + DHT, HFHS+Veh and HFHS+DHT groups were weaned onto chow diet. A subset of F_1 male offspring was mated with unrelated females fed chow diet to generate F_2 male, and a subset of F_2 male offspring was mated with unrelated females fed chow diet to generate F_3 male. The remaining F_1, F_2 and F_3male offspring from both maternal and paternal lineages were subjected to phenotypic testing as described below. The exact number of litter and mice used for each procedure are given in the figure legends. To accurately ensure the variability within each group, offspring in each generation were randomly allocated for phenotypic testing or breeding.

METHOD DETAILS

Assessment of reproductive phenotype

Anogenital distance was measured in F_1, F_2 and F_3 male offspring at weaning time with vernier caliper. At 3-week to 15-week of age, body weight development was recorded weekly.

Assessment of metabolic phenotype

Body composition as total fat and lean masses were assessed by magnetic resonance imaging (EchoMRI-100 system) (EchoMRI LLC, Houston, TX) at 16 to 18-week of age. Metabolic cages (TSE PhenoMaster, TSE Systems GmbH, Thuringia, Germany) measured food intake, gas exchange, and spontaneous locomotor activity for three consecutive days. In metabolic cages animals were kept individually; the first day being considered an adaptation period (not analyzed) and 24-h readings were used for analysis after the adaption period. Parameters included were EE: indirect gas calorimetry and adjusted for total body mass and RER: VCO_2/VO_2 as the calculated ratio between volumes of CO_2 produced and O_2 consumed and were recorded for each mouse at 3-min intervals. Spontaneous locomotor activity was measured by recording interruptions of infrared light beams emitted along the x- and y axis of each cage (expressed in counts). An oral glucose tolerance test (OGTT) assessed glucose metabolism following 6-h fasting at 17 to 19-week of age. Oral gavage administered D-glucose (2 g/kg) and blood glucose was measured at 0 (before glucose administration) and 15, 30, 60, and 90-min (FreeStyle Precision, Abbott Diabetes Care Inc., UK). Blood was collected at 0 and 15-min for insulin measurement by tail bleeding.

Biochemical assessment of serum insulin and sex steroids

Serum insulin by an ELISA kit (Crystal Chem, Elk Grove Village, IL, USA) and serum testosterone, DHT, and androstenedione were measured by using GC-MS/MS assay[82] in F_1, F_2 and F_3 offspring.

Adipocyte size measurement

Epididymal adipose tissues were dissected and prepared as previously described for subcutaneous fat.[83] Tissue was sectioned with 5μm thickness, and 1 section was taken every 30 μm, in total 6 sections were taken for each sample. Three animals were used in each group. One to two representative images were taken per section with a light microscope at 20X magnification (Zeiss Axioplan, Germany). The adipocyte size was quantified using CellProfiler. Identified cells with an area >4000 AU or >0.97 eccentricities were removed. 20X representative images were taken with an inverted microscope (Olympus IX73, Olympus).

CellPress
OPEN ACCESS

Liver triglycerides quantification

Liver triglycerides (TG) were extracted and measured as described below: briefly, 100 mg of the liver was homogenized in the Tissue Lyzer (Qiagen, Hilden, Germany) for 3 min in 5% NP-40 alternative (492016; Merck KGaA, Darmstadt, Germany) with distilled water. Then the lysate was heated at 95 °C for 5 min in a heating block, until the samples became cloudy, then cooled down at room temperature. The previous step was repeated and centrifuged at 13,000 g × 2 min. The supernatant was removed, diluted 10 times, and analyzed using the TG kit Randox (TR210, Crumlin, United Kingdom) according to instructions from the manufacturer.

Testis AMH quantification

Testes AMH were extracted and measured as described below: briefly, 100 mg of the testis was homogenized in the Tissue Lyzer (Qiagen, Hilden, Germany) for 2 min in with 1X PBS. Then the lysate was stored at −80°C overnight. Then performed two freeze-thaw cycles to break the cell membranes. The lysate was centrifuged at 5000 g for 5 min. The supernatant was removed, and analyzed using the AMH ELISA kit (# OKEH00320, Aviva systems biology, San Diego, CA, USA) according to instructions from the manufacturer.

Liver oil red O staining

For oil red O staining, the liver was collected from F_1, F_2 and F_3 male offspring and immediately after collection flash-frozen the liver in liquid N_2. Then, a piece of liver was embedded in OCT (Sakura Finetek USA, Inc., Torrance, CA, USA)) on the mold and dropped into dry-ice chilled 2-Methylbutane (277258, Merck KGaA, Darmstadt, Germany). Optimal cutting temperature (OCT)-embedded tissues were sectioned into 10μm-thick sections and stained as describe.[84]

RNA isolation from testis and mRNA expression of mitochondrial genes

Total RNA was extracted by using TRI reagent (T9424, Sigma) from mouse testis. Reverse transcription was performed by using a High-Capacity RNA-to-cDNA kit (4387406, Applied Biosystems, Carlsbad, California, USA). Quantitative real-time PCR for gene analysis was performed in a ViiA 7 Real-Time PCR system thermal cycler with SYBR Green PCR Master Mix (both Applied Biosystems). The primer sequences (see key resource table) are *Tfam* **F**: ATTCCGAAGTGTTTTTCCAGCA; **R**: TCTGAAAGTTTTGCATCTGGGT; *Drp1* **F**: CAGGAATTGTTACGGTTCCCTAA; **R**: CCTGAATTAACTTGTCCCGTG; *Opa1* **F**: TGGAAAATGGTTCGAGAGTCAG; **R**: CATTCCGTCTCTAGGTTAAAGCG; *Guf1* **F**: GCTTTCTGATTGCTGGGATGA; **R**: TGCAAACACCATTGGTTTCGC. The relative gene expression was calculated using the comparative critical threshold (Ct) method. *Gapdh* was selected as the endogenous control and mRNA is presented as fold change.

Collection of organs and isolation of motile spermatozoa

At finalization, mice have fasted for 2 h before blood, spermatozoa, and tissue collection. Briefly, mice were anesthetized with isoflurane, and blood was collected through the axillar vein. The subcutaneous, epididymal adipose tissues and liver were quickly dissected on ice, snap-frozen, and stored at −80°C.

The cauda epididymis was dissected from the anesthetized animal and punctured in a Petri dish containing sperm isolation buffer (Earle's Balanced Salt Solution, 25 mM HEPES, 48.5 mM bovine serum albumin) pre-warmed to 37°C. Samples were transferred to a 14 mL round bottom tube overlaid with isolation buffer and subjected to a swim-up assay. Samples were incubated at 37°C at a 45-degree angle and the supernatant was harvested after 2 h.

Transmission electron microscopy (TEM) for mitochondrial morphology

Testis piece from F_1, F_2 and F_3 male offspring were fixed in 2.5% glutaraldehyde in 0.1 M phosphate buffer, pH 7.4 at room temperature for 30 min. Tissues were rinsed in 0.1 M phosphate buffer before post-fixation using 2% osmium tetroxide in 0.1 M phosphate buffer, pH 7.4 at 4°C for 2 h. The tissues were subsequently dehydrated in ethanol followed by acetone and finally embedded in LX-112. Ultrathin sections were prepared using a Leica EM UC7 (Leica Microsystems) and contrasted with uranyl acetate followed by Reynolds lead citrate. The sections were examined in a Hitachi HT 7700 Electron microscope (Hitachi High-Technologies) at 80kV and images acquired using a 2k x 2k Veleta CCD camera (Olympus Soft Imaging Solutions GmbH).

Small non-coding RNA library preparation

The total RNA was extracted from sperm in mice and from human serum (n = 9 sons of PCOS mothers and n = 9 sons of control mothers) by using the Trizol method. The quality and quantity of RNA were determined by Nanodrop-1000. 400 ng total RNAs are used for small RNA library construction by using NEBNext Multiplex Small RNA Library Prep Set for Illumina (NEB, E7300, USA). The PCR amplification products were purified by DNA Clean & Concentrator-5 Kit (Zymo Research, D4003) followed by size selection using PipPin Prep (Sage Science, CDP 3010) for F_1 and F_3 samples. For the samples of F_2 and human serum, size selection was performed using 6% polyacrylamide Gel (Life Technologies, EC6265BOX) with the instruction in NEB protocol. The quality of the library is checked by Bioanalyzer 2100 (Agilent Technologies, Inc.). The high-throughput sequencing is completed by Illumina HiSeq 2500 sequencer and 12 million reads of raw data are obtained.

sncRNA-seq data processing

Cutadapt version 1.9.1[85] was used to trim any remains of adaptor sequence (AGATCGGAAGAGCACACGTCTGAACTCCAGTCA) from sequenced reads. Only trimmed reads between 15 and 45 nucleotides, containing adaptor sequence, and with 80% of the nucleotides showing Illumina quality scores (Q-scores) >20 was retained. Average depth was 30.05 ± 1.14M reads per sample (min = 17.23M) in which at least 81.54% passed our initial filters. Next, trimmed reads were mapped to small RNA sequences using the analytical workflow Sports 1.0.[86] The databases used for biotype annotation include the UCSC genome GRCm38 for mouse and GRCh38 for human, and the mouse and human fasta sequences from the following databases: miRbase 22.1, rRNA from NCBI/Nucleutide, GtRNAdb, piRBase and piRNA bank, ensembl (release-89) ncRNA, and rfam 12.3. We sequentially mapped the reads to miRNA, rRNA, tRNA, piRNA and other non-coding RNAs. For tsRNA annotation, the sports pipeline has pre-processed the tRNA sequences via removal of predicted introns in the tRNA genes, addition of CCA sequence to the 3′ ends of the tRNAs, as well as addition of a single base G to the 5′ ends of histidine tRNAs. To balance the trade-off between information leakage due to RNA base modification and accuracy of mapping, we first set the mismatch for sequence acceptance to be zero for all the subtypes. Afterward, we took out the piRNA and no-annotation sequences that were not mapped to the genome. With one mismatch allowed, these sequences were re-annotated as rsRNA or tsRNA if they were mapped to the rRNA or tRNA. According to the origin loci in the parent tRNAs, the tsRNA were annotated as 5′ tsRNA, 3′tsRNA, 3′ tsRNA-CCA end, and internal tsRNA (i tsRNA). For rsRNA annotation, to avoid repeated counting caused by rRNA sequence redundancy, the rsRNA were annotated based on the size of the rRNA source in a small to large sequence in terms of the Svedberg unit. For example, a small RNA would be identified as 18s derived rsRNA but not 45s RNA derived rsRNA if it was simultaneously mapped to 18s and 45s RNAs.

Differentially expressed gene analyses

We performed differential expression of small RNAs based on ID annotation for miRNA and clusters for other biotypes. For miRNA sequences, we sum up the expressions based on the miRNA ID. For the other small RNA biotypes, we used UPARSE, a method for generating clusters (OTUs) from next-generation sequencing reads of small RNAs with identical sequences,[87] to assign the sequences into clusters and then sum up the expression based on the clusters. To decrease the noise, we first filtered out the genes with LogMean RPM <1.[88,89] Next, we used standard edgeR[90] workflow for DEG analyses, with a Log2 fold change >1 and p-value <0.05 considered as candidate DEGs. For log2 fold change calculation and heatmap, expression values are normalized by library depth into Reads Per Million Reads (RPM).

Identification of common DEmiRNAs in women with PCOS and in sons of PCOS women

We obtained the DEmiRNA in serum, whole blood, plasma, or serum exosomes between women with PCOS and controls from published paper,[38,59–66] and then identified the common ones with the DEmiRNAs in sons of PCOS women.

Human and mouse DEsncRNAs alignment

A large number of rsRNAs have been reported to target genes through Argonaute pathway,[91] in which the seeding sequence for smallRNA-mRNA binding is primarily dependent on the seeding sequence starting from the 5′ end second base in the small RNA. Since we focused on target gene prediction through Argonaute pathway, we identified homological alignment hits through blastn[92] with the criteria that the query and subject DEsncRNAs have identical sequences starting from their second bases at the 5′ end and ending close to the 3′ ends to cover larger than 90 percent of their total lengths.

Target gene prediction and gene ontology analyses

The target genes for miRNA are annotated in miRBase22.1, in which the predicted targets with scores larger than 90 are used. For the piRNA target mRNAs, the information comes from piRBase.[93] Gene Ontology enrichment is carried out by metascape.[94]

QUANTIFICATION AND STATISTICAL ANALYSIS

In the register-based cohort study, association between maternal PCOS, with or without use of metformin during pregnancy, and obesity in their sons was estimated as HRs and 95% CIs using a multivariable Cox proportional-hazard model. Childhood obesity in sons were assessed in a main analysis including i) women with PCOS that did not use of metformin during pregnancy and in a sub-analysis including ii) women with PCOS, who used metformin during pregnancy. All analysis was performed with PCOS-/Met-as the reference. The covariates in the adjusted model were: maternal age at delivery stratified as <25, 25–29, 30–34 and ≥35; maternal BMI stratified as <18.5, 18.5–24.9, 25–29.9 and ≥30; parity (multiparous/, nulliparous), cigarette consumption at enrollment (yes/no); assisted reproduction (yes/no); size for gestational age (adequate, small or large); preterm birth (yes/no); apgar <07 at 5 min; cesarean section and diabetes (gestational diabetes, diabetes mellitus or use of metformin during pregnancy). Considering the potential dependence between the observations (many children from the same mother), robust standard errors was considered in the analysis. As obesity should not be diagnosed before the age of two,[95] the follow-up was calculated from two years of age until the first reported occurrence of the outcome, death, or end of study period (December 2017), whichever occurred first.

CellPress
OPEN ACCESS

Moreover, to define how maternal body weight modify the associations between maternal PCOS and childhood obesity we performed a sub-analysis considering: i) women with a BMI <25 and ii) women with a BMI $\geq$25. The analysis was done if at least 5 cases are identified with the outcome in the main or sub-analysis.

The sample size in the mice experiments was based on differences in anogenital distance in the control and androgenized lineage in our previous studies.[6,96] Nine animals per group were required to detect a mean difference in anogenital distance of 40.6% with a standard deviation (SD) of 0.1, a significance level of 0.05, and a power of 0.8. All data are presented as mean ± s.e.m, SD or as median and range. In the transgenerational experiments, to control for the dependency of pups coming from the same litter, in each generation we performed multilevel model statistical analyses as previously described.[97] In brief, we used linear mixed-effects models (lmer, lme4, R 4.1.1) with two different approaches. First, in non-repeated measure, comparisons were made between each lineage (fixed effects) with litter ID as random factor, except for adipocyte size in which mouse ID was included as random factor. In repeated measures i.e., body weight development, OGTT, metabolic cages, 2 fixed effects were included i.e., lineages and time/weeks of measure with mouse ID and litter ID as random factor. Then statistical significance was calculated based on an analysis of deviance (ANODE, R package car). Differences were considered statistically significant at $p < 0.05$. Statistical analyses in the register-based study were performed using Stata statistical software version 14.0 (Stata Corps, Texas, USA), and in the case-control study by GraphPad Prism 8 (GraphPad Software Inc., CA, USA) and mouse data using the R software 4.1.1.

Cell Reports Medicine, Volume *4*

Supplemental information

Transgenerational transmission of reproductive and metabolic dysfunction in the male progeny of polycystic ovary syndrome

Sanjiv Risal, Congru Li, Qing Luo, Romina Fornes, Haojiang Lu, Gustaw Eriksson, Maria Manti, Claes Ohlsson, Eva Lindgren, Nicolas Crisosto, Manuel Maliqueo, Barbara Echiburú, Sergio Recabarren, Teresa Sir Petermann, Anna Benrick, Nele Brusselaers, Jie Qiao, Qiaolin Deng, and Elisabet Stener-Victorin

Cell Reports Medicine

Supplemental Information

Transgenerational transmission of reproductive and metabolic dysfunction in the male progeny of polycystic ovary syndrome

Sanjiv Risal, Congru Li, Qing Luo, Romina Fornes, Haojiang Lu, Gustaw Eriksson, Maria Manti, Claes Ohlsson, Eva Lindgren, Nicolas Crisosto, Manuel Maliqueo, Barbara Echiburú, Sergio Recabarren, Teresa Sir Petermann, Anna Benrick, Nele Brusselaers, Jie Qiao, Qiaolin Deng, Elisabet Stener-Victorin

Supplemental Data to:

Transgenerational transmission of reproductive and metabolic dysfunction in the male progeny of polycystic ovary syndrome

Sanjiv Risal[1,13], Congru Li[1,2,13], Qing Luo[1,13], Romina Fornes[1,3,13], Haojiang Lu[1], Gustaw Eriksson[1], Maria Manti[1], Claes Ohlsson[4,5], Eva Lindgren[1], Nicolas Crisosto[6,7], Manuel Maliqueo[6], Barbara Echiburú[6], Sergio Recabarren[8], Teresa Sir Petermann[6], Anna Benrick[9,10], Nele Brusselaers[3,11], Jie Qiao[2], Qiaolin Deng[1, 12, 14], Elisabet Stener-Victorin[1, 15]

[1] Department of Physiology and Pharmacology, Karolinska Institutet, Stockholm, Sweden

[2] Center of Reproductive Medicine, Department of Obstetrics and Gynecology, Peking University Third Hospital, Beijing 100191, China

[3] Department of Microbiology, Tumor and Cell Biology, Karolinska Institutet, Stockholm, Sweden

[4] Centre for Bone and Arthritis Research, Department of Internal Medicine and Clinical Nutrition, Institute of Medicine, Sahlgrenska Academy, University of Gothenburg, Gothenburg, Sweden

[5] Department of Drug Treatment, Region Västra Götaland, Sahlgrenska University Hospital, Gothenburg, Sweden

[6] Endocrinology and Metabolism Laboratory, West Division, School of Medicine, University of Chile, Carlos Schachtebeck 299, Interior Quinta Normal, Santiago, Chile

[7] Endocrinology Unit, Department of Medicine, Clínica Alemana de Santiago, Faculty of Medicine, Clinica Alemana, Universidad del Desarrollo, Santiago, Chile

[8] Laboratory of Animal Physiology and Endocrinology, Faculty of Veterinary Sciences, University of Concepción, Chillán, Chile

[9] Department of Physiology, Sahlgrenska Academy, University of Gothenburg, Gothenburg, Sweden

[10] School of Health Sciences, University of Skövde, Skövde, Sweden

[11] Global Health Institute, Antwerp University, Antwerp, Belgium

[12] Center for Molecular Medicine, Karolinska University Hospital, Stockholm, Sweden

[13] **These authors contributed equally.**

[14] **Corresponding author**
Qiaolin Deng: qiaolin.deng@ki.se
Karolinska Institutet, Department of Physiology and Pharmacology
Center for molecular medicine
171 77 Stockholm, Sweden

[15] **Corresponding author and lead contact**
Elisabet Stener-Victorin: elisabet.stener-victorin@ki.se
Karolinska Institutet, Department of Physiology and Pharmacology
171 77 Stockholm, Sweden

Table S1. Maternal and pregnancy characteristics of women with and without polycystic ovary syndrome (PCOS) and exposure to metformin (Met) and perinatal characteristics of their sons between 2006 to 2016 in a Swedish register-based cohort study. **Related to figure 1.**

	**Control**		**PCOS**		**PCOS+/Met-**		**PCOS+/Met+**	
	**n**	**%**	**n**	**%**	**n**	**%**	**n**	**%**
	**457,447**	**%**	**9,828**	**%**	**9,663**		**165**	
Maternal age, in years								
< 25	66,280	14.5	1,341	13.6	1,325	13.7	16	9.7
25-29	135,271	29.6	3,262	33.2	3,210	33.2	52	31.5
30-34	156,353	34.2	3,425	34.8	3,372	34.9	53	32.1
≥ 35	99,543	21.8	1,800	18.3	1,756	18.2	44	26.7
Maternal body mass index, in kg/m^2								
<20	47,168	10.3	736	7.5	731	7.6	5	3.0
20-24.9	239,774	52.4	3,780	38.5	3,751	38.8	29	17.6
25-29.9	114,682	25.1	2,730	27.8	2,689	27.8	41	24.8
≥30	55,823	12.2	2,582	26.3	2,492	25.8	90	54.5
Parity								
Multiparous	255,389	55.8	4,616	47.0	4,543	47.0	73	44.2
Primiparous	202,058	44.2	5,212	53.0	5,120	53.0	92	55.8
Cigarrette consumption								
yes	28,249	6.2	583	5.9	4,543	33.3	16	9.7
no	429,198	93.8	9,245	94.1	9,096	66.7	149	90.3
Assisted reproduction								
yes	12,739	2.8	1,086	11.1	1,069	11.1	17	10.3
no	444,708	97.2	8,742	88.9	8,594	88.9	148	89.7
Diabetes								
yes	8,935	2.0	528	5.4	490	5.1	38	23.0
no	448,512	98.0	9,300	94.6	9,173	94.9	127	77.0
Size for gestational age								
Adequate for gestational age	432,251	94.5	9,137	93.0	8,990	93.0	147	89.1
Small for gestational age	10,025	2.2	209	2.1	204	2.1	5	3.0
Large for gestational age	15,171	3.3	482	4.9	469	4.9	13	7.9
Cesarean section								
yes	78,626	17.2	2,081	21.2	2,034	21.0	47	28.5
no	378,821	82.8	7,747	78.8	7,629	79.0	118	71.5

Preterm birth								
yes	21,217	4.6	622	6.3	612	6.3	10	6.1
no	436,230	95.4	9,206	93.7	9,051	93.7	155	93.9
Apgar <07 at 5 minutes								
yes	5,229	1.1	153	1.6	151	1.6	2	1.2
no	452,218	98.9	9,675	98.4	9,512	98.4	163	98.8

Table S2. Clinical. endocrine and metabolic features of sons of control and PCOS women in the Chilean case-control study. **Related to figure 1.**

	Tanner I			Tanner II - III			Tanner IV - V		
	Control	PCOS	*P*	Control	PCOS	*P*	Control	PCOS	*P*
Age (y)	9.0 (8.3 - 9.6) (20)	9.1 (8.4 - 9.6) (23)	0.541	10.3 (9.8 - 11.8) (31)	10.8 (10.3 - 11.6) (26)	0.442	14.6 (13.2 - 16.4) (42)	15.3 (13.7 - 16.7) (29)	0.289
Weight (kg)	35.6 (26.6 - 38.7) (20)	33.5 (30 - 42.0) (23)	0.846	39.1 (34.6 - 48.0) (31)	40.8 (35.9 - 54.6) (26)	0.400	60.1 (52.9 - 77.4) (42)	65.1 (56.5 - 78.5) (29)	0.281
Height (m)	1.3 (1.3 - 1.4) (20)	1.4 (1.3 - 1.4) (23)	0.573	1.4 (1.4 - 1.5) (31)	1.4 (1.4 - 1.5) (26)	0.500	1.7 (1.6 - 1.7) (42)	1.7 (1.6 - 1.7) (29)	0.091
BMI (kg/m^2)	19.8 (16.4 - 22.6) (20)	18.7 (17.3 - 22.4) (23)	0.850	20.2 (18.2 - 23.1) (31)	21.0 (18.4 - 24.8) (26)	0.421	22.9 (19.8 - 26.4) (42)	24.5 (20.2 - 27.1) (29)	0.533
Z-Score BMI	1.6 (-0.1 - 2.4) (20)	1.4 (0.4 - 2.6) (23)	0.581	1.5 (0.6 - 2.1) (31)	1.6 (0.8 - 2.5) (26)	0.330	1.2 (0.5 - 1.8) (42)	1.5 (0.3 - 2.1) (29)	0.648
Obesity (%)	40.0	39.1	1.000	29.0	34.6	0.777	16.7	24.1	0.547
Waist (cm)	63.3 (59.3 - 68) (20)	62.5 (59.6 - 70) (20)	0.968	66.0 (61.0 - 75.0) (31)	70.5 (65.8 - 79.3) (26)	0.111	74.5 (70.0 - 84.3) (42)	79.0 (72.8 - 87.5) (29)	0.097
Hip (cm)	71.0 (62.1 - 77.6) (20)	68 (64.3 - 77.3) (20)	0.560	72.0 (65.0 - 82.0) (31)	74.8 (69.9 - 84) (26)	0.431	84.0 (80.0 - 92.6) (42)	87.0 (80.8 - 93.5) (29)	0.264
Endocrine parameters									
SHBG (nmol/l)	56.5 (36.7 - 85.8) (20)	47.3 (35.2 - 66.7) (22)	0.268	45.9 (31.1 - 69.1) (30)	50.0 (29.7 - 72.5) (25)	1.000	23.0 (16.5 - 39.6) (41)	22.6 (16.6 - 30.5) (28)	0.582
LH (mIU/ml)	0.1 (0.1 - 0.2) (20)	0.1 (0.1 - 0.9) (20)	0.245	0.8 (0.2 - 2.5) (28)	1.8 (0.2 - 3.4) (24)	0.533	5.1 (2.8 - 7.8) (42)	4.5 (3.4 - 6.5) (27)	0.623
FSH (mIU/ml)	0.3 (0.1 - 0.7) (20)	0.5 (0.2 - 0.7) (21)	0.375	0.4 (0.1 - 0.9) (29)	1.0 (0.3 - 2.2) (24)	**0.038**	1 (0.3 - 2.2) (42)	0.9 (0.4 - 3.6) (27)	0.503
A4 (ng/ml)	0.3 (0.2 - 0.6) (20)	0.5 (0.3 - 0.7) (21)	0.111	0.6 (0.3 - 0.9) (29)	0.7 (0.6 - 1.1) (25)	0.070	1.3 (0.9 - 1.9) (42)	1.6 (1.2 - 2.4) (28)	**0.039**
T (ng/ml)	0.1 (0.1 - 0.2) (20)	0.1 (0.1 - 0.2) (21)	0.102	0.2 (0.1 - 0.9) (30)	0.3 (0.2 - 1.2) (25)	0.314	3.7 (2.9 - 4.7) (40)	4.0 (3.1 - 6.1) (27)	0.184
AMH (pmol/L)	528.5 (434.4 - 780.7) (20)	549.4 (458.4 - 726.3) (20)	0.626	411.6 (200.1 - 528.6) (30)	440.8 (154.7 - 514.5) (24)	0.287	67.8 (42.6 - 119.6) (41)	62.6 (39.3 - 119.8) (28)	0.678
FAI	0.7 (0.3 - 1.1) (20)	1.0 (0.8 - 1.7) (21)	0.054	2.4 (0.9 - 5.4) (30)	2.9 (1.4 - 12.3) (25)	0.917	44.9 (32.0 - 92.8) (40)	68.1 (52.5 - 86.7) (27)	0.135
Metabolic parameters									
Fating glucose (mg/dl)	80.0 (77.3 - 85) (20)	84.0 (78.0 - 89.0) (23)	0.215	84.0 (76.0 - 90.0) (31)	87 (80.8 - 93.5) (26)	0.111	84.5 (77.0 - 92.0) (42)	84.0 (79.0 - 87.0) (29)	0.680
Fasting insulin (uIU/ml)	7.9 (5.4 - 12.4) (20)	7.0 (5.2 - 11.9) (23)	0.715	9.5 (7.2 - 13.9) (31)	9.3 (7.1 - 12.7) (26)	0.949	12.8 (9.2 - 18) (41)	12.8 (7.5 - 18.5) (29)	0.839
HOMA	1.5 (1.1 - 2.6) (20)	1.6 (1.1 - 2.5) (23)	0.903	2.0 (1.5 - 2.9) (31)	1.9 (1.5 - 2.7) (26)	0.898	2.7 (1.9 - 3.8) (41)	2.6 (1.7 - 3.6) (29)	0.716
AUC glucose (mg/dl/2-h)	11340 (10413.8 - 13196.3) (20)	11865 (10365 - 14280) (23)	0.466	12585 (11370 - 14700) (31)	12967.5 (11782.5 - 14602.5) (26)	0.564	12180 (10927.5 - 13882.5) (33)	12412.5 (11036.3 - 13301.3) (20)	0.761
AUC insulin (uIU/ml/2-h)	4949.1 (2328.5 - 8004.2) (20)	4341.3 (2990.3 - 5933.6) (23)	0.715	4827.3 (2946.6 - 9672.9) (31)	5916.8 (4032.7 - 10748.9) (26)	0.543	7068 (5124.6 - 12910.7) (33)	7975.3 (5006 - 11510) (20)	0.971
Triglycerides (mg/dl)	107.5 (86.3 - 131.8) (20)	104.0 (81.0 - 137.0) (23)	0.456	106.0 (82.0 - 141.0) (31)	125.5 (88.5 - 150.8) (26)	0.247	109.5 (77.3 - 138.3) (42)	97 (83.0 - 138.0) (29)	0.478
Cholesterol (mg/dl)	141.0 (133.3 - 163.5) (20)	155.0 (129.0 - 176.0) (23)	0.348	140.0 (130.0 - 159.0) (31)	164.0 (141.8 - 179.0) (26)	**0.008**	118.5 (103.5 - 137.3) (42)	134.0 (118.0 - 164.0) (29)	**0.015**
HDL-Cholesterol (mg/dl)	43.8 (39.4 - 52.8) (20)	40.1 (36.7 - 49.7) (23)	0.113	41.8 (37.2 - 45.1) (31)	42.0 (38.0 - 52.0) (26)	0.665	36.1 (29.5 - 43.0) (42)	38.6 (33.5 - 44.0) (29)	0.390
LDL-Cholesterol (mg/dl)	74.1 (57.4 - 82.2) (20)	88.9 (70.1 - 107.5) (23)	0.084	79.6 (57.6 - 92.9) (31)	92.6 (78.5 - 103.4) (26)	**0.037**	54.0 (45.4 - 71.7) (41)	74.6 (56.3 - 101.1) (29)	**0.005**

Data are expressed as median (p25 - p75). Brackets show the number of observations. Differences were calculated by Student t' test or Mann Whitney test depending on normal distribution. AUC: Area Under the Curve; BMI: Body mass index; FAI: free androgen index.

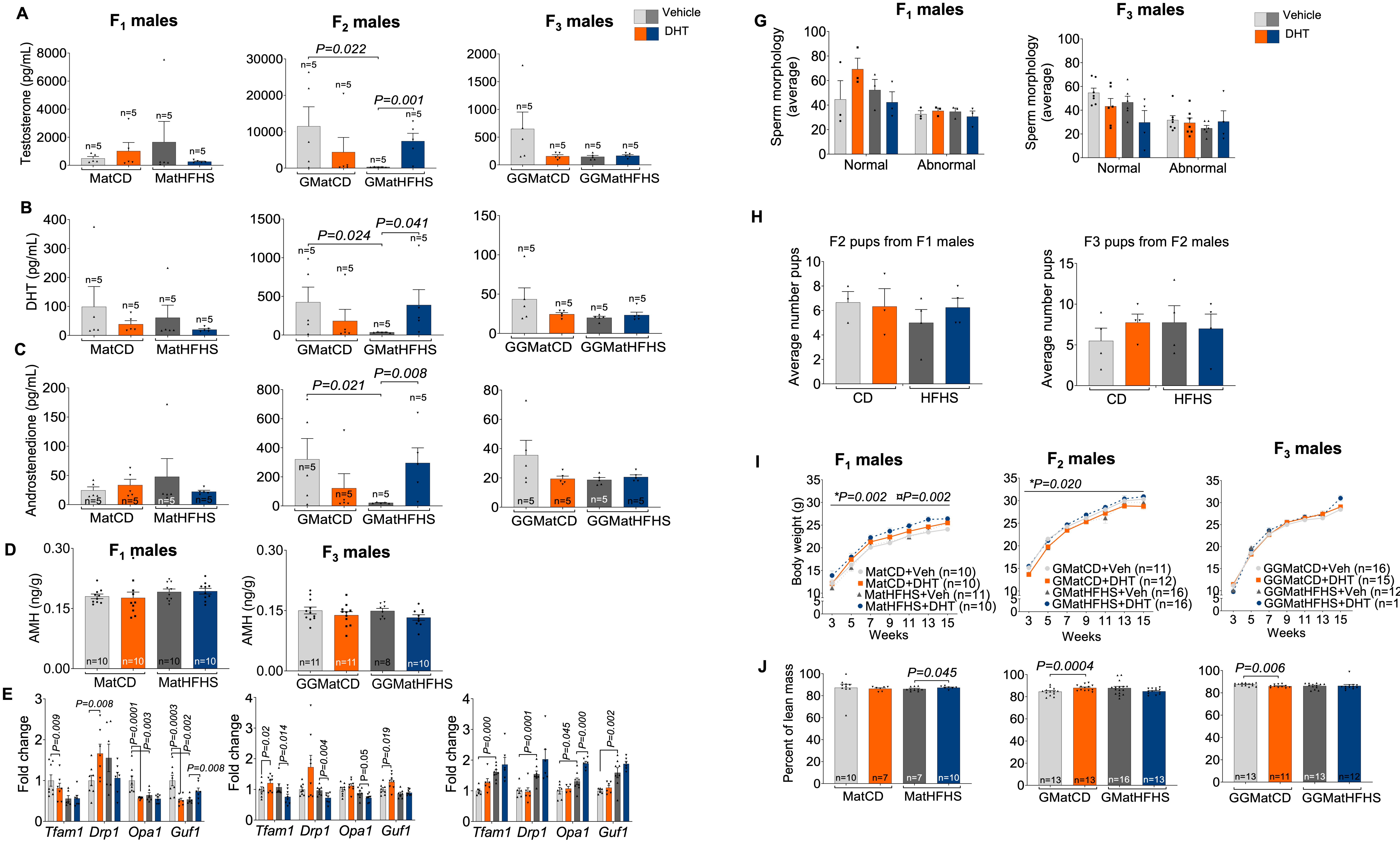

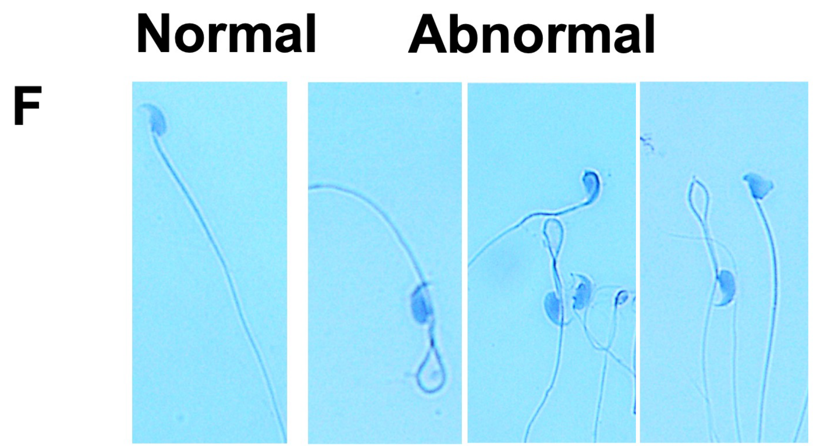

Supplemental Figure 1. Maternal obesity and prenatal androgen do not affect serum steroid levels and sperm morphology, mitochondrial gene expression, and body weight in male offspring. Related to figure 2.
Serum (A) testosterone, (B) dihydrotestosterone and (C) androstenedione levels measured by gas-chromatography mass spectrometry (GC-MS/MS) in F1, F2 and F3 male offspring.
(CD+Veh (F1 n=5, litters=5; F2 n=5, litters=4; F3 n=5, litters=4), CD+DHT (F1 n=5, litters=5; F2 n=5, litters=4, F3 n=5, litters=4), HFHS+Veh (F1 n=5, litters=5; F2 n=5, litters=4, F3 n=5, litters=4), HFHS+DHT (F1 n=5, litters=5; F2 n=5, litters=4; F3 n=5, litters=4). (D) mRNA expression levels of mitochondrial genes: Tfam1, Drp1, Opa1, and Guf1 in testis of F1, F2, and F3 male offspring.
CD+Veh (F1 n=8, litters=5; F2 n=8, litters=4 F3 n=6, litters=3), CD+DHT (F1, n=7, litters=5; F2 n=8, litters=4, F3 n=6, litters=4), HFHS+Veh (F1 n=6, litters=5; F2 n=7, litters=4, F3 n=8, litters=4), HFHS+DHT (F1, n=7, litters=5; F2 n=8, litters=4;, F3 n=6, litters=4).
(E) Representative images of normal and abnormal sperm morphology considered for analysis. (F) Average number of normal and abnormal sperm (expressed in percentages) of F1 and F3 male offspring.
(G) Average total number of pups sired by F1 and F2 males. Body weight development in (H) F1, F2, and F3 adult male offspring from 3 to15 weeks of age.
CD+Veh (F1 n=10, litters=5; F2 n=11, litters=4; F3 n=16, litters=4), CD+DHT (F1 n=10, litters=5; F2 n=12, litters=4; F3 n=15, litters=4), HFHS+Veh, (F1 n=11, litters=5; F2 n=16, litters=4; F3 n=12, litters=4), HFHS+DHT (F1 n=10, litters=7; F2 n=16, litters=4; F3 n=13, litters=4).
(i) Percent lean mass normalized with body weight (grams) in F1, F2, and F3 adult male offspring.
CD+Veh (F1 n=10, litters=5; F2 n=13, litters=4; F3 n=13, litters=4), CD+DHT (F1 n=7, litters=5; n=13; F2 litters=4, F3 n=11, litters=4), HFHS+Veh (F1 n=10, litters=5; F2 n=16, litters=4, F3 n=13, litters=4), HFHS+DHT, (F1 n=7, litters=4. F2 n=13, litters=4; F3 n=12, litters=4).
Lineage: CD+Veh = maternal control diet + vehicle exposure; CD+DHT = maternal control diet + dihydrotestosterone exposure; HFHS+Veh = maternal high fat high sucrose diet + vehicle exposure.
Comparison between the groups were performed using linear mixed-effects models, non-repeated measures (ANODE, R package car) except for panel H) linear mixed-effects models with repeated measure was used.
*=CD+Veh vs CD+DHT; ¤=HFHS+Veh vs HFHS+DHT. Each dot represents one offspring mouse. All data are presented as mean ± s.e.m.

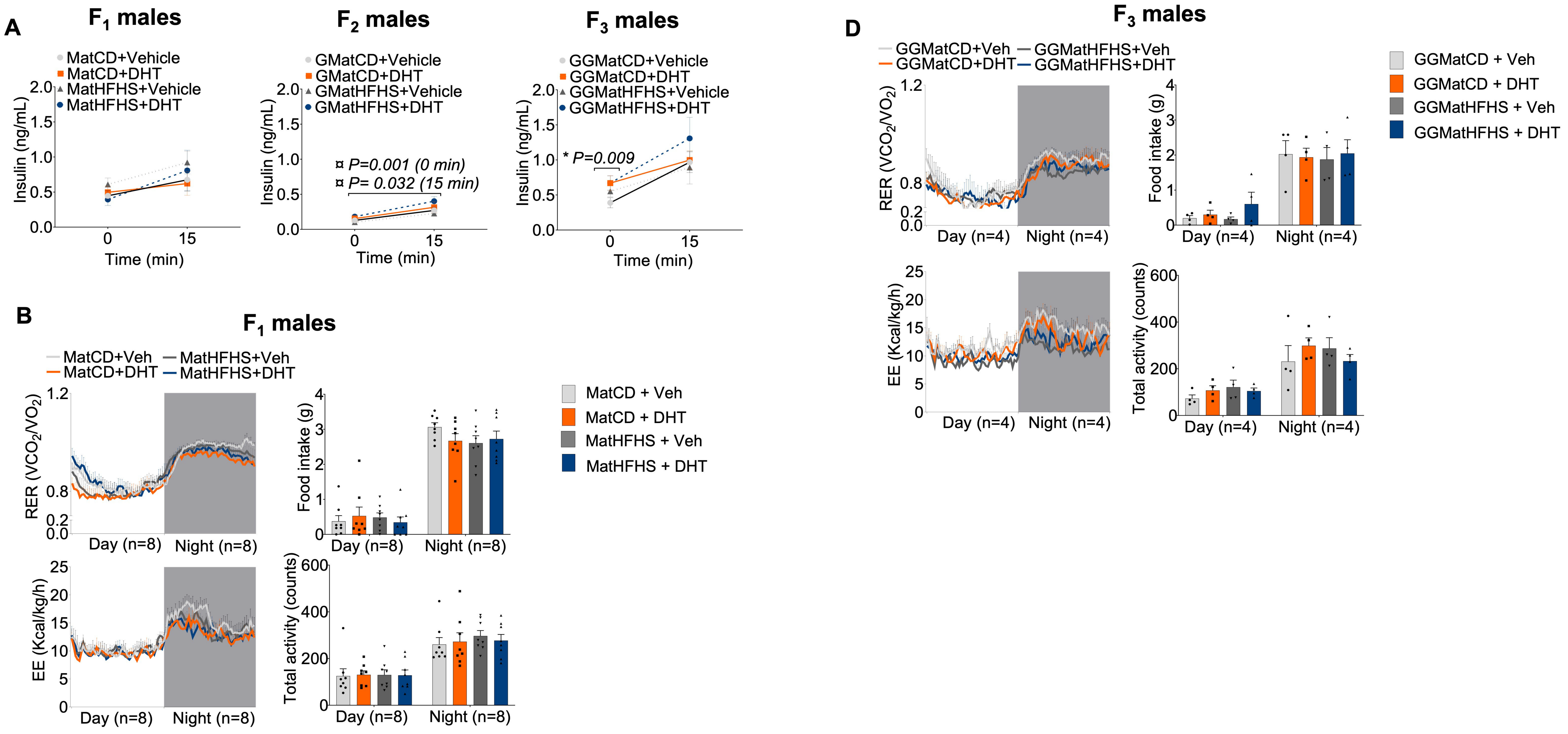

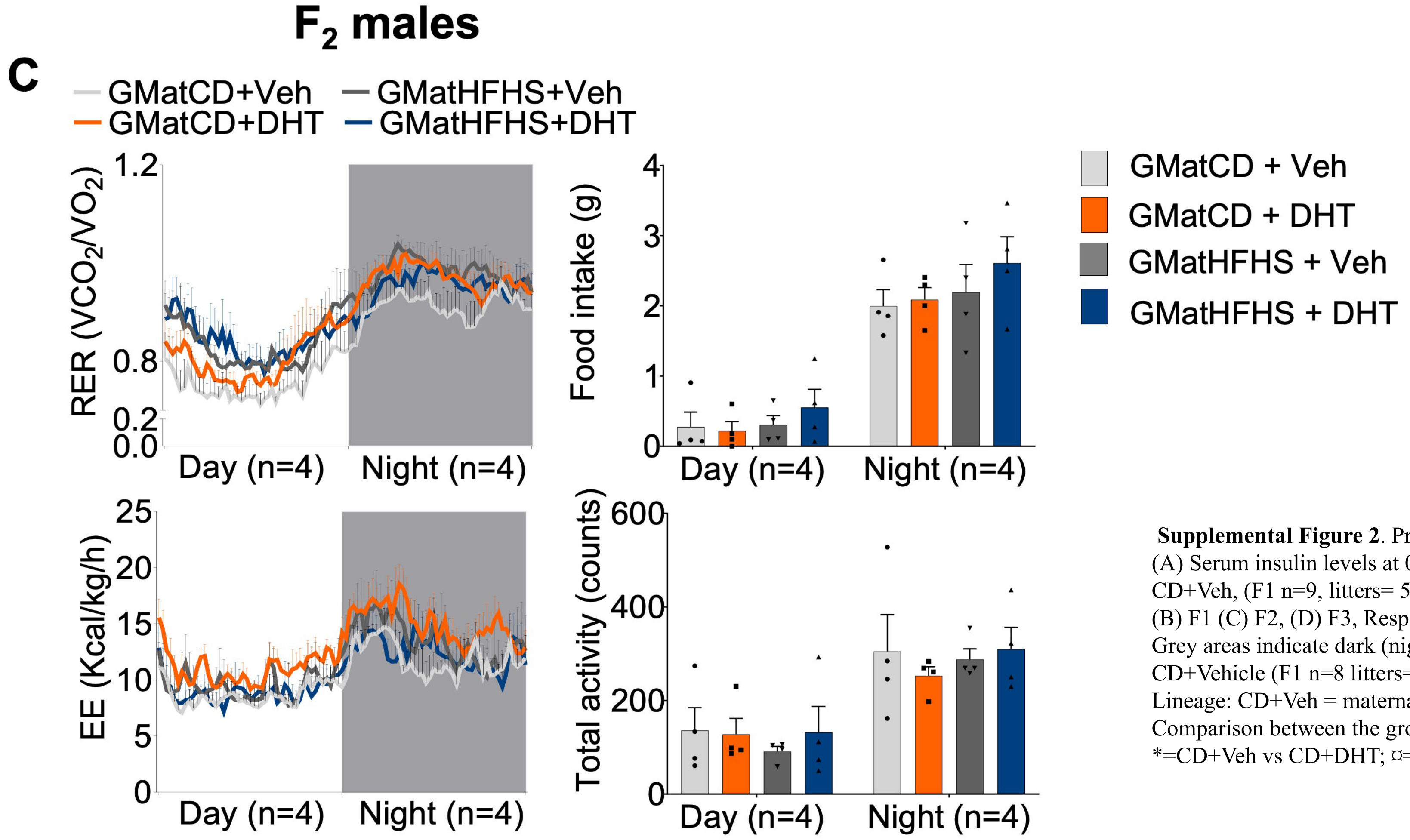

Supplemental Figure 2. Prenatal androgen and maternal obesity exposure cause transgenerational impaired energy balance in male offspring. Related to figure 3.
(A) Serum insulin levels at 0 and 15 minutes before and after oral glucose challenge.
CD+Veh, (F1 n=9, litters= 5; F2 n=9, litters=4; F3 n=12, litters=4), CD+DHT (F1 n=7, litters=4, F2 n=5, litters=4, F3 n=8, litters=4), HFHS+Veh (F1 n=9 litters=5; F2 n=9, litters=4; F3 n=12, litters=4) HFHS+DHT (F1 n=7, litters=4; F2 n=5, litters=4. F3 n=8, litters=4).
(B) F1 (C) F2, (D) F3, Respiratory exchange ratio (RER; VCO2/VO2) and energy expenditure (EE) was measured by indirect calorimetry by using the TSE system in F1 to F3 adult male offspring.
Grey areas indicate dark (night) periods (6 PM to 6 AM). Bar graph represents RER, EE, food intake, and total activity average values during day and night.
CD+Vehicle (F1 n=8 litters=4; F2 n=4, litters= 4; F3 n=4, litters=4), CD+DHT (F1 n=8, litters=4; F2 n=4, litters=4; F3 n=4, litters=4), HFHS+Veh (F1 n=8, litters=4; F2 n=4, litters=4; F3 n=4, litters=4), and HFHS+DHT (F1 n=8, litters=5; F2 n=4, litters=4; F3 n=4, litters=4).
Lineage: CD+Veh = maternal control diet + vehicle exposure; CD+DHT = maternal control diet + dihydrotestosterone exposure; HFHS+Veh = maternal high fat high sucrose diet + vehicle exposure.
Comparison between the groups were performed using linear mixed-effects models, non-repeated measures (ANODE, R package car) except for panel H) linear mixed-effects models with repeated measure was used.
*=CD+Veh vs CD+DHT; ¤=HFHS+Veh vs HFHS+DHT. Each dot represents one offspring mouse. All data are presented as mean ± s.e.m.

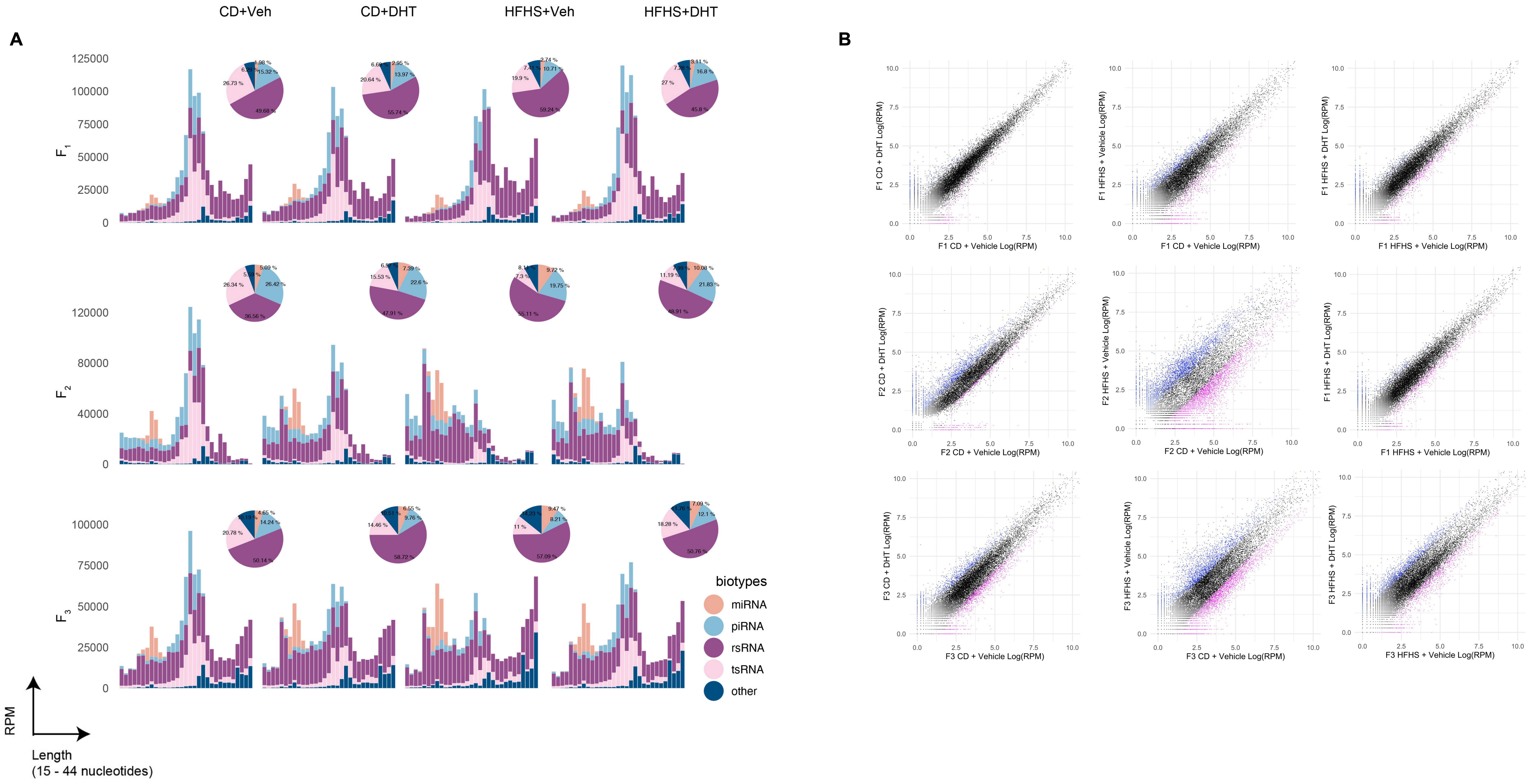

Supplemental Figure 3. Size distribution and expression of sncRNAs in sperm. Related to figure 4.
(A) The size distribution of miRNA, piRNA, rsRNA, tsRNA and other biotypes in different lineages in F1 and F3 male. Average RPM (reads per million) values in bar plots and RPM proportion (%) in pie charts.
(B) Scatterplot analysis showing average RPM values of different lineages as indicated in x-axis and y-axis for differentially expressed sncRNA. Down (magenta) and up (blue) regulated sncRNAs.
CD+Veh (F1 n=4, litters=4; F2 n=4, litters= 4; F3 n=4, litters=4), CD+DHT (F1 n=3, litters=3; F2 n=4, litters=4; F3 n=3, litters=3), HFHS+Veh (F1 n=4, litters=4; F2 n=4, litters=4; F3 n=4, litters=4), and HFHS+DHT (F1 n=4, litters=4; F2 n=4, litters=4; F3 n=4, litters=4).
